# The equal effectiveness of different defensive strategies

**DOI:** 10.1038/srep13049

**Published:** 2015-08-12

**Authors:** Shuang Zhang, Yuxin Zhang, Keming Ma

**Affiliations:** 1 State Key Laboratory of Urban and Regional Ecology, Research Center for Eco-Environmental Sciences, Chinese Academy of Sciences, Beijing, 100085, P. R., China

## Abstract

Plants have evolved a variety of defensive strategies to resist herbivory, but at the interspecific level, the relative effectiveness of these strategies has been poorly evaluated. In this study, we compared the level of herbivory between species that depend on ants as indirect defenders and species that rely primarily on their own direct defenses. Using a dataset of 871 species and 1,405 data points, we found that in general, ant-associated species had levels of herbivory equal to those of species that are unattractive to ants; the pattern was unaffected by plant life form, climate and phylogenetic relationships between species. Interestingly, species that offer both food and nesting spaces for ants suffered significantly lower herbivory compared to species that offer either food or nesting spaces only or no reward for ants. A negative relationship between herbivory and latitude was detected, but the pattern can be changed by ants. These findings suggest that, at the interspecific level, the effectiveness of different defensive strategies may be equal. Considering the effects of herbivory on plant performance and fitness, the equal effectiveness of different defensive strategies may play an important role in the coexistence of various species at the community scale.

Herbivory is the most important pathway for energy flow from autotrophic plants to higher trophic levels^1^. It has profound effects on plant community structure and dynamics, nutrient cycling, and the primary productivity of an ecosystem^2^,^3^,^4^. The “arms race” between plants and herbivores is one of the main drivers of the diversification of angiosperms and insect herbivores as well as the coexistence of species in nature^5^,^6^. Plants have evolved a range of defensive strategies against herbivores that reduce herbivory and enhance plant fitness effectively^7^,^8^. Different species also have different defensive strategies^7^,^8^, but, at the interspecific level, the relative effectiveness of these strategies has been poorly evaluated.

Plant defensive strategies can be classified into two types: direct and indirect^9^,^10^. Direct defense includes physical traits, such as thorns, trichomes, and secondary metabolites produced by the plant itself, such as tannin and latex^11^. In many cases, direct defense can be induced, and thus the expression of direct defensive traits often varies with the actual level of herbivory^9^,^10^,^11^. Plants can also attract the enemies of herbivores through the emission of volatile organic compounds (VOCs) or by offering other rewards for indirect defense^9^. Ants play an important role in the indirect defense of plants, and protective ant-plant interactions have been used as model systems in ecology and evolutionary studies^12^,^13^,^14^. At the intraspecific level, numerous studies have confirmed the positive effects of ants on plants, especially their strong anti-herbivory effect^15^,^16^,^17^,^18^,^19^. For example, in a meta-analysis, plants suffered 97% higher herbivory when their mutualistic ants were absent^16^. For plants with both domatia and food bodies, herbivory was found to be up to five times higher when their partner ants were excluded experimentally^17^; among plants that offer ants domatia and honeydew, herbivory increased more than 26 times when the ants were excluded^16^. Even in indirect ant-plant interactions mediated by Hemipterans, plants suffered 55.8% higher herbivory when the ants were absent^15^. Herbivory can be used as “a measure of a plant’s net level of resistance”^20^. Generally speaking, herbivory should be related negatively to the effectiveness of resistance, and the effectiveness of resistance can be expressed as 1 minus the percentage of herbivory^11^,^21^. Recently, through a comparison of biotic, physical, and chemical defenses, a meta-analysis found that the most effective defense strategy for a plant is mutualisms with ants^22^. Thus, if this finding is solid, we should expect a lower level of herbivory for species associated with ants. However, another meta-analysis found that there is a trade-off between direct and indirect defenses at both the intra- and interspecific levels^23^. Therefore, based on this finding, we should expect a similar level of herbivory between species that associate with ants and those that do not. Only a small number of studies have compared the level of herbivory in species that do and do not associate with ants, with mixed conclusions. For example, compared to non-myrmecophytic species, in the genus *Macaranga* the level of herbivory for myremecophytic species was significantly lower^24^,^25^. At the interspecific level in the genus *Inga*, association with ants had no effect on leaf damage; thus, different defensive strategies have equal effectiveness^26^,^27^. Yet another study found that in the genus *Piper*, species that have higher leaf toughness have a significantly lower level of herbivory than do species associated with ants^28^. Therefore, a more generalized comparison of herbivory among a broad range of plant species that use different defensive strategies is needed.

Herbivory and the anti-herbivory effect of ants can be affected by a variety of factors^12^,^29^. First, on a large spatial scale, herbivory is assumed to be higher at lower latitudes because of the high diversity, abundance, and host-plant specialization of herbivores in those areas^30^,^31^, but the generalization of this assumption is still controversial^29^. Both predation by ants and the anti-herbivory effect of ants are considered stronger at lower latitudes^15^,^16^,^17^,^32^. Climate, habitat type, and the life form of the plant species itself also have strong effects on plant herbivory and the anti-herbivory effect of ants^15^,^16^,^17^,^33^,^34^,^35^. In addition, the reward type of plants to ants is another key factor that mediates the anti-herbivory effect of ants on plants; more investments for ants often yield more benefits for plants^15^,^16^,^17^.

Here, through an exploration of published literature and datasets, we evaluated the differences in plant herbivory among ant-associated species and those that have no ant-attractive traits, and identified the possible drivers of the differences. Based on previous models and findings, we hypothesized that: 1) in general, ant-associated species have lower levels of herbivory compared to species that have no ant-attractive traits; 2) the difference in herbivory for the two groups of plants can be context-dependent, and 3) species with higher investments in ants suffer lower levels of herbivory compared to species with lower or no investments in ants.

## Methods

### Literature search

We collated the data on leaf herbivory from a published dataset^36^ and the current literature. Depending upon whether plants have traits that can attract ants, we classified plant species into two groups: species with ants (those with traits that attract ants) and species without ants (those that do not have traits that attract ants). We identified the traits that a species with ants should have: 1) structures such as domatia or other types of specialized hollow spaces that can be used by ants; 2) food bodies; 3) extrafloral nectaries (EFNs), and 4) attractions for honeydew-producing insects attended by ants^15^,^16^,^17^. Species that have one or more of the traits above were classified as species with ants, while others were classified as species without ants.

### Selection criteria and data acquisition

We collected data according to several criteria: 1) herbivory must be measured as the percentage of leaf area consumed by herbivores in order to maintain consistency with the dataset cited^36^; 2) only data that represent plant herbivory across a relatively long period of time were included, excluding data on daily herbivory rates^36^; 3) for experimental studies conducted on the exclusion of ants from plants, both control and treatment data were included; 4) if herbivory was measured in a time series, the mean value of different times was preferred, and 5) only observations conducted in the field were considered; studies conducted in greenhouses or labs were excluded.

We should point out several weaknesses in the methods we used to subdivide species. Some of the limitations were unavoidable, but we believe their effects can be minimized through appropriate adjustments. First, considering the ubiquity of ants in terrestrial ecosystems, we assumed that all plant species with traits that attract ants are tended by ants in nature. Second, it is possible that some plant species with none of the traits above are still patrolled by ants^37^,^38^. The patrolling of ants on plant species with no ant-attractive traits may be accidental and highly variable compared to species that have ant-attractive traits^37^,^39^. Thus, the ecological effects of ants on plant species with no ant-attractive traits should be weaker compared to the effects on those with ant-attractive traits^40^,^41^. Third, in the group we classified as “without ants,” some species can also attract honeydew-producing insects, such as aphids, and thus, they are attractive to ants; these species could be misclassified into the “without ants” group because there have been no reports of their interactions with ants in the literature we found. To address this problem, we argue that if plant species with ants are indeed better protected, species that only offer honeydew for ants should still have lower herbivory compared to the group of species we classified as “without ants”. On the other hand, if species that only offer honeydew for ants suffer higher herbivory compared to those without ants, we can reject the hypothesis that species with ants are better protected. Last, in many cases, plants do not produce rewarding traits, such as EFNs, food bodies and domatia, until they reach a minimum size^42^. The “minimum size” varies greatly across different species; for example, it can range from 0.10 to 2.50 m in height for plants that produce domatia^42^. To address this issue, information about whether the rewarding traits had occurred, and the size or ontogenetic stages of the plants were checked in the original literature.

The world list of angiosperm species with EFNs was used to identify the species that have EFNs in the dataset we cited (http://biosci-labs.unl.edu/Emeriti/keeler/extrafloral/worldlistfamilies.htm)^36^. In addition, we searched related monographs extensively^43^,^44^,^45^,^46^ as well as studies cited in reviews of ant-plant interactions^8^,^9^,^12^,^13^,^14^,^15^,^17^,^18^,^19^,^23^,^47^,^48^,^49^ to identify species with traits that attract ants and data on herbivory.

Furthermore, we used “ant plant,” “ant herbivory,” “ant herbivore,” “ant protection,” “EFNs,” “domatia,” “food body,” “ant-aphid mutualism,” and “ant honeydew” as key words while searching in the ISI Web of Sciences. All of the studies found were checked for the suitability of data collection.

The mean, standard error or deviation, and sample size were extracted from texts, tables, or figures in the literature found. For data expressed in figures, the UTHSCSA Image Tool (University of Texas, USA) was used to obtain the exact values of the data. Information on the latitude and longitude of the study site, climate type, habitat type, and life form of the plant species were collected. Studies that did not report clear latitude and longitude were excluded. The types of rewards offered to ants were classified into four groups: no reward (for species without ants); food only; nesting spaces only, and both food and nesting spaces.

### Data analysis

We preferred to use the unweighted mean herbivory for each data point in data analysis rather than the weighted regression (as in the data analysis of another study^20^), because 59.6% of the data points we used had no clear standard deviation or error, and a weighted regression would exclude these data from analysis. To improve the independence of the data for the same species, prior to data analysis, we averaged the herbivory data for a given species conducted at the same site and in the same climate zone and habitat type and herbivory caused by the same type of herbivores within a reference. The mean value was used in the subsequent data analysis. Thus, under the same biotic and abiotic conditions that we considered, just one data point was included for each species. Because herbivory data are often non-normally distributed, the arithmetic mean gives an upward biased estimate; therefore, we adopted geometric means and 95% confidence intervals (CI) to describe the distribution patterns of the data^20^.

A mixed-effect negative binomial regression model with Laplace approximation was used to evaluate the effects of different factors on herbivory, with species as the random effect. Using this model, we first compared herbivory for species without ants, species associated with ants (control), and species associated with ants, but with the ants excluded experimentally (treatment). In the subsequent analysis, we used only the data for species without ants and the control data for species with ants. Using the model, we evaluated the effects of plant type, life form, climate zone and their interactions on herbivory. The data on polar climate, liana, and ferns were excluded in this analysis due to their small sample sizes. Then we compared herbivory in the two types of plants for several main types of habitats, and plant families. The least squared means were used for multiple comparisons. The model was also used to analyze the effect of reward type on herbivory. A general linear regression was used to evaluate the relationship between herbivory and latitude.

All of the analyses above were conducted using SAS 9.3 (SAS Institute, Inc., Cary, NC, USA). To detect the possible effects of phylogenetic relationships on our analysis, we first compared the herbivory of two groups within the same family; next, we evaluated the relationship between phylogenetic distance and the difference in herbivory for given pairs of species using linear regression. The phylogenetic tree and distance were generated and analyzed using Phylomatics^50^ and the ape package in R 2.15.1 (R Development Core Team 2012)^51^,^52^.

## Results

**Figure 1:**
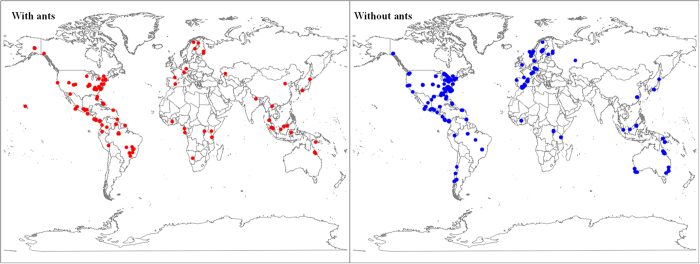
The distribution of study locations, left for species with ants, right for species without ants. The output and data analysis for this paper was generated using SAS software, Version 9.3 of the SAS System for Windows. Copyright © SAS Institute Inc. SAS and all other SAS Institute Inc. Product or service names are registered trademarks or trademarks of SAS Institute Inc., Cary, NC, USA.

**Figure 2:**
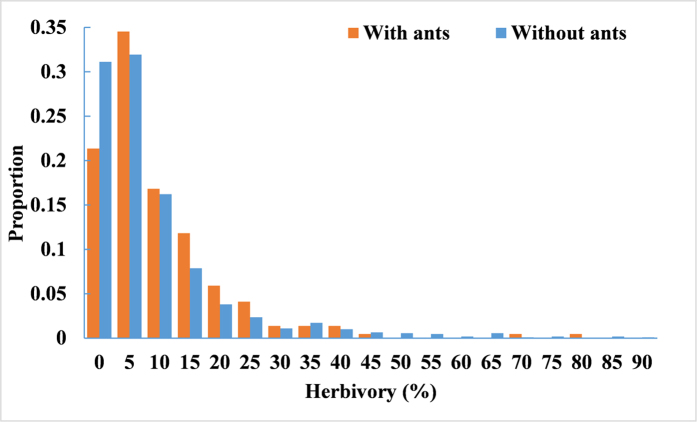
The distribution pattern of herbivory for plant species with and without ants.

From 210 publications, we obtained a final dataset that contained 1,405 data points on plant herbivory for 871 species; 124 species associated with ants and 747 species did not (see Fig. 1 for the global distribution of the study sites and S1 for the dataset). The geometric mean herbivory for all the species was 4.38% (95% CI: 4.07–4.72%, n = 1,325). In most cases, plant herbivory was lower than 10% (Fig. 2). The association with ants had no effect on the distribution pattern of the data (Mantel-Haenszel Chi-Square = 0.14, *P* = 0.7104; Fig. 2).

**Figure 3:**
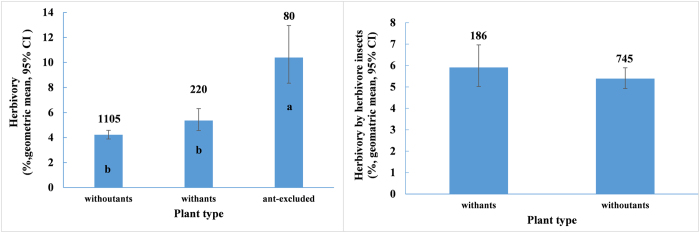
The herbivory for plant species with and without ants. Number on bar represents sample size, different letters on bars mean significant differences (*P* < 0.05).

Species without ants, species with ants, and species with ants but with the ants experimentally excluded had significantly different herbivory (*F*_2, 533_ = 23.48, *P* < 0.0001). The multiple comparisons showed that the first two groups had similar levels of herbivory (t = 1.37, *P* = 0.1710, Fig. 3). When ants were excluded experimentally from ant-associated species, plant herbivory was 93.87% higher (t = 6.11, *P* < 0.0001), and the value was 146.60% higher than herbivory among species without ants (t = 6.19, *P* < 0.0001, Fig. 3). Plant species with ants and those without ants suffered similar levels of herbivory caused by insects (*F*_1, 252_ = 1.41, *P* = 0.2804, Fig. 3).

**Figure 4:**
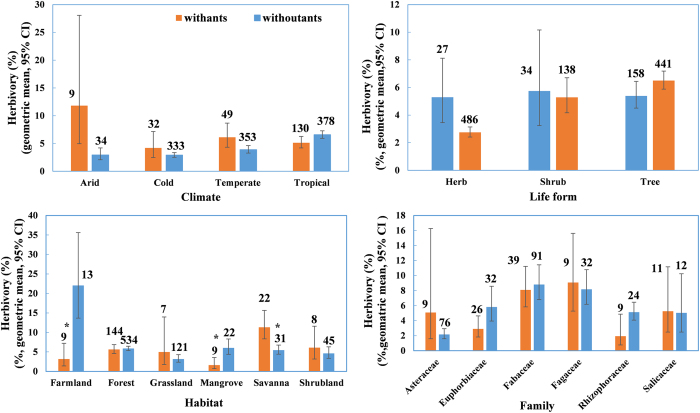
The herbivory for plant species with and without ants in different contexts. Number on bar represent sample size. *represents significant differences (*P* < 0.05).

Both climate and plant life form had significant effects on herbivory (*F*_3, 429_ = 6.67, *P* = 0.0002 and *F*_2, 429_ = 7.51, *P* = 0.0006 respectively), but not on their interaction with plant type (all *P* > 0.1). Further analysis showed that, in cold climates, plants suffered lower herbivory than in tropical, temperate, and arid regions (*P* < 0.05 for all comparisons; Fig. 4), while species in tropical, temperate, and arid regions had similar levels of herbivory (*P* > 0.20 for all comparisons). Herbs had significantly lower levels of herbivory compared to trees (t = 3.46, *P* = 0.0006) and shrubs (t = 3.48, *P* < 0.0004). Herbivory was not significantly different between shrubs and trees (t = 0.59, *P* = 0.5539, Fig. 4).

**Figure 5:**
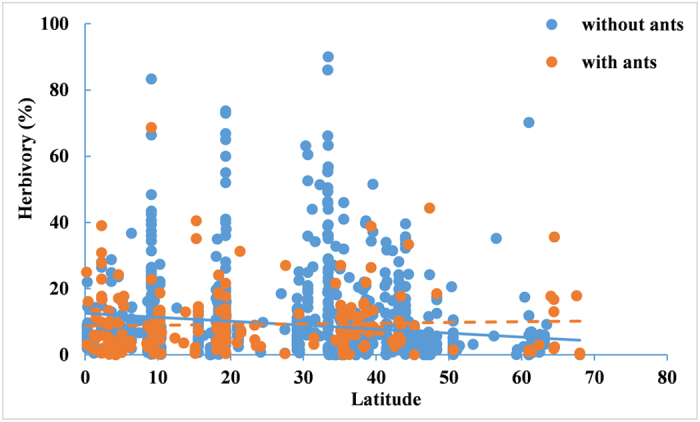
The relationship between herbivory and latitude for plant species with and without ants. Solid line mean significant relationship (*P* < 0.05), dot line means insignificant relationship.

Habitat had no significant effect (*F*_5, 243_ = 1.56, *P* = 0.1341), but their interaction with plant type had a significant effect on herbivory (*F*_5, 243_ = 3.15, *P* = 0.0089). In agricultural areas and mangrove swamps, plants associated with ants suffered significantly lower herbivory (t = 2.43, *P* = 0.0159 and t = 2.07, *P* = 0.0396, Fig. 4). However, in the savanna, species associated with ants suffered significantly higher herbivory compared to those without ants (t = 2.24, *P* = 0.0261). In other habitats, the two groups of plants did not show significant differences in herbivory (Fig. 4). In general, herbivory tended to decrease with latitude (*R*^*2*^ = 1.56%, *P* < 0.0001). However, this pattern changed with the presence of ants: for species with ants, herbivory had no relationship with latitude (*R*^*2*^ = 0.12%, *P* = 0.6230), but for species without ants, herbivory tended to decrease with latitude (*R*^*2*^ = 2.42%, *P* < 0.0001, Fig. 5).

**Figure 6:**
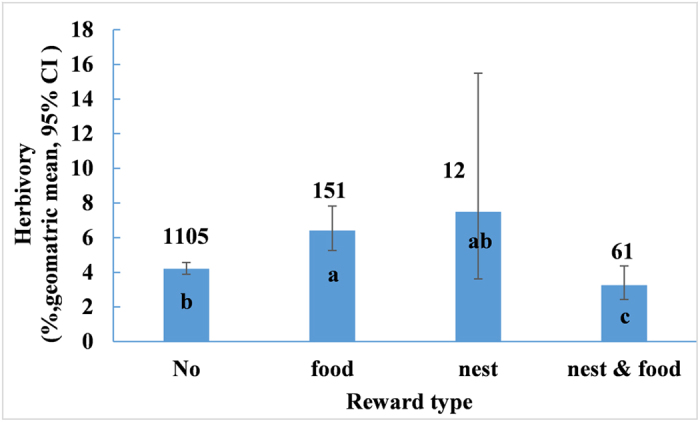
The herbivory of plant species with different types of rewards for ants. Numbers on bar represent sample size; different letters on bars mean significant differences (*P* < 0.05).

Herbivory varied significantly with the type of reward provided for ants (*F*_3, 454_ = 4.2, *P* = 0.0077). Species that offer both food and nesting spaces for ants had the lowest herbivory compared to others (Fig. 6). The herbivory in species that offer either food or nesting spaces was 96.52% and 129.66% higher (t = 3.10, *P* = 0.0021 and t = 2.11, *P* < 0.0354, respectively) than in those that offer both food and nesting spaces for ants. Species that offer ants no reward had a 29.19% higher level of herbivory compared to species that offer ants both food and nesting places (t = 2.08, *P* = 0.0378). Multiple comparisons also showed that species that offer ants food alone had 53.28% higher herbivory compared to species that provide no reward (t = 2.39, *P* = 0.0174). Species that offer ants honeydew alone had a similar level of herbivory (geometric mean = 5.72%, CI = 3.77–8.68, n = 45) as those that offer ants EFNs (geometric mean = 6.70%, CI = 5.34–8.40%, n = 100; *F*_1,60_ = 0.01, *P* = 0.9117). Species that offer ants honeydew alone suffered 35.67% higher herbivory compared to species that provided ants with no reward, but the difference was insignificant (*F*_1, 381_ = 1.22, *P* = 0.2693).

**Figure 7:**
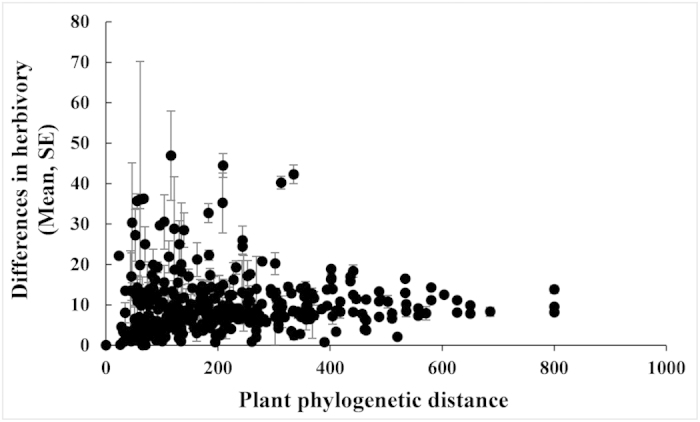
The relationship between the phylogenetic distance and the difference in herbivory for pairs of plant species.

Herbivory varied significantly among different families (*F*_5, 138_ = 4.60, *P* = 0.0006), but, within each family, the two groups of species had similar levels of herbivory (*F*_5, 138_ = 1.76, *P* = 0.1244; Fig. 4). A phylogenetic analysis of 864 of the 871 plant species found that the phylogenetic distances between pairs of species had no relationship to differences in herbivory (t = 0.3796, *P* = 0.7045; Fig. 7). Both results suggest that our findings were unlikely to be affected by the phylogenetic relationship between species. In the 220 data points for plants we classified as “with ants”, 12 (5.45%) of them were collected from saplings with no clear information about whether or not the rewarding traits had occurred on these individuals. Nevertheless, the main results were solid, even when these data points were excluded from the analyses above.

## Discussion

We found that, at the interspecific level, species with different defensive strategies have similar levels of herbivory. However, among those with indirect defenses, species that invested the most in ants indeed had the lowest levels of herbivory. These findings improve our understanding about the relationship between different defensive strategies in plants. The results also highlight the role of obligate mutualistic interactions in shaping the pattern of certain key ecological processes, such as herbivory.

We showed that, at the interspecific level, different defensive strategies can be equally effective. Only with the help of ants, ant associated species can achieve a similar level of herbivory as those that depend primarily on their own direct defenses; when the mutualistic ants were removed experimentally, the level of herbivory for ant associated species was 93.87% higher compared to that for species that do not associate with ants. This pattern indicates that the investment in direct defense should be much lower among species that associate with ants, by comparison to those that do not^23^.With this trade-off, the total effectiveness of different defensive strategies can be equal, which can lead to the pattern that we have shown in this study. In contrast to the findings of a previous study^22^, we found that mutualism with ants is not a more effective defensive strategy than others, and we argue that the effectiveness of direct defense may have been underestimated in previous studies^22^. For plants, ants are “worn on the outside” and it is easy to control their presence or absence experimentally^12^. However, the effect of direct (e.g., chemical) defense is much more difficult to control. In fact, to explore the effect of direct defensive strategies on herbivory, studies often use plants or species that produce different levels of chemical defenses, rather than with the presence or absence of particular direct defense traits. Furthermore, some direct defensive traits function in combination, rather than separately^8^. We agree with the argument that we should “abandon searching for single silver bullet traits” in the study of plant defense^8^, because at the interspecific level, the effectiveness of different defensive strategies may be similar. At the community scale, the structure of ant-plant ecological networks can vary with plant traits, such as the occurrence of EFNs^37^. But a recent study found that EFNs have only a limited effect on the structure of ant communities in the canopy^38^. In addition to these studies, our results suggest that interspecific interaction networks with different structures can have similar ecological effects. The relationship between the structure of ecological networks and certain key ecological processes, such as herbivory, should be highlighted in future studies.

We confirmed that species with higher investments in ants indeed suffered lower levels of herbivory compared to those that invested less. Producing specialized structures such as food bodies and domatia are costly to plants, and higher investments are predicted to be associated with higher rewards in ant-plant interactions^14^,^53^. This prediction has been confirmed in some case studies^54^,^55^ and meta-analyses^16^,^17^,^18^. However, at the interspecific level, whether species with higher investments in ants really have lower levels of herbivory has been poorly studied. The positive feedback between investment and rewards can lead to the persistence of the highly specialized mutualistic interactions between ants and plants over evolutionary time. The ants associated with plants that offer them greater rewards are often more aggressive, which can lead to stronger anti-herbivory effects^56^,^57^. It must be pointed out that the investment of plants in indirect defenses often varies across their ontogenetic stages^42^. Plants do not produce rewarding traits until they reach a minimum size, which varies greatly across species^42^. In our dataset, 12 (5.45%) of the data points for ant-associated plants were collected from saplings for which there was no clear information about whether or not these individuals had reached the “minimum size” necessary for the production of rewarding traits. Therefore, we could not evaluate the relationship between herbivory and the shifts in defensive strategies for ant-associated species. Two recent studies highlighted the importance of seasonal variation in different defensive strategies for plants with EFNs^58^,^59^. But because the lack of related information in the used dataset, we haven’t evaluated this effect in our study. We suggest the ontogenetic and seasonal shifts of plant defensive strategies and the corresponding effects on herbivory should be addressed in future studies.

We noticed that 59 of the 62 studies on obligate ant-plant interactions occurred in tropical regions, which have higher plant and herbivore diversity and stronger biotic interactions^60^,^61^. Therefore, in tropical regions, the obligate ants may play an important role in helping their host plants cope with specialized herbivores or catastrophic damage^62^. In some habitats, such as agricultural areas and mangrove swamps, plant species associated with ants have significantly lower herbivory compared to those without ants. Both agricultural and mangrove habitats are lower in biodiversity and habitat complexity, which may make ant-herbivore conflict unavoidable and yield a stronger anti-herbivory effect. In the savanna, species without ants suffered significantly higher herbivory compared to those with ants, and the mechanism underlying this pattern is unclear. We suggest that in the savanna, the relationship between herbivory and the association with ants at the interspecific level should be focused on in future studies, because the classic example of mutualism, the ant-*Acacia* interaction, occurs in this area.

This study found a negative relationship between herbivory and latitude, but the relationship could be changed by ants. Both herbivore pressure and the anti-herbivory effect of ants are stronger at lower latitudes^15^,^16^,^17^,^32^,^63^. For ant-associated species, higher herbivory pressure in tropical areas can be relieved by the stronger anti-herbivory effect of ants, which may cause the relationship between herbivory and latitude to become insignificant, as we showed here. It should be noted that latitude explains only a very small portion of the variation in herbivory (1.56% for all species and 2.42% for species that are not associated with ants). More variables and mechanisms should be included in future models to explain the variation in herbivory across large spatial scales.

In general, we confirmed the equal effectiveness of different defensive strategies at the interspecific level, but highly specialized ant-plant interactions are indeed a more effective defensive trait than are others. Considering that herbivory has profound effects on plant performance and fitness^64^,^65^, suffering equal levels of herbivory may play an important role in the coexistence of species that use different defensive strategies over evolutionary time.

## Additional Information

**How to cite this article**: Zhang, S. *et al.* The equal effectiveness of different defensive strategies. *Sci. Rep.* **5**, 13049; doi: 10.1038/srep13049 (2015).

## Supplementary Material

Supplementary Information
