# Supplementary material for: The equal effectiveness of different defensive strategies
Source: Sci Rep. 2015 Aug 12;5:13049. doi: 10.1038/srep13049 (PMC4533318; doi:10.1038/srep13049)
Supplement: Supplementary Information [file srep13049-s1.pdf]

Supplementary information for "The equal effectiveness of different defensive strategies"

Shuang Zhang, Yuxin Zhang, and Keming Ma*

Table S1, The dataset used in the study.

The data for studies from 1 to 1221 were based on the data set of Turcotte et al. (2014).

For abbreviation:

PT, plant type, WOA, without ants, WA, with ants, AE, ants excluded;

Lat, latitude, Lon, longitude;

Cl, climate, T, temperate, Tro, tropical, a, Aric, C, cold, P, Polar;

LF, life form, T tree, H, herb, S, shrub, F, fern, L, liana, V, Vine;

Reward, F, food, N, nest, F & N, food & nest;

H, herbivory(%);

R, references

ID	Species	PT	Lat	Lon	Cl	LF	Habitat	Reward	Family	Consumers	H	R
1	Aextoxicon punctatum	WOA	41.6S	73.9W	T	T	Forest	NO	Aextoxicaceae	Insect	8.90	43
2	Nothofagus pumilio	WOA	40.57S	71.56W	T	T	Forest	NO	Nothofagaceae	Insect	6.49	69
3	Nothofagus pumilio	WOA	40.37S	71.5W	T	T	Forest	NO	Nothofagaceae	Insect	6.10	70
4	Nothofagus pumilio	WOA	40.36S	71.05W	T	T	Forest	NO	Nothofagaceae	Insect	14.30	70
5	Aristotelia chilensis	WOA	35.58S	72.42W	T	T	Forest	NO	Elaeocarpaceae	Insect	9.01	197
6	Eucalyptus delegatensis	WOA	35.17S	148.44E	T	T	Forest	NO	Myrtaceae	Insect	4.03	148
7	Eucalyptus dives	WOA	35.17S	148.44E	T	T	Forest	NO	Myrtaceae	Insect	5.34	148
8	Eucalyptus pauciflora	WOA	35.17S	148.44E	T	T	Forest	NO	Myrtaceae	Insect	9.92	148
9	Eucalyptus globulus	WOA	34.53S	118.05E	T	T	Forest	NO	Myrtaceae	Insect	18.10	112

10	Eucalyptus globulus	WOA	34.44S	117.2E	T	T	Forest	NO	Myrtaceae	Insect	32.80	112
11	Eucalyptus globulus	WOA	34.33S	117.02E	T	T	Forest	NO	Myrtaceae	Insect	20.90	112
12	Eucalyptus marginata	WOA	34.14S	116.8E	T	T	Forest	NO	Myrtaceae	Insect	12.47	1
13	Eucalyptus globulus	WOA	34.14S	116.11E	T	T	Forest	NO	Myrtaceae	Insect	27.10	112
14	Acacia terminalis	WOA	33.39S	151.14E	T	S	Terrace	NO	Fabaceae	Insect	29.06	131
15	Banksia oblongifolia	WOA	33.39S	151.14E	T	S	Terrace	NO	Proteaceae	Insect	1.50	131
16	Banksia serrata	WOA	33.39S	151.14E	T	S	Terrace	NO	Proteaceae	Insect	2.16	131
17	Callicoma serratifolia	WOA	33.39S	151.14E	T	S	Terrace	NO	Cunoniaceae	Insect	19.62	131
18	Ceratopetalum gummiferum	WOA	33.39S	151.14E	T	T	Terrace	NO	Cunoniaceae	Insect	24.90	131
19	Grevillea buxifolia	WOA	33.39S	151.14E	T	S	Terrace	NO	Proteaceae	Insect	5.67	131
20	Grevillea sericea	WOA	33.39S	151.14E	T	S	Terrace	NO	Proteaceae	Insect	7.74	131
21	Hakea dactyloides	WOA	33.39S	151.14E	T	S	Terrace	NO	Proteaceae	Insect	22.20	131
22	Hakea gibbosa	WOA	33.39S	151.14E	T	S	Terrace	NO	Proteaceae	Insect	13.67	131
23	Pimelea linifolia	WOA	33.39S	151.14E	T	S	Terrace	NO	Thymelaeaceae	Insect	2.43	131
24	Pomaderris lanigera	WOA	33.39S	151.14E	T	S	Terrace	NO	Rhamnaceae	Insect	12.00	131
25	Pultenaea flexillis	WOA	33.39S	151.14E	T	S	Terrace	NO	Fabaceae	Insect	9.86	131
26	Xanthosia pilosa	WOA	33.39S	151.14E	T	S	Terrace	NO	Apiaceae	Insect	1.19	131
27	Angophora hispida	WOA	33.39S	151.13E	T	T	Ridge	NO	Myrtaceae	Insect	90.02	131
28	Banksia ericifolia	WOA	33.39S	151.13E	T	S	Ridge	NO	Proteaceae	Insect	16.00	131
29	Banksia serrata	WOA	33.39S	151.13E	T	S	Ridge	NO	Proteaceae	Insect	1.47	131
30	Darwinia fascicularis	WOA	33.39S	151.13E	T	S	Ridge	NO	Myrtaceae	Insect	36.11	131
31	Epacris tubiflora	WOA	33.39S	151.13E	T	S	Ridge	NO	Ericaceae	Insect	63.30	131
32	Eucalyptus haemastoma	WOA	33.39S	151.13E	T	T	Ridge	NO	Myrtaceae	Insect	56.80	131
33	Grevillea sericea	WOA	33.39S	151.13E	T	S	Ridge	NO	Proteaceae	Insect	35.10	131
34	Grevillea speciosa	WOA	33.39S	151.13E	T	S	Ridge	NO	Proteaceae	Insect	1.06	131
35	Hakea teretifolia	WOA	33.39S	151.13E	T	S	Ridge	NO	Proteaceae	Insect	39.30	131
36	Isopogon anethifolius	WOA	33.39S	151.13E	T	S	Ridge	NO	Proteaceae	Insect	28.54	131
37	Leucopogon esquamatus	WOA	33.39S	151.13E	T	S	Ridge	NO	Ericaceae	Insect	44.00	131
38	Leucopogon microphyllus	WOA	33.39S	151.13E	T	S	Ridge	NO	Ericaceae	Insect	8.57	131
39	Pultenaea elliptica	WOA	33.39S	151.13E	T	S	Ridge	NO	Fabaceae	Insect	15.60	131
40	Tetratheca ericifolia	WOA	33.39S	151.13E	T	S	Ridge	NO	Elaeocarpaceae	Insect	23.14	131
41	Acacia floribunda	WOA	33.33S	151.17E	T	S	Volcanic soil	NO	Fabaceae	Insect	55.47	131

42	Eucalyptus umbra	WOA	33.33S	151.17E	T	T	Volcanic soil	NO	Myrtaceae	Insect	46.31	131
43	Glycine tabacina	WOA	33.33S	151.17E	T	H	Volcanic soil	NO	Fabaceae	Insect	66.15	131
44	Hardenbergia violacea	WOA	33.33S	151.17E	T	V	Volcanic soil	NO	Fabaceae	Insect	86.05	131
45	Lasiopetalum ferrugineum	WOA	33.33S	151.17E	T	S	Volcanic soil	NO	Malvaceae	Insect	50.50	131
46	Pandorea pandorana	WOA	33.33S	151.17E	T	V	Volcanic soil	NO	Bignoniaceae	Insect	55.32	131
47	Pomaderris ferruginea	WOA	33.33S	151.17E	T	S	Volcanic soil	NO	Rhamnaceae	Insect	45.78	131
48	Prostanthera denticulata	WOA	33.33S	151.17E	T	S	Volcanic soil	NO	Lamiaceae	Insect	49.30	131
49	Pultenaea daphnoides	WOA	33.33S	151.17E	T	S	Volcanic soil	NO	Fabaceae	Insect	11.84	131
50	Pultenaea flexillis	WOA	33.33S	151.17E	T	S	Volcanic soil	NO	Fabaceae	Insect	38.97	131
51	Syncarpia glomulifera	WOA	33.33S	151.17E	T	T	Volcanic soil	NO	Myrtaceae	Insect	36.30	131
52	Xylomelum pyriforme	WOA	33.33S	151.17E	T	T	Volcanic soil	NO	Proteaceae	Insect	18.80	131
53	Eucalyptus botryoides	WOA	31.57S	115.5E	T	T	Forest	NO	Myrtaceae	Insect	10.80	159
54	Eucalyptus gomphocephala	WOA	31.57S	115.5E	T	T	Forest	NO	Myrtaceae	Insect	6.70	159
55	Eucalyptus marginata	WOA	31.57S	115.5E	T	T	Forest	NO	Myrtaceae	Insect	10.30	159
56	Angophora floribunda	WOA	30.59S	151.29E	T	T	Grassland	NO	Myrtaceae	Insect	17.00	116
57	Eucalyptus blakelyi	WOA	30.59S	151.29E	T	T	Forest	NO	Myrtaceae	Insect	7.50	116
58	Eucalyptus blakelyi	WOA	30.59S	151.29E	T	T	Grassland	NO	Myrtaceae	Insect	16.00	116
59	Eucalyptus caliginosa	WOA	30.59S	151.29E	T	T	Forest	NO	Myrtaceae	Insect	24.90	116
60	Eucalyptus caliginosa	WOA	30.59S	151.29E	T	T	Grassland	NO	Myrtaceae	Insect	20.90	116
61	Eucalyptus dalrympleana	WOA	30.59S	151.29E	T	T	Grassland	NO	Myrtaceae	Insect	19.00	116
62	Eucalyptus melliodora	WOA	30.59S	151.29E	T	T	Forest	NO	Myrtaceae	Insect	10.20	116
63	Eucalyptus nova-anglica	WOA	30.59S	151.29E	T	T	Grassland	NO	Myrtaceae	Insect	60.50	116

64	Eucalyptus radiata	WOA	30.59S	151.29E	T	T	Forest	NO	Myrtaceae	Insect	14.30	116
65	Eucalyptus stellulata	WOA	30.59S	151.29E	T	T	Grassland	NO	Myrtaceae	Insect	35.90	116
66	Eucalyptus viminalis	WOA	30.59S	151.29E	T	T	Forest	NO	Myrtaceae	Insect	19.80	116
67	Eucalyptus viminalis	WOA	30.59S	151.29E	T	T	Grassland	NO	Myrtaceae	Insect	52.60	116
68	Eucalyptus youmanii	WOA	30.59S	151.29E	T	T	Forest	NO	Myrtaceae	Insect	14.30	116
69	Aextoxicon punctatum	WOA	30.4S	71.3W	A	T	Forest	NO	Aextoxicaceae	Insect	6.00	43
70	Acanthus ilicifolius	WOA	19.17S	147.03E	Tro	S	Forest	NO	Acanthaceae	Insect	9.00	165
71	Acrostichum speciosum	WOA	19.17S	147.03E	Tro	H	Forest	NO	Pteridaceae	Insect	3.90	165
72	Aegialitis annulata	WOA	19.17S	147.03E	Tro	T	Forest	NO	Plumbaginaceae	Insect	1.20	165
73	Aegiceras corniculatum	WOA	19.17S	147.03E	Tro	T	Forest	NO	Primulaceae	Insect	10.50	165
74	Avicennia marina	WOA	19.17S	147.03E	Tro	T	Forest	NO	Acanthaceae	Insect	12.00	165
75	Bruguiera exaristata	WOA	19.17S	147.03E	Tro	T	Forest	NO	Rhizophoraceae	Insect	2.90	165
76	Bruguiera gymnorhiza	WOA	19.17S	147.03E	Tro	T	Forest	NO	Rhizophoraceae	Insect	1.80	165
77	Ceriops australis	WOA	19.17S	147.03E	Tro	T	Forest	NO	Rhizophoraceae	Insect	6.80	165
78	Excoecaria agallocha	WOA	19.17S	147.03E	Tro	T	Forest	NO	Euphorbiaceae	Insect	1.50	165
79	Lumnitzera racemosa	WOA	19.17S	147.03E	Tro	T	Forest	NO	Combretaceae	Insect	3.00	165
80	Osbornia octodonta	WOA	19.17S	147.03E	Tro	T	Forest	NO	Myrtaceae	Insect	6.20	165
81	Sonneratia alba	WOA	19.17S	147.03E	Tro	T	Forest	NO	Lythraceae	Insect	1.20	165
82	Xylocarpus australasicus	WOA	19.17S	146E	Tro	T	Forest	NO	Meliaceae	Insect	3.50	165
83	Acrostichum speciosum	WOA	18.15S	146.12E	Tro	H	Forest	NO	Pteridaceae	Insect	3.10	165
84	Aegiceras corniculatum	WOA	18.15S	146.12E	Tro	T	Forest	NO	Primulaceae	Insect	16.00	165
85	Avicennia marina	WOA	18.15S	146.12E	Tro	T	Forest	NO	Acanthaceae	Insect	8.80	165
86	Bruguiera gymnorhiza	WOA	18.15S	146.12E	Tro	T	Forest	NO	Rhizophoraceae	Insect	3.70	165
87	Ceriops australis	WOA	18.15S	146.12E	Tro	T	Forest	NO	Rhizophoraceae	Insect	6.30	165
88	Excoecaria agallocha	WOA	18.15S	146.12E	Tro	T	Forest	NO	Euphorbiaceae	Insect	0.30	165
89	Heritiera littoralis	WOA	18.15S	146.12E	Tro	T	Forest	NO	Malvaceae	Insect	35.00	165
90	Rhizophora apiculata	WOA	18.15S	146.12E	Tro	T	Forest	NO	Rhizophoraceae	Insect	5.80	165
91	Rhizophora x lamarckii	WOA	18.15S	146.12E	Tro	T	Forest	NO	Rhizophoraceae	Insect	1.40	165
92	Scyphiphora hydrophyllacea	WOA	18.15S	146.12E	Tro	S	Forest	NO	Rubiaceae	Insect	0.70	165
93	Xylocarpus australasicus	WOA	18.15S	146E	Tro	T	Forest	NO	Meliaceae	Insect	3.00	165
94	Xylocarpus granatum	WOA	18.15S	146E	Tro	T	Forest	NO	Meliaceae	Insect	10.00	165
95	Acanthus ilicifolius	WOA	18.01S	146.02E	Tro	S	Forest	NO	Acanthaceae	Insect	6.80	165
96	Aegiceras corniculatum	WOA	18.01S	146.02E	Tro	T	Forest	NO	Primulaceae	Insect	17.10	165

97	Avicennia marina	WOA	18.01S	146.02E	Tro	T	Forest	NO	Acanthaceae	Insect	10.50	165
98	Bruguiera gymnorhiza	WOA	18.01S	146.02E	Tro	T	Forest	NO	Rhizophoraceae	Insect	2.80	165
99	Bruguiera parviflora	WOA	18.01S	146.02E	Tro	T	Forest	NO	Rhizophoraceae	Insect	3.30	165
100	Cynometra iripa	WOA	18.01S	146.02E	Tro	T	Forest	NO	Fabaceae	Insect	19.70	165
101	Excoecaria agallocha	WOA	18.01S	146.02E	Tro	T	Forest	NO	Euphorbiaceae	Insect	0.70	165
102	Heritiera littoralis	WOA	18.01S	146.02E	Tro	T	Forest	NO	Malvaceae	Insect	29.70	165
103	Lumnitzera racemosa	WOA	18.01S	146.02E	Tro	T	Forest	NO	Combretaceae	Insect	4.60	165
104	Rhizophora apiculata	WOA	18.01S	146.02E	Tro	T	Forest	NO	Rhizophoraceae	Insect	3.90	165
105	Sonneratia alba	WOA	18.01S	146.02E	Tro	T	Forest	NO	Lythraceae	Insect	10.70	165
106	Sonneratia caseolaris	WOA	18.01S	146.02E	Tro	T	Forest	NO	Lythraceae	Insect	9.90	165
107	Sonneratia x gulngai	WOA	18.01S	146.02E	Tro	T	Forest	NO	Lythraceae	Insect	7.80	165
108	Xylocarpus granatum	WOA	18.01S	146E	Tro	T	Forest	NO	Meliaceae	Insect	6.90	165
109	Calamus australis	WOA	17.23S	145.43E	T	V	Forest	NO	Arecaceae	Insect	1.20	77
110	Cordyline cannifolia	WOA	17.23S	145.43E	T	S	Forest	NO	Asparagaceae	Insect	1.50	77
111	Freycinetia excelsa	WOA	17.23S	145.43E	T	V	Forest	NO	Pandanaceae	Insect	0.00	77
112	Pothos longipes	WOA	17.23S	145.43E	T	V	Forest	NO	Araceae	Insect	6.60	77
113	Alocasia brisbanensis	WOA	17.18S	145.26E	T	H	Forest	NO	Araceae	Insect	2.00	77
114	Calamus caryotoides	WOA	17.18S	145.26E	T	V	Forest	NO	Arecaceae	Insect	2.20	77
115	Pothos longipes	WOA	17.18S	145.26E	T	V	Forest	NO	Araceae	Insect	0.00	77
116	Aspidosperma tomentosum	WOA	15.56S	44.55W	Tro	T	Savanna	NO	Apocynaceae	Insect	8.86	122
117	Barjonia erecta	WOA	15.56S	44.55W	Tro	H	Savanna	NO	Apocynaceae	Insect	0.65	122
118	Borreria spp.	WOA	15.56S	44.55W	Tro	H	Savanna	NO	Rubiaceae	Insect	6.99	122
119	Byrsonima coccolobifolia	WOA	15.56S	44.55W	Tro	T	Savanna	NO	Malpighiaceae	Insect	6.91	122
120	Byrsonima crassa	WOA	15.56S	44.55W	Tro	T	Savanna	NO	Malpighiaceae	Insect	9.11	122
121	Byrsonima verbascifolia	WOA	15.56S	44.55W	Tro	T	Savanna	NO	Malpighiaceae	Insect	3.74	122
122	Chomelia ribesioides	WOA	15.56S	44.55W	Tro	S	Savanna	NO	Rubiaceae	Insect	9.43	122
123	Croton goyazensis	WOA	15.56S	44.55W	Tro	T	Savanna	NO	Euphorbiaceae	Insect	7.80	122
124	Dalechampia caperonioides	WOA	15.56S	44.55W	Tro	H	Savanna	NO	Euphorbiaceae	Insect	2.68	122
125	Erythroxylum campestre	WOA	15.56S	44.55W	Tro	S	Savanna	NO	Rhizophoraceae	Insect	4.47	122
126	Erythroxylum deciduum	WOA	15.56S	44.55W	Tro	S	Savanna	NO	Rhizophoraceae	Insect	6.91	122
127	Erythroxylum suberosum	WOA	15.56S	44.55W	Tro	T	Savanna	NO	Rhizophoraceae	Insect	13.25	122
128	Erythroxylum tortuosum	WOA	15.56S	44.55W	Tro	T	Savanna	NO	Rhizophoraceae	Insect	2.93	122
129	Hyptis saxatilis	WOA	15.56S	44.55W	Tro	S	Savanna	NO	Lamiaceae	Insect	5.12	122

130	Kielmeyera coriacea	WOA	15.56S	44.55W	Tro	T	Savanna	NO	Clusiaceae	Insect	4.63	122
131	Kielmeyera variabilis	WOA	15.56S	44.55W	Tro	S	Savanna	NO	Clusiaceae	Insect	4.63	122
132	Manihot spp.	WOA	15.56S	44.55W	Tro	T	Savanna	NO	Euphorbiaceae	Insect	5.53	122
133	Maprounea guianensis	WOA	15.56S	44.55W	Tro	S	Savanna	NO	Euphorbiaceae	Insect	3.98	122
134	Palicourea rigida	WOA	15.56S	44.55W	Tro	T	Savanna	NO	Rubiaceae	Insect	5.04	122
135	Pavonia rosa-campestris	WOA	15.56S	44.55W	Tro	S	Savanna	NO	Malvaceae	Insect	10.24	122
136	Peixotoa goiana	WOA	15.56S	44.55W	Tro	S	Savanna	NO	Malpighiaceae	Insect	5.04	122
137	Sabicea brasiliensis	WOA	15.56S	44.55W	Tro	S	Savanna	NO	Rubiaceae	Insect	12.03	122
138	Aegialitis annulata	WOA	9.285S	147.11E	Tro	T	Mangrove	NO	Plumbaginaceae	Insect	9.20	95
139	Aegiceras corniculatum	WOA	9.285S	147.11E	Tro	T	Mangrove	NO	Primulaceae	Insect	4.40	95
140	Avicennia marina	WOA	9.285S	147.11E	Tro	T	Mangrove	NO	Acanthaceae	Insect	5.90	95
141	Avicennia officinalis	WOA	9.285S	147.11E	Tro	T	Mangrove	NO	Acanthaceae	Insect	6.90	95
142	Avicennia rumphiana	WOA	9.285S	147.11E	Tro	T	Mangrove	NO	Acanthaceae	Insect	5.90	95
143	Bruguiera cylindrica	WOA	9.285S	147.11E	Tro	T	Mangrove	NO	Rhizophoraceae	Insect	6.60	95
144	Bruguiera exaristata	WOA	9.285S	147.11E	Tro	T	Mangrove	NO	Rhizophoraceae	Insect	7.50	95
145	Bruguiera gymnorhiza	WOA	9.285S	147.11E	Tro	T	Mangrove	NO	Rhizophoraceae	Insect	4.92	95
146	Ceriops australis	WOA	9.285S	147.11E	Tro	T	Mangrove	NO	Rhizophoraceae	Insect	9.60	95
147	Ceriops decandra	WOA	9.285S	147.11E	Tro	T	Mangrove	NO	Rhizophoraceae	Insect	6.40	95
148	Ceriops tagal var. tagals	WOA	9.285S	147.11E	Tro	T	Mangrove	NO	Rhizophoraceae	Insect	8.60	95
149	Excoecaria agallocha	WOA	9.285S	147.11E	Tro	T	Mangrove	NO	Euphorbiaceae	Insect	1.30	95
150	Heritiera littoralis	WOA	9.285S	147.11E	Tro	T	Mangrove	NO	Malvaceae	Insect	14.20	95
151	Lumnitzera racemosa	WOA	9.285S	147.11E	Tro	T	Mangrove	NO	Combretaceae	Insect	9.20	95
152	Osbornia octodonta	WOA	9.285S	147.11E	Tro	T	Mangrove	NO	Myrtaceae	Insect	5.90	95
153	Rhizophora apiculata	WOA	9.285S	147.11E	Tro	T	Mangrove	NO	Rhizophoraceae	Insect	8.60	95
154	Rhizophora lamarckii	WOA	9.285S	147.11E	Tro	T	Mangrove	NO	Rhizophoraceae	Insect	5.40	95
155	Sonneratia alba	WOA	9.285S	147.11E	Tro	T	Mangrove	NO	Lythraceae	Insect	7.80	95
156	Xylocarpus australasicus	WOA	9.28S	147E	Tro	T	Mangrove	NO	Meliaceae	Insect	9.00	95
157	Xylocarpus mekongensis	WOA	9.28S	147E	Tro	T	Mangrove	NO	Meliaceae	Insect	9.70	95
158	Swietenia macrophylla	WOA	7.46S	51.5W	Tro	T	Forest	NO	Meliaceae	Insect	10.34	143
159	Swietenia macrophylla	WOA	7.46S	51.5W	Tro	T	Forest	NO	Meliaceae	Insect	6.64	144
160	Aglaia mackiana	WOA	6.43S	145E	Tro	T	Forest	NO	Meliaceae	NA	14.50	118
161	Actinodaphne spp.	WOA	6.351S	145.4E	Tro	T	Forest	NO	Lauraceae	Insect	10.60	48
162	Ardisia spp.	WOA	6.351S	145.4E	Tro	T	Forest	NO	Primulaceae	Insect	1.90	48

163	Caldcluvia nymanii	WOA	6.351S	145.4E	Tro	H	Forest	NO	Cunoniaceae	Insect	7.00	48
164	Elaeocarpus ptilanthus	WOA	6.351S	145.4E	Tro	T	Forest	NO	Elaeocarpaceae	Insect	4.80	48
165	Elaeocarpus sayeri	WOA	6.351S	145.4E	Tro	T	Forest	NO	Elaeocarpaceae	Insect	9.70	48
166	Phyllanthus choristylus	WOA	6.351S	145.4E	Tro	H	Forest	NO	Phyllanthaceae	Insect	6.00	48
167	Pouteria firma	WOA	6.351S	145.4E	Tro	T	Forest	NO	Sapotaceae	Insect	4.10	48
168	Prunus pullei	WOA	6.351S	145.4E	Tro	T	Forest	NO	Rosaceae	Insect	7.60	48
169	Schizomeria spp.	WOA	6.351S	145.4E	Tro	T	Forest	NO	Cunoniaceae	Insect	36.70	48
170	Sphenostemon papuanum	WOA	6.351S	145.4E	Tro	T	Forest	NO	Paracryphiaceae	Insect	14.20	48
171	Syzygium spp.	WOA	6.351S	145.4E	Tro	T	Forest	NO	Myrtaceae	Insect	10.85	48
172	Artocarpus heterophyllus	WOA	5.053S	38.38E	Tro	T	Forest	NO	Moraceae	NA	5.99	39
173	Camellia sinensis	WOA	5.053S	38.38E	Tro	S	Forest	NO	Theaceae	NA	4.25	39
174	Castilla elastica	WOA	5.053S	38.38E	Tro	T	Forest	NO	Moraceae	NA	4.40	39
175	Cinchona pubescens	WOA	5.053S	38.38E	Tro	T	Forest	NO	Rubiaceae	NA	2.16	39
176	Cinnamomum camphora	WOA	5.053S	38.38E	Tro	T	Forest	NO	Lauraceae	NA	6.92	39
177	Cinnamomum verum	WOA	5.053S	38.38E	Tro	T	Forest	NO	Lauraceae	NA	8.25	39
178	Clidemia hirta	WOA	5.053S	38.38E	Tro	S	Forest	NO	Melastomataceae	NA	4.68	39
179	Coffea canephora	WOA	5.053S	38.38E	Tro	T	Forest	NO	Rubiaceae	NA	5.57	39
180	Cola nitida	WOA	5.053S	38.38E	Tro	T	Forest	NO	Malvaceae	NA	8.10	39
181	Eugenia spp.	WOA	5.053S	38.38E	Tro	T	Forest	NO	Myrtaceae	NA	12.12	39
182	Hydnocarpus pentandra	WOA	5.053S	38.38E	Tro	T	Forest	NO	Achariaceae	NA	6.16	39
183	Ilex paraguariensis	WOA	5.053S	38.38E	Tro	T	Forest	NO	Aquifoliaceae	NA	3.74	39
184	Maesopsis eminii	WOA	5.053S	38.38E	Tro	T	Forest	NO	Rhamnaceae	NA	1.16	39
185	Manihot glaziovii	WOA	5.053S	38.38E	Tro	T	Forest	NO	Euphorbiaceae	NA	4.08	39
186	Myroxylon peruiferum	WOA	5.053S	38.38E	Tro	T	Forest	NO	Fabaceae	NA	11.33	39
187	Pimenta racemosa	WOA	5.053S	38.38E	Tro	T	Forest	NO	Myrtaceae	NA	0.93	39
188	Piper aduncum	WOA	5.053S	38.38E	Tro	T	Forest	NO	Piperaceae	NA	4.63	39
189	Psidium cattleianum	WOA	5.053S	38.38E	Tro	T	Forest	NO	Myrtaceae	NA	5.57	39
190	Psidium guajava	WOA	5.053S	38.38E	Tro	T	Forest	NO	Myrtaceae	NA	3.23	39
191	Senna multijuga	WOA	5.053S	38.38E	Tro	T	Forest	NO	Fabaceae	NA	6.50	39
192	Syzygium jambos	WOA	5.053S	38.38E	Tro	T	Forest	NO	Myrtaceae	NA	3.31	39
193	Syzygium malaccense	WOA	5.053S	38.38E	Tro	T	Forest	NO	Myrtaceae	NA	5.28	39
194	Acer oblongum	WOA	5.05S	38.3E	Tro	T	Forest	NO	Sapindaceae	NA	4.52	39
195	Toona ciliata	WOA	5.05S	38.3E	Tro	T	Forest	NO	Meliaceae	NA	0.84	39

196	Eriotheca sp.	WOA	3.57S	73.24W	Tro	T	Forest	NO	Malvaceae	Insect	28.74	58
197	Hevea guianensis	WOA	3.57S	73.24W	Tro	T	Forest	NO	Euphorbiaceae	Insect	9.10	58
198	Hevea parviflora	WOA	3.57S	73.24W	Tro	T	Forest	NO	Euphorbiaceae	Insect	7.87	58
199	Mabea sessiliflorum	WOA	3.57S	73.24W	Tro	T	Forest	NO	Euphorbiaceae	Insect	12.94	58
200	Mabea spp.	WOA	3.57S	73.24W	Tro	T	Forest	NO	Euphorbiaceae	Insect	6.18	58
201	Macrolobium angustifolium	WOA	3.57S	73.24W	Tro	T	Forest	NO	Fabaceae	Insect	24.86	58
202	Macrolobium limbatum	WOA	3.57S	73.24W	Tro	T	Forest	NO	Fabaceae	Insect	10.68	58
203	Macrolobium spp.	WOA	3.57S	73.24W	Tro	T	Forest	NO	Fabaceae	Insect	2.63	58
204	Pachira brevipes	WOA	3.57S	73.24W	Tro	T	Forest	NO	Malvaceae	Insect	22.17	58
205	Chrysophyllum pomiferum	WOA	2.25S	59.5W	Tro	T	Forest	NO	Sapotaceae	NA	26.48	18
206	Henriettella caudata	WOA	2.25S	59.5W	Tro	T	Forest	NO	Melastomataceae	Insect	7.98	53
207	Micropholis venulosa	WOA	2.25S	59.5W	Tro	T	Forest	NO	Sapotaceae	NA	14.82	18
208	Pouteria caimito	WOA	2.25S	59.5W	Tro	T	Forest	NO	Sapotaceae	NA	28.01	18
209	Pouteria peruviensis	WOA	2.25S	59.5W	Tro	T	Forest	NO	Sapotaceae	NA	7.86	18
210	Celtis africana	WOA	0.27N	30.25E	Tro	T	Forest	NO	Cannabaceae	Insect	4.00	28
211	Celtis durandii	WOA	0.27N	30.25E	Tro	T	Forest	NO	Cannabaceae	Insect	2.00	28
212	Chaetachme aristata	WOA	0.27N	30.25E	Tro	T	Forest	NO	Ulmaceae	Insect	4.00	28
213	Chrysophyllum spp.	WOA	0.27N	30.25E	Tro	T	Forest	NO	Sapotaceae	Insect	8.00	28
214	Erythrina abyssinica	WOA	0.27N	30.25E	Tro	T	Forest	NO	Fabaceae	Insect	2.00	28
215	Funtumia latifolia	WOA	0.27N	30.25E	Tro	T	Forest	NO	Apocynaceae	Insect	9.00	28
216	Markhamia platycalyx	WOA	0.27N	30.25E	Tro	T	Forest	NO	Bignoniaceae	Insect	9.00	28
217	Mimusops bagshawei	WOA	0.27N	30.25E	Tro	T	Forest	NO	Sapotaceae	Insect	5.00	28
218	Neoboutonia macrocalyx	WOA	0.27N	30.25E	Tro	T	Forest	NO	Euphorbiaceae	Insect	6.00	28
219	Olea welwitschii	WOA	0.27N	30.25E	Tro	T	Forest	NO	Oleaceae	Insect	7.00	28
220	Polyscias fulva	WOA	0.27N	30.25E	Tro	T	Forest	NO	Araliaceae	Insect	5.00	28
221	Premna angolensis	WOA	0.27N	30.25E	Tro	T	Forest	NO	Lamiaceae	Insect	8.00	28
222	Prunus africana	WOA	0.27N	30.25E	Tro	T	Forest	NO	Rosaceae	Insect	8.00	28
223	Strombosia scheffleri	WOA	0.27N	30.25E	Tro	T	Forest	NO	Olacaceae	Insect	22.00	28
224	Strychnos mitis	WOA	0.27N	30.25E	Tro	T	Forest	NO	Loganiaceae	Insect	2.00	28
225	Trilepisium phoberos	WOA	0.27N	30.25E	Tro	T	Forest	NO	Moraceae	Insect	3.00	28
226	Uvariopsis congensis	WOA	0.27N	30.25E	Tro	T	Forest	NO	Annonaceae	Insect	3.00	28
227	Culcasia falcifolia	WOA	0.34N	30.21E	Tro	V	Forest	NO	Araceae	Insect	9.70	77
228	Dracaena fragrans	WOA	0.34N	30.21E	Tro	S	Forest	NO	Asparagaceae	Insect	5.10	77

229	Dracaena laxissima	WOA	0.34N	30.21E	Tro	S	Forest	NO	Asparagaceae	Insect	8.70	77
230	Leptaspis zeylanica	WOA	0.34N	30.21E	Tro	H	Forest	NO	Poaceae	Insect	4.20	77
231	Palisota mannii	WOA	0.34N	30.21E	Tro	H	Forest	NO	Commelinaceae	Insect	3.70	77
232	Pollia condensata	WOA	0.34N	30.21E	Tro	H	Forest	NO	Commelinaceae	Insect	14.50	77
233	Hanguana malayana	WOA	1.21N	103.47E	Tro	H	Forest	NO	Hanguanaceae	Insect	0.51	77
234	Molineria latifolia	WOA	1.21N	103.47E	Tro	H	Forest	NO	Hypoxidaceae	Insect	1.50	77
235	Schismatoglottis wallichii	WOA	1.21N	103.47E	Tro	H	Forest	NO	Araceae	Insect	1.10	77
236	Tacca integrifolia	WOA	1.21N	103.47E	Tro	H	Forest	NO	Dioscoreaceae	Insect	2.90	77
237	Avicennia marina	WOA	1.43N	110.27E	Tro	T	Forest	NO	Acanthaceae	Insect	10.20	36
238	Eugenia ochneocarpa	WOA	1.43N	110.27E	Tro	T	Forest	NO	Myrtaceae	Insect	5.16	36
239	Sonneratia alba	WOA	1.43N	110.27E	Tro	T	Forest	NO	Lythraceae	Insect	14.30	36
240	Dryobalanops lanceolata	WOA	4.54N	117.48E	Tro	T	Forest	NO	Dipterocarpaceae	Insect	6.79	124
241	Hopea nervosa	WOA	4.54N	117.48E	Tro	T	Forest	NO	Dipterocarpaceae	Insect	23.56	124
242	Parashorea malaanonan	WOA	4.54N	117.48E	Tro	T	Forest	NO	Dipterocarpaceae	Insect	3.55	124
243	Shorea gibbosa	WOA	4.54N	117.48E	Tro	T	Forest	NO	Dipterocarpaceae	Insect	6.84	124
244	Dryobalanops lanceolata	WOA	4.571N	117.4E	Tro	T	Forest	NO	Dipterocarpaceae	NA	2.48	83
245	Dryobalanops lanceolata	WOA	4.58N	117.48E	Tro	T	Forest	NO	Dipterocarpaceae	Insect	2.52	17
246	Parashorea spp.	WOA	4.58N	117.48E	Tro	T	Forest	NO	Dipterocarpaceae	Insect	6.06	17
247	Shorea acuminatissima	WOA	4.58N	117.48E	Tro	T	Forest	NO	Dipterocarpaceae	Insect	2.52	17
248	Shorea glaucescens	WOA	4.58N	117.48E	Tro	T	Forest	NO	Dipterocarpaceae	Insect	1.33	17
249	Shorea johorensis	WOA	4.58N	117.48E	Tro	T	Forest	NO	Dipterocarpaceae	Insect	7.93	17
250	Shorea parvifolia	WOA	4.58N	117.48E	Tro	T	Forest	NO	Dipterocarpaceae	Insect	6.59	17
251	Hopea beccariana	WOA	5.1N	117.56E	Tro	T	Forest	NO	Dipterocarpaceae	NA	5.04	49
252	Hopea nervosa	WOA	5.1N	117.56E	Tro	T	Forest	NO	Dipterocarpaceae	NA	11.21	49
253	Parashorea tomentella	WOA	5.1N	117.56E	Tro	T	Forest	NO	Dipterocarpaceae	NA	12.11	49
254	Shorea fallax	WOA	5.1N	117.56E	Tro	T	Forest	NO	Dipterocarpaceae	NA	15.94	49
255	Shorea multiflora	WOA	5.1N	117.56E	Tro	T	Forest	NO	Dipterocarpaceae	NA	6.06	49
256	Arrabidaea candicans	WOA	8.59N	79.33W	Tro	L	Forest	NO	Bignoniaceae	Insect	0.71	195
257	Arrabidaea patellifera	WOA	8.59N	79.33W	Tro	L	Forest	NO	Bignoniaceae	Insect	3.49	195
258	Castilla elastica	WOA	8.59N	79.33W	Tro	T	Forest	NO	Moraceae	Insect	8.18	195
259	Cecropia longipes	WOA	8.59N	79.33W	Tro	T	Forest	NO	Moraceae	Insect	4.78	195
260	Chrysophyllum cainito	WOA	8.59N	79.33W	Tro	T	Forest	NO	Sapotaceae	Insect	16.17	195
261	Ficus insipida	WOA	8.59N	79.33W	Tro	T	Forest	NO	Moraceae	Insect	1.35	195

262	Luehea seemannii	WOA	8.59N	79.33W	Tro	T	Forest	NO	Malvaceae	Insect	5.38	195
263	Mikania leiostachya	WOA	8.59N	79.33W	Tro	V	Forest	NO	Asteraceae	Insect	1.33	195
264	Phoebe cinnamomifolia	WOA	8.59N	79.33W	Tro	T	Forest	NO	Lauraceae	Insect	8.73	195
265	Phryganocydia corymbosa	WOA	8.59N	79.33W	Tro	V	Forest	NO	Bignoniaceae	Insect	1.24	195
266	Stigmaphyllon hypargyreum	WOA	8.59N	79.33W	Tro	T	Forest	NO	Malpighiaceae	Insect	3.64	195
267	Urera caracasana	WOA	8.59N	79.33W	Tro	T	Forest	NO	Urticaceae	Insect	9.88	195
268	Bridelia ferruginea	WOA	9.06N	3.42W	Tro	T	Savanna	NO	Phyllanthaceae	Insect	6.60	194
269	Burkea africana	WOA	9.06N	3.42W	Tro	T	Savanna	NO	Fabaceae	Insect	3.40	194
270	Crossopteryx febrifuga	WOA	9.06N	3.42W	Tro	T	Savanna	NO	Rubiaceae	Insect	6.10	194
271	Detarium microcarpum	WOA	9.06N	3.42W	Tro	T	Savanna	NO	Fabaceae	Insect	8.70	194
272	Mitragyna inermis	WOA	9.06N	3.42W	Tro	T	Savanna	NO	Rubiaceae	Insect	2.30	194
273	Monotes kerstingii	WOA	9.06N	3.42W	Tro	T	Savanna	NO	Dipterocarpaceae	Insect	4.50	194
274	Piliostigma thonningii	WOA	9.06N	3.42W	Tro	T	Savanna	NO	Fabaceae	Insect	8.70	194
275	Sarcocephalus latifolia	WOA	9.06N	3.42W	Tro	S	Savanna	NO	Rubiaceae	Insect	6.90	194
276	Terminalia macroptera	WOA	9.06N	3.42W	Tro	T	Savanna	NO	Combretaceae	Insect	5.30	194
277	Acalypha diversifolia	WOA	9.08N	79.5W	Tro	S	Forest	NO	Euphorbiaceae	Insect	11.69	162
278	Alseis blackiana	WOA	9.08N	79.5W	Tro	T	Forest	NO	Rubiaceae	Insect	39.37	162
279	Annona spraguei	WOA	9.08N	79.5W	Tro	T	Forest	NO	Annonaceae	Insect	36.03	162
280	Cecropia insignis	WOA	9.08N	79.5W	Tro	T	Forest	NO	Moraceae	Insect	37.40	162
281	Coussarea curvigemmia	WOA	9.08N	79.5W	Tro	T	Forest	NO	Rubiaceae	Insect	4.40	162
282	Faramea occidentalis	WOA	9.08N	79.5W	Tro	T	Forest	NO	Rubiaceae	Insect	26.19	162
283	Garcinia edulis	WOA	9.08N	79.5W	Tro	T	Forest	NO	Clusiaceae	Insect	6.10	162
284	Hybanthus prunifolius	WOA	9.08N	79.5W	Tro	S	Forest	NO	Violaceae	Insect	14.51	162
285	Luehea seemannii	WOA	9.08N	79.5W	Tro	T	Forest	NO	Malvaceae	Insect	48.37	162
286	Miconia argentea	WOA	9.08N	79.5W	Tro	T	Forest	NO	Melastomataceae	Insect	43.45	162
287	Mouriri myrtilloides	WOA	9.08N	79.5W	Tro	S	Forest	NO	Melastomataceae	Insect	17.53	162
288	Pouteria stipitata	WOA	9.08N	79.5W	Tro	T	Forest	NO	Sapotaceae	Insect	14.99	162
289	Protium tenuifolium	WOA	9.08N	79.5W	Tro	T	Forest	NO	Burseraceae	Insect	10.25	162
290	Psychotria acuminata	WOA	9.08N	79.5W	Tro	T	Forest	NO	Rubiaceae	Insect	3.51	162
291	Psychotria furcata	WOA	9.08N	79.5W	Tro	S	Forest	NO	Rubiaceae	Insect	15.51	162
292	Psychotria horizontalis	WOA	9.08N	79.5W	Tro	S	Forest	NO	Rubiaceae	Insect	26.47	162
293	Psychotria marginata	WOA	9.08N	79.5W	Tro	S	Forest	NO	Rubiaceae	Insect	18.06	162
294	Rinorea sylvatica	WOA	9.08N	79.5W	Tro	S	Forest	NO	Violaceae	Insect	13.18	162

295	Spondias mombin	WOA	9.08N	79.5W	Tro	T	Forest	NO	Anacardiaceae	Insect	83.35	162
296	Tachigali versicolor	WOA	9.08N	79.5W	Tro	T	Forest	NO	Fabaceae	Insect	66.40	162
297	Tetragastris panamensis	WOA	9.08N	79.5W	Tro	T	Forest	NO	Burseraceae	Insect	9.52	162
298	Chusquea simpliciflora	WOA	9.09N	79.51W	Tro	V	Forest	NO	Poaceae	Insect	1.80	77
299	Connarus turczaninowii	WOA	9.09N	79.51W	Tro	V	Forest	NO	Connaraceae	Insect	9.61	7
300	Desmoncus orthocanthos	WOA	9.09N	79.51W	Tro	V	Forest	NO	Arecaceae	Insect	16.70	77
301	Inga acuminata	WOA	9.09N	79.51W	Tro	T	Forest	NO	Fabaceae	Insect	23.30	106
302	Inga cocleensis	WOA	9.09N	79.51W	Tro	T	Forest	NO	Fabaceae	Insect	23.60	106
303	Inga laurina	WOA	9.09N	79.51W	Tro	T	Forest	NO	Fabaceae	Insect	31.40	106
304	Inga marginata	WOA	9.09N	79.51W	Tro	T	Forest	NO	Fabaceae	Insect	40.60	106
305	Inga multijuga	WOA	9.09N	79.51W	Tro	T	Forest	NO	Fabaceae	Insect	42.50	106
306	Inga nobilis	WOA	9.09N	79.51W	Tro	T	Forest	NO	Fabaceae	Insect	24.30	106
307	Inga pezizifera	WOA	9.09N	79.51W	Tro	T	Forest	NO	Fabaceae	Insect	25.40	106
308	Inga sapindoides	WOA	9.09N	79.51W	Tro	T	Forest	NO	Fabaceae	Insect	22.40	106
309	Inga umbellifera	WOA	9.09N	79.51W	Tro	T	Forest	NO	Fabaceae	Insect	21.40	35
310	Inga umbellifera	WOA	9.09N	79.51W	Tro	T	Forest	NO	Fabaceae	Insect	20.50	106
311	Inga vera	WOA	9.09N	79.51W	Tro	T	Forest	NO	Fabaceae	Insect	34.40	106
312	Pharus latifolius	WOA	9.09N	79.51W	Tro	H	Forest	NO	Poaceae	Insect	6.10	77
313	Rhipidocladum racemiflorum	WOA	9.09N	79.51W	Tro	H	Forest	NO	Poaceae	Insect	0.14	77
314	Vriesea sanguinolenta	WOA	9.1N	79.51W	Tro	H	Forest	NO	Bromeliaceae	Insect	2.83	172
315	Anacardium excelsum	WOA	9.193N	79.38W	Tro	T	Field	NO	Anacardiaceae	Insect	6.32	156
316	Anacardium excelsum	WOA	9.213N	79.54W	Tro	T	Plantation	NO	Anacardiaceae	Insect	2.04	129
317	Dalbergia retusa	WOA	9.213N	79.54W	Tro	T	Plantation	NO	Fabaceae	Insect	1.86	129
318	Pachira quinata	WOA	9.213N	79.54W	Tro	T	Plantation	NO	Malvaceae	Insect	1.49	129
319	Terminalia amazonia	WOA	9.213N	79.54W	Tro	T	Plantation	NO	Combretaceae	Insect	1.85	129
320	Carica papaya	WOA	9.51N	83.39W	Tro	T	Fallow field	NO	Caricaceae	Insect	2.50	52
321	Clibadium sp.	WOA	9.51N	83.39W	Tro	S	Fallow field	NO	Asteraceae	Insect	4.20	52
322	Erythrina poeppigiana	WOA	9.51N	83.39W	Tro	T	Agricultural	NO	Fabaceae	Insect	5.50	52
323	Phaseolus vulgaris	WOA	9.51N	83.39W	Tro	V	Fallow field	NO	Fabaceae	Insect	1.70	52
324	Phytolacca rivinoides	WOA	9.51N	83.39W	Tro	H	Fallow field	NO	Phytolaccaceae	Insect	10.70	52
325	Sorghum bicolor	WOA	9.51N	83.39W	Tro	H	Fallow field	NO	Poaceae	Insect	16.40	52
326	Adiantum obliquum	WOA	10.25N	84W	Tro	H	Forest	NO	Adiantaceae	Insect	7.30	86
327	Polybotrya cervina	WOA	10.25N	84W	Tro	H	Forest	NO	Dryopteridaceae	Insect	9.90	86

328	Thelypteris turrialbae	WOA	10.25N	84W	Tro	H	Forest	NO	Thelypteridaceae	Insect	5.50	86
329	Hampea appendiculata	WOA	10.25N	84.05W	Tro	T	Forest	NO	Malvaceae	NA	27.35	121
330	Hampea appendiculata	WOA	10.25N	84.05W	Tro	T	Grassland	NO	Malvaceae	NA	21.25	121
331	Asplundia uncinata	WOA	10.26N	84W	Tro	H	Forest	NO	Cyclanthaceae	Insect	3.50	77
332	Cyclanthus bipartitus	WOA	10.26N	84W	Tro	H	Forest	NO	Cyclanthaceae	Insect	22.20	77
333	Geonoma congesta	WOA	10.26N	84W	Tro	S	Forest	NO	Arecaceae	Insect	13.40	77
334	Spathiphyllum fulvovirens	WOA	10.26N	84W	Tro	H	Forest	NO	Araceae	Insect	22.10	77
335	Welfia regia	WOA	10.26N	84W	Tro	T	Forest	NO	Arecaceae	Insect	3.50	77
336	Asclepias curassavica	WOA	10.42N	61.17W	Tro	S	Forest	NO	Apocynaceae	NA	0.80	206
337	Cassia fruticosa	WOA	10.42N	61.17W	Tro	T	Forest	NO	Fabaceae	NA	5.00	206
338	Chrysothemis pulchella	WOA	10.42N	61.17W	Tro	H	Forest	NO	Gesneriaceae	NA	2.17	206
339	Lantana camara	WOA	10.42N	61.17W	Tro	V	Forest	NO	Verbenaceae	NA	5.58	206
340	Laurentia longiflora	WOA	10.42N	61.17W	Tro	H	Forest	NO	Campanulaceae	NA	0.20	206
341	Mimosa pudica	WOA	10.42N	61.17W	Tro	H	Forest	NO	Fabaceae	NA	2.27	206
342	Psiguria umbrosa	WOA	10.42N	61.17W	Tro	V	Forest	NO	Cucurbitaceae	NA	0.67	206
343	Spermacoce assurgens	WOA	10.42N	61.17W	Tro	S	Forest	NO	Rubiaceae	NA	2.20	206
344	Stachytarpheta jamaicensis	WOA	10.42N	61.17W	Tro	H	Forest	NO	Verbenaceae	NA	8.33	206
345	Trimezia martinicensis	WOA	10.42N	61.17W	Tro	H	Forest	NO	Iridaceae	NA	0.20	206
346	Brassica oleracea	WOA	12.59N	86.21W	Tro	H	Agricultural	NO	Brassicaceae	Insect	14.15	23
347	Dialium guianense	WOA	16.27N	91.93W	Tro	T	Forest	NO	Fabaceae	NA	5.81	22
348	Casearia arborea	WOA	18.1N	65.3W	Tro	T	Forest	NO	Salicaceae	Insect	0.31	174
349	Dacryodes excelsa	WOA	18.1N	65.3W	Tro	T	Forest	NO	Burseraceae	Insect	0.68	174
350	Manilkara bidentata	WOA	18.1N	65.3W	Tro	T	Forest	NO	Sapotaceae	Insect	2.16	174
351	Sloanea berteriana	WOA	18.1N	65.3W	Tro	T	Forest	NO	Elaeocarpaceae	Insect	4.13	174
352	Cecropia schreberiana	WOA	18.2N	66W	Tro	T	Forest	NO	Moraceae	Insect	17.00	137
353	Inga vera	WOA	18.2N	66W	Tro	T	Forest	NO	Fabaceae	Insect	18.00	137
354	Chamaedorea alternans	WOA	18.35N	95.07W	Tro	T	Forest	NO	Arecaceae	Insect	15.08	34
355	Chamaedorea ernesti-augusti	WOA	18.35N	95.07W	Tro	T	Forest	NO	Arecaceae	Insect	0.49	34
356	Chamaedorea pinnatifrons	WOA	18.35N	95.07W	Tro	T	Forest	NO	Arecaceae	Insect	2.14	34
357	Nectandra ambigens	WOA	18.35N	95.07W	Tro	T	Forest	NO	Lauraceae	Insect	20.70	67
358	Allophylus campostachys	WOA	18.36N	95W	Tro	T	Forest	NO	Sapindaceae	Insect	8.20	40
359	Bursera simaruba	WOA	18.36N	95W	Tro	T	Forest	NO	Burseraceae	Insect	0.16	40
360	Costus villosissimus	WOA	18.36N	95W	Tro	H	Forest	NO	Costaceae	Insect	9.74	40

361	Cupania dentata	WOA	18.36N	95W	Tro	T	Forest	NO	Sapindaceae	Insect	13.10	40
362	Paullinia spp.	WOA	18.36N	95W	Tro	L	Forest	NO	Sapindaceae	Insect	10.44	40
363	Solanum spp.	WOA	18.36N	95W	Tro	H	Forest	NO	Solanaceae	Insect	9.18	40
364	Spondias radlkoferi	WOA	18.36N	95W	Tro	T	Forest	NO	Anacardiaceae	Insect	6.00	40
365	Thinouia tomocarpa	WOA	18.36N	95W	Tro	L	Forest	NO	Sapindaceae	Insect	13.90	40
366	Abuta panamensis	WOA	18.36N	95.07W	Tro	L	Forest	NO	Menispermaceae	Insect	13.00	40
367	Acalypha skutchii	WOA	18.36N	95.07W	Tro	S	Forest	NO	Euphorbiaceae	Insect	24.00	40
368	Ampelocera hottlei	WOA	18.36N	95.07W	Tro	T	Forest	NO	Ulmaceae	Insect	9.11	40
369	Aphelandra aurantiaca	WOA	18.36N	95.07W	Tro	T	Forest	NO	Acanthaceae	Insect	6.16	40
370	Astrocaryum mexicanum	WOA	18.36N	95.07W	Tro	T	Forest	NO	Arecaceae	Insect	13.50	40
371	Brosimum alicastrum	WOA	18.36N	95.07W	Tro	T	Forest	NO	Moraceae	Insect	5.70	40
372	Calophyllum brasiliense	WOA	18.36N	95.07W	Tro	T	Forest	NO	Clusiaceae	Insect	4.07	40
373	Carica papaya	WOA	18.36N	95.07W	Tro	T	Forest	NO	Caricaceae	Insect	7.60	40
374	Cecropia obtusifolia	WOA	18.36N	95.07W	Tro	T	Forest	NO	Moraceae	Insect	5.93	40
375	Chamaedorea spp.	WOA	18.36N	95.07W	Tro	T	Forest	NO	Arecaceae	Insect	3.50	40
376	Connarus schultesii	WOA	18.36N	95.07W	Tro	L	Forest	NO	Connaraceae	Insect	9.50	40
377	Croton schiedeanus	WOA	18.36N	95.07W	Tro	T	Forest	NO	Euphorbiaceae	Insect	12.70	40
378	Cymbopetalum baillonii	WOA	18.36N	95.07W	Tro	T	Forest	NO	Annonaceae	Insect	3.80	40
379	Cynometra retusa	WOA	18.36N	95.07W	Tro	T	Forest	NO	Fabaceae	Insect	6.50	40
380	Dussia mexicana	WOA	18.36N	95.07W	Tro	T	Forest	NO	Fabaceae	Insect	18.86	40
381	Eugenia spp.	WOA	18.36N	95.07W	Tro	T	Forest	NO	Myrtaceae	Insect	9.08	40
382	Faramea occidentalis	WOA	18.36N	95.07W	Tro	T	Forest	NO	Rubiaceae	Insect	12.89	40
383	Garcinia edulis	WOA	18.36N	95.07W	Tro	T	Forest	NO	Clusiaceae	Insect	9.78	40
384	Hamelia longipes	WOA	18.36N	95.07W	Tro	T	Forest	NO	Rubiaceae	Insect	13.30	40
385	Heliocarpus appendiculatus	WOA	18.36N	95.07W	Tro	T	Forest	NO	Malvaceae	Insect	11.50	40
386	Inga spp.	WOA	18.36N	95.07W	Tro	T	Forest	NO	Fabaceae	Insect	6.50	40
387	Lonchocarpus guatemalensis	WOA	18.36N	95.07W	Tro	T	Forest	NO	Fabaceae	Insect	3.21	40
388	Nectandra ambigens	WOA	18.36N	95.07W	Tro	T	Forest	NO	Lauraceae	Insect	5.10	40
389	Omphalea oleifera	WOA	18.36N	95.07W	Tro	T	Forest	NO	Euphorbiaceae	Insect	19.80	40
390	Orthion oblanceolatum	WOA	18.36N	95.07W	Tro	T	Forest	NO	Violaceae	Insect	6.42	40
391	Poulsenia armata	WOA	18.36N	95.07W	Tro	T	Forest	NO	Moraceae	Insect	1.78	40
392	Pseudolmedia oxyphyllaria	WOA	18.36N	95.07W	Tro	T	Forest	NO	Moraceae	Insect	7.10	40
393	Psychotria faxlucens	WOA	18.36N	95.07W	Tro	T	Forest	NO	Rubiaceae	Insect	3.76	40

394	Psychotria simiarum	WOA	18.36N	95.07W	Tro	T	Forest	NO	Rubiaceae	Insect	0.97	40
395	Pterocarpus rohrii	WOA	18.36N	95.07W	Tro	T	Forest	NO	Fabaceae	Insect	6.30	40
396	Robinsonella mirandae	WOA	18.36N	95.07W	Tro	T	Forest	NO	Malvaceae	Insect	7.04	40
397	Salacia cordata	WOA	18.36N	95.07W	Tro	L	Forest	NO	Celastraceae	Insect	9.80	40
398	Schaueria calycobractea	WOA	18.36N	95.07W	Tro	S	Forest	NO	Acanthaceae	Insect	14.20	40
399	Stemmadenia donnellsmithii	WOA	18.36N	95.07W	Tro	T	Forest	NO	Apocynaceae	Insect	4.88	40
400	Strychnos tabascana	WOA	18.36N	95.07W	Tro	L	Forest	NO	Loganiaceae	Insect	8.60	40
401	Trema micrantha	WOA	18.36N	95.07W	Tro	T	Forest	NO	Cannabaceae	Insect	3.17	40
402	Trophis mexicana	WOA	18.36N	95.07W	Tro	T	Forest	NO	Moraceae	Insect	0.65	40
403	Urera caracasana	WOA	18.36N	95.07W	Tro	T	Forest	NO	Urticaceae	Insect	18.01	40
404	Vatairea lundellii	WOA	18.36N	95.07W	Tro	T	Forest	NO	Fabaceae	Insect	13.50	40
405	Pinguicula moranensis	WOA	18.58N	99.17W	T	H	Forest	NO	Lentibulariaceae	Insect	1.61	9
406	Blechnum varians	WOA	19.3N	96.57W	T	H	Forest	NO	Blechnaceae	NA	9.31	126
407	Diplazium expansum	WOA	19.3N	96.57W	T	H	Forest	NO	Woodsiaceae	NA	10.84	126
408	Marattia laxa	WOA	19.3N	96.57W	T	H	Forest	NO	Marattiaceae	NA	14.81	126
409	Pleopeltis crassinervata	WOA	19.3N	96.57W	T	H	Grassland	NO	Polypodiaceae	Insect	13.15	127
410	Polypodium furfuraceum	WOA	19.3N	96.57W	T	H	Grassland	NO	Polypodiaceae	Insect	9.58	127
411	Polypodium polypodioides	WOA	19.3N	96.57W	T	H	Grassland	NO	Polypodiaceae	Insect	12.56	127
412	Polypodium rhodopleuron	WOA	19.3N	96.57W	T	H	Grassland	NO	Polypodiaceae	Insect	9.50	127
413	Bursera excelsa	WOA	19.3N	105W	Tro	T	Forest	NO	Burseraceae	Insect	12.16	38
414	Bursera heteresthes	WOA	19.3N	105W	Tro	T	Forest	NO	Burseraceae	Insect	3.12	56
415	Bursera instabilis	WOA	19.3N	105W	Tro	T	Forest	NO	Burseraceae	NA	4.57	20
416	Cordia elaeagnoides	WOA	19.3N	105W	Tro	T	Forest	NO	Boraginaceae	Insect	3.49	56
417	Ipomoea wolcottiana	WOA	19.3N	105W	Tro	T	Forest	NO	Convolvulaceae	Insect	66.84	38
418	Ipomoea wolcottiana	WOA	19.3N	105W	Tro	T	Forest	NO	Convolvulaceae	Insect	14.00	56
419	Thouinia paucidentata	WOA	19.3N	105W	Tro	T	Forest	NO	Sapindaceae	Insect	6.74	56
420	Trichilia trifolia	WOA	19.3N	105W	Tro	T	Forest	NO	Meliaceae	Insect	5.86	56
421	Achatocarpus gracilis	WOA	19.3N	105.03W	Tro	S	Forest	NO	Achatocarpaceae	Insect	16.18	38
422	Brosimum alicastrum	WOA	19.3N	105.03W	Tro	T	Forest	NO	Moraceae	Insect	12.75	38
423	Caesalpinia caladenia	WOA	19.3N	105.03W	Tro	S	Forest	NO	Fabaceae	Insect	36.03	38
424	Casearia	WOA	19.3N	105.03W	Tro	T	Forest	NO	Salicaceae	Insect	11.32	56
425	Casearia nitida	WOA	19.3N	105.03W	Tro	T	Forest	NO	Salicaceae	Insect	9.66	21
426	Ceiba aesculifolia	WOA	19.3N	105.03W	Tro	T	Forest	NO	Malvaceae	Insect	10.58	38

427	Ceiba grandiflora	WOA	19.3N	105.03W	Tro	T	Forest	NO	Malvaceae	Insect	11.06	38
428	Celaenodendron mexicanun	WOA	19.3N	105.03W	Tro	T	Forest	NO	Picrodendraceae	Insect	9.86	56
429	Croton alamosanus	WOA	19.3N	105.03W	Tro	S	Forest	NO	Euphorbiaceae	Insect	12.84	38
430	Croton pseudoniveus	WOA	19.3N	105.03W	Tro	S	Forest	NO	Euphorbiaceae	Insect	11.79	38
431	Croton pseudoniveus	WOA	19.3N	105.03W	Tro	T	Forest	NO	Euphorbiaceae	Insect	15.16	56
432	Croton pseudoniveus	WOA	19.3N	105.03W	Tro	T	Forest	NO	Euphorbiaceae	NA	10.91	20
433	Croton suberosus	WOA	19.3N	105.03W	Tro	S	Forest	NO	Euphorbiaceae	Insect	11.93	38
434	Cynometra oaxacana	WOA	19.3N	105.03W	Tro	T	Forest	NO	Fabaceae	Insect	19.20	38
435	Erythroxylum mexicanum	WOA	19.3N	105.03W	Tro	T	Forest	NO	Rhizophoraceae	Insect	8.00	38
436	Ficus cotinifolia	WOA	19.3N	105.03W	Tro	T	Forest	NO	Moraceae	Insect	12.50	38
437	Guapira macrocarpa	WOA	19.3N	105.03W	Tro	T	Forest	NO	Nyctaginaceae	Insect	14.92	38
438	Guapira spp.	WOA	19.3N	105.03W	Tro	T	Forest	NO	Nyctaginaceae	Insect	13.36	56
439	Guettarda elliptica	WOA	19.3N	105.03W	Tro	S	Forest	NO	Rubiaceae	Insect	12.37	38
440	Gyrocarpus jatrophifolius	WOA	19.3N	105.03W	Tro	T	Forest	NO	Hernandiaceae	Insect	73.68	38
441	Heliocarpus pallidus	WOA	19.3N	105.03W	Tro	T	Forest	NO	Malvaceae	Insect	17.55	37
442	Heliocarpus pallidus	WOA	19.3N	105.03W	Tro	T	Forest	NO	Malvaceae	Insect	8.48	38
443	Heliocarpus pallidus	WOA	19.3N	105.03W	Tro	T	Forest	NO	Malvaceae	Insect	12.29	56
444	Jatropha malacophylla	WOA	19.3N	105.03W	Tro	T	Forest	NO	Euphorbiaceae	Insect	7.49	38
445	Jatropha standleyi	WOA	19.3N	105.03W	Tro	T	Forest	NO	Euphorbiaceae	Insect	13.56	38
446	Jatropha standleyi	WOA	19.3N	105.03W	Tro	T	Forest	NO	Euphorbiaceae	Insect	28.09	56
447	Lippia graveolens	WOA	19.3N	105.03W	Tro	S	Forest	NO	Verbenaceae	Insect	4.21	38
448	Lonchocarpus eriocarinalis	WOA	19.3N	105.03W	Tro	T	Forest	NO	Fabaceae	Insect	12.15	38
449	Lonchocarpus eriocarinalis	WOA	19.3N	105.03W	Tro	T	Forest	NO	Fabaceae	Insect	4.88	56
450	Lonchocarpus spp.	WOA	19.3N	105.03W	Tro	S	Forest	NO	Fabaceae	Insect	11.27	38
451	Maclura tinctoria	WOA	19.3N	105.03W	Tro	T	Forest	NO	Moraceae	Insect	13.23	38
452	Piper stipulaceum	WOA	19.3N	105.03W	Tro	S	Forest	NO	Piperaceae	NA	12.27	20
453	Psidium sartorianum	WOA	19.3N	105.03W	Tro	T	Forest	NO	Myrtaceae	Insect	2.94	56
454	Randia spinosa	WOA	19.3N	105.03W	Tro	S	Forest	NO	Rubiaceae	Insect	26.16	38
455	Recchia mexicana	WOA	19.3N	105.03W	Tro	S	Forest	NO	Surianaceae	Insect	9.60	38
456	Ruprechtia fusca	WOA	19.3N	105.03W	Tro	T	Forest	NO	Polygonaceae	Insect	20.24	38
457	Thevetia ovata	WOA	19.3N	105.03W	Tro	S	Forest	NO	Apocynaceae	Insect	7.80	38
458	Urera caracasana	WOA	19.3N	105.03W	Tro	T	Forest	NO	Urticaceae	Insect	11.20	38
459	Vitex hemsleyi	WOA	19.3N	105.03W	Tro	S	Forest	NO	Lamiaceae	Insect	16.10	38

460	Catopsis sessiliflora	WOA	19.31N	96.57W	T	H	Grassland	NO	Bromeliaceae	NA	55.00	200
461	Jacquiniella leucomelana	WOA	19.31N	96.57W	T	H	Grassland	NO	Orchidaceae	NA	52.00	200
462	Jacquiniella teretifolia	WOA	19.31N	96.57W	T	H	Grassland	NO	Orchidaceae	NA	60.00	200
463	Lycaste aromatica	WOA	19.31N	96.57W	T	H	Grassland	NO	Orchidaceae	NA	40.00	200
464	Pleopeltis crassinervata	WOA	19.31N	96.57W	T	H	Grassland	NO	Polypodiaceae	NA	8.53	200
465	Pleopeltis plebeium	WOA	19.31N	96.57W	T	H	Grassland	NO	Polypodiaceae	NA	16.97	200
466	Pleopeltis polypodioides	WOA	19.31N	96.57W	T	V	Grassland	NO	Polypodiaceae	NA	8.07	200
467	Pleopeltis rhodopleuron	WOA	19.31N	96.57W	T	H	Grassland	NO	Polypodiaceae	NA	7.71	200
468	Polypodium furfuraceum	WOA	19.31N	96.57W	T	H	Grassland	NO	Polypodiaceae	NA	6.42	200
469	Tillandsia deppeana	WOA	19.31N	96.57W	T	H	Grassland	NO	Bromeliaceae	NA	73.00	200
470	Tillandsia juncea	WOA	19.31N	96.57W	T	H	Grassland	NO	Bromeliaceae	NA	20.00	200
471	Tillandsia multicaulis	WOA	19.31N	96.57W	T	H	Grassland	NO	Bromeliaceae	NA	41.00	200
472	Tillandsia punctulata	WOA	19.31N	96.57W	T	H	Grassland	NO	Bromeliaceae	NA	65.00	200
473	Begonia spp.1	WOA	19.37N	97.44W	T	H	Forest	NO	Begoniaceae	Insect	5.00	15
474	Begonia spp.2	WOA	19.37N	97.44W	T	H	Forest	NO	Begoniaceae	Insect	7.00	15
475	Blechnum varians	WOA	19.37N	97.44W	T	H	Forest	NO	Blechnaceae	Insect	12.00	15
476	Gunnera mexicana	WOA	19.37N	97.44W	T	H	Forest	NO	Gunneraceae	Insect	4.00	15
477	Polystichum muricatum	WOA	19.37N	97.44W	T	H	Forest	NO	Dryopteridaceae	Insect	16.00	15
478	Thelypteris cheilanthoides	WOA	19.37N	97.44W	T	H	Forest	NO	Thelypteridaceae	Insect	38.00	15
479	Achatocarpus gracilis	WOA	20.91N	103.54W	T	S	Forest	NO	Achatocarpaceae	Insect	6.15	149
480	Guapira macrocarpa	WOA	20.91N	103.54W	T	T	Forest	NO	Nyctaginaceae	Insect	8.55	149
481	Guettarda elliptica	WOA	20.91N	103.54W	T	S	Forest	NO	Rubiaceae	Insect	7.75	149
482	Ruprechtia fusca	WOA	20.91N	103.54W	T	T	Forest	NO	Polygonaceae	Insect	5.55	149
483	Bursera simaruba	WOA	21.06N	89.1W	A	T	Forest	NO	Burseraceae	NA	3.95	31
484	Acacia gaumeri	WOA	21.06N	89.17W	A	T	Forest	NO	Fabaceae	NA	2.45	31
485	Pithecellobium dulce	WOA	21.06N	89.17W	A	T	Forest	NO	Fabaceae	NA	4.57	31
486	Coccoloba uvifera	WOA	24.1N	76.26W	Tro	S	Grassland	NO	Polygonaceae	Insect	0.79	186
487	Amaranthus hypochondriacus	WOA	24.44N	101.19W	A	H	Agricultural	NO	Amaranthaceae	Insect	9.80	41
488	Brassica oleracea	WOA	26.93N	97.59W	T	H	Agricultural	NO	Brassicaceae	Insect	18.50	111
489	Avicennia germinans	WOA	27.45N	82.34W	T	T	Mangrove	NO	Acanthaceae	Others	0.50	50
490	Rhizophora mangle	WOA	27.45N	82.34W	T	T	Mangrove	NO	Rhizophoraceae	Others	7.64	50
491	Ardisia crenata	WOA	29.14N	118.07E	T	S	Forest	NO	Primulaceae	Insect	4.06	176
492	Ardisia crenata	WOA	29.14N	118.07E	T	S	Forest	NO	Primulaceae	Insect	1.75	177

493	Camellia fraterna	WOA	29.14N	118.07E	T	S	Forest	NO	Theaceae	Insect	3.36	176
494	Camellia fraterna	WOA	29.14N	118.07E	T	S	Forest	NO	Theaceae	Insect	2.99	177
495	Castanopsis carlesii	WOA	29.14N	118.07E	T	T	Forest	NO	Fagaceae	Insect	3.20	177
496	Castanopsis eyrei	WOA	29.14N	118.07E	T	T	Forest	NO	Fagaceae	Insect	7.33	176
497	Castanopsis eyrei	WOA	29.14N	118.07E	T	T	Forest	NO	Fagaceae	Insect	5.30	177
498	Castanopsis fargesii	WOA	29.14N	118.07E	T	T	Forest	NO	Fagaceae	Insect	5.30	177
499	Castanopsis tibetana	WOA	29.14N	118.07E	T	T	Forest	NO	Fagaceae	Insect	3.78	177
500	Cinnamomum subavenium	WOA	29.14N	118.07E	T	T	Forest	NO	Lauraceae	Insect	4.86	177
501	Cyclobalanopsis glauca	WOA	29.14N	118.07E	T	T	Forest	NO	Fagaceae	Insect	17.05	176
502	Cyclobalanopsis glauca	WOA	29.14N	118.07E	T	T	Forest	NO	Fagaceae	Insect	14.00	177
503	Cyclobalanopsis myrsinifolia	WOA	29.14N	118.07E	T	T	Forest	NO	Fagaceae	Insect	11.07	177
504	Eurya muricata	WOA	29.14N	118.07E	T	S	Forest	NO	Pentaphylacaceae	Insect	5.57	176
505	Eurya muricata	WOA	29.14N	118.07E	T	S	Forest	NO	Pentaphylacaceae	Insect	3.06	177
506	Eurya rubiginosa	WOA	29.14N	118.07E	T	S	Forest	NO	Pentaphylacaceae	Insect	2.84	177
507	Lithocarpus glaber	WOA	29.14N	118.07E	T	T	Forest	NO	Fagaceae	Insect	13.52	176
508	Lithocarpus glaber	WOA	29.14N	118.07E	T	T	Forest	NO	Fagaceae	Insect	10.05	177
509	Machilus thunbergii	WOA	29.14N	118.07E	T	T	Forest	NO	Lauraceae	Insect	8.30	176
510	Machilus thunbergii	WOA	29.14N	118.07E	T	T	Forest	NO	Lauraceae	Insect	3.92	177
511	Myrica rubra	WOA	29.14N	118.07E	T	T	Forest	NO	Myricaceae	Insect	3.27	177
512	Neolitsea aurata	WOA	29.14N	118.07E	T	T	Forest	NO	Lauraceae	Insect	8.92	176
513	Neolitsea aurata	WOA	29.14N	118.07E	T	T	Forest	NO	Lauraceae	Insect	4.14	177
514	Quercus serrata	WOA	29.14N	118.07E	T	T	Forest	NO	Fagaceae	Insect	11.22	177
515	Schima superba	WOA	29.14N	118.07E	T	T	Forest	NO	Theaceae	Insect	7.95	176
516	Schima superba	WOA	29.14N	118.07E	T	T	Forest	NO	Theaceae	Insect	5.73	177
517	Symplocos stellaris	WOA	29.14N	118.07E	T	S	Forest	NO	Symplocaceae	Insect	1.46	177
518	Loropetalum chinense	WOA	29.14N	118E	T	S	Forest	NO	Hamamelidaceae	Insect	6.10	176
519	Celtis laevigata	WOA	29.23N	95.02W	T	T	Forest	NO	Cannabaceae	Insect	12.99	180
520	Celtis laevigata	WOA	29.23N	95.02W	T	T	Grassland	NO	Cannabaceae	Insect	4.49	180
521	Fagus grandifolia	WOA	29.23N	95.02W	T	T	Forest	NO	Fagaceae	Insect	5.57	180
522	Sapium sebiferum	WOA	29.23N	95.02W	T	T	Forest	NO	Euphorbiaceae	Insect	5.70	180
523	Sapium sebiferum	WOA	29.23N	95.02W	T	T	Grassland	NO	Euphorbiaceae	Insect	3.42	180
524	Asimina incana	WOA	29.38N	82.2W	T	S	Sandhill	NO	Annonaceae	Insect	25.09	99
525	Croton argyranthemus	WOA	29.38N	82.2W	T	H	Sandhill	NO	Euphorbiaceae	Insect	7.27	99

526	Eriogonum tomentosum	WOA	29.38N	82.2W	T	H	Sandhill	NO	Polygonaceae	Insect	13.82	99
527	Licania michauxii	WOA	29.38N	82.2W	T	S	Sandhill	NO	Chrysobalanaceae	Insect	12.00	99
528	Quercus laevis	WOA	29.38N	82.2W	T	T	Sandhill	NO	Fagaceae	Insect	22.55	99
529	Rhynchosia reniformis	WOA	29.38N	82.2W	T	H	Sandhill	NO	Fabaceae	Insect	8.18	99
530	Tephrosia virginiana	WOA	29.38N	82.2W	T	H	Sandhill	NO	Fabaceae	Insect	14.00	99
531	Acer saccharinum	WOA	29.45N	95.2W	T	T	Plantation	NO	Sapindaceae	Others	1.49	82
532	Liquidambar styraciflua	WOA	29.45N	95.2W	T	T	Plantation	NO	Altingiaceae	Insect	3.03	82
533	Platanus occidentalis	WOA	29.45N	95.22W	T	T	Plantation	NO	Proteaceae	Insect	3.20	82
534	Sapium sebiferum	WOA	29.45N	95.22W	T	T	Plantation	NO	Euphorbiaceae	Insect	0.56	82
535	Glycine max	WOA	30.31N	87.54W	T	H	Agricultural	NO	Fabaceae	Insect	63.17	87
536	Glycine max	WOA	31.21N	85.2W	T	H	Agricultural	NO	Fabaceae	Insect	43.95	87
537	Glycine max	WOA	31.31N	87.19W	T	H	Agricultural	NO	Fabaceae	Insect	26.55	87
538	Glycine max	WOA	31.61N	87.41W	T	H	Agricultural	NO	Fabaceae	Insect	34.20	87
539	Brassica oleracea	WOA	32.26N	85.53W	T	H	Agricultural	NO	Brassicaceae	Insect	5.68	83
540	Glycine max	WOA	32.26N	87.14W	T	H	Agricultural	NO	Fabaceae	Insect	51.40	87
541	Persea americana	WOA	33.41N	117.47W	A	T	Agricultural	NO	Lauraceae	Others	26.00	97
542	Oenothera humifusa	WOA	33.53N	78.1W	T	H	Dune	NO	Onagraceae	NA	1.18	208
543	Spartina alterniflora	WOA	33.53N	78.1W	T	H	Marsh	NO	Poaceae	NA	0.00	208
544	Quercus prinus	WOA	33.53N	83.21W	T	T	Forest	NO	Fagaceae	Insect	14.07	60
545	Quercus rubra	WOA	33.53N	83.21W	T	T	Forest	NO	Fagaceae	Insect	21.16	60
546	Borrichia frutescens	WOA	33.57N	77.56W	T	S	Marsh	NO	Asteraceae	NA	0.40	208
547	Spartina alterniflora	WOA	33.57N	77.56W	T	H	Marsh	NO	Poaceae	NA	0.14	208
548	Oenothera humifusa	WOA	34.2N	77.53W	T	H	Dune	NO	Onagraceae	NA	3.07	208
549	Spartina alterniflora	WOA	34.2N	77.54W	T	H	Marsh	NO	Poaceae	NA	0.03	208
550	Oenothera humifusa	WOA	34.25N	77.32W	T	H	Dune	NO	Onagraceae	NA	0.11	208
551	Borrichia frutescens	WOA	34.29N	77.25W	T	S	Marsh	NO	Asteraceae	NA	0.72	208
552	Solidago sempervirens	WOA	34.29N	77.25W	T	H	Marsh	NO	Asteraceae	NA	4.21	208
553	Spartina alterniflora	WOA	34.29N	77.25W	T	H	Marsh	NO	Poaceae	NA	0.01	208
554	Rumex hastatulus	WOA	34.3N	77.52W	T	H	Roadside field	NO	Polygonaceae	NA	1.61	208
555	Borrichia frutescens	WOA	34.3N	77.54W	T	S	Marsh	NO	Asteraceae	NA	0.74	208
556	Oenothera humifusa	WOA	34.3N	77.54W	T	H	Dune	NO	Onagraceae	NA	1.62	208
557	Solidago sempervirens	WOA	34.3N	77.54W	T	H	Marsh	NO	Asteraceae	NA	1.07	208

558	Spartina alterniflora	WOA	34.3N	77.54W	T	H	Marsh	NO	Poaceae	NA	0.05	208
559	Decodon verticillatus	WOA	34.32N	78.47W	T	S	Wetland	NO	Lythraceae	NA	1.91	208
560	Oenothera laciniata	WOA	34.36N	78.37W	T	H	Roadside field	NO	Onagraceae	NA	0.90	208
561	Phlox drummondii	WOA	34.36N	78.37W	T	H	Roadside field	NO	Polemoniaceae	NA	0.07	208
562	Rumex hastatulus	WOA	34.36N	78.37W	T	H	Roadside field	NO	Polygonaceae	NA	2.03	208
563	Decodon verticillatus	WOA	34.39N	78.35W	T	S	Wetland	NO	Lythraceae	NA	0.50	208
564	Oenothera humifusa	WOA	34.41N	76.31W	T	H	Dune	NO	Onagraceae	NA	0.46	208
565	Solidago sempervirens	WOA	34.41N	76.31W	T	H	Marsh	NO	Asteraceae	NA	1.00	208
566	Solidago sempervirens	WOA	34.43N	76.33W	T	H	Marsh	NO	Asteraceae	NA	0.18	208
567	Borrichia frutescens	WOA	34.43N	76.34W	T	S	Marsh	NO	Asteraceae	NA	0.88	208
568	Spartina alterniflora	WOA	34.43N	76.34W	T	H	Marsh	NO	Poaceae	NA	0.00	208
569	Solidago sempervirens	WOA	34.43N	76.4W	T	H	Marsh	NO	Asteraceae	NA	2.12	208
570	Borrichia frutescens	WOA	34.43N	76.41W	T	S	Marsh	NO	Asteraceae	NA	0.49	208
571	Oenothera humifusa	WOA	34.47N	76.37W	T	H	Dune	NO	Onagraceae	NA	0.15	208
572	Borrichia frutescens	WOA	34.49N	76.27W	T	S	Marsh	NO	Asteraceae	NA	0.04	208
573	Solidago sempervirens	WOA	34.49N	76.27W	T	H	Marsh	NO	Asteraceae	NA	1.15	208
574	Salix eriocarpa	WOA	35N	136E	T	T	Forest	NO	Salicaceae	Insect	10.76	138
575	Salix gilgiana	WOA	35N	136E	T	T	Forest	NO	Salicaceae	Insect	10.85	138
576	Quercus rubra	WOA	35.03N	83.25W	T	T	Forest	NO	Fagaceae	Insect	25.94	204
577	Acer rubrum	WOA	35.12N	83.2W	T	T	Forest	NO	Sapindaceae	Insect	4.62	179
578	Cornus florida	WOA	35.12N	83.25W	T	T	Forest	NO	Cornaceae	Insect	2.32	179
579	Liriodendron tulipifera	WOA	35.12N	83.25W	T	T	Forest	NO	Magnoliaceae	Insect	2.55	179
580	Fagus crenata	WOA	35.18N	135.43E	T	T	Forest	NO	Fagaceae	Insect	1.93	112
581	Sabatia angularis	WOA	35.21N	79.49W	T	H	Roadside field	NO	Gentianaceae	NA	0.00	208
582	Lathyrus latifolius	WOA	35.26N	81.53W	T	V	Roadside field	NO	Fabaceae	NA	2.42	208
583	Hypericum perforatum	WOA	35.28N	78.55W	T	H	River bank	NO	Hypericaceae	NA	5.25	208
584	Datura stramonium	WOA	35.4N	78W	T	H	Roadside field	NO	Solanaceae	NA	0.24	208

585	Ipomoea hederacea	WOA	35.4N	78W	T	H	Field	NO	Convolvulaceae	NA	12.49	208
586	Ipomoea purpurea	WOA	35.4N	78W	T	H	Field	NO	Convolvulaceae	NA	6.13	208
587	Ipomoea purpurea	WOA	35.4N	78W	T	H	Roadside field	NO	Convolvulaceae	NA	0.87	208
588	Solanum carolinense	WOA	35.4N	78W	T	H	Field	NO	Solanaceae	NA	3.52	208
589	Solanum carolinense	WOA	35.4N	78W	T	H	Pond edge	NO	Solanaceae	NA	1.17	208
590	Asclepias syriaca	WOA	35.43N	78.41W	T	H	Roadside field	NO	Apocynaceae	NA	1.82	208
591	Impatiens capensis	WOA	35.43N	78.41W	T	H	Wetland	NO	Balsaminaceae	NA	3.04	208
592	Liriodendron tulipifera	WOA	35.43N	78.41W	T	T	Forest	NO	Magnoliaceae	NA	3.07	208
593	Lobelia cardinalis	WOA	35.43N	78.41W	T	H	Pond edge	NO	Campanulaceae	NA	0.40	208
594	Rhus glabra	WOA	35.43N	78.41W	T	S	Field	NO	Anacardiaceae	NA	1.03	208
595	Taraxacum officinale	WOA	35.43N	78.41W	T	H	Field	NO	Asteraceae	NA	2.57	208
596	Woodwardia areolata	WOA	35.43N	78.41W	T	H	Marsh	NO	Blechnaceae	NA	1.70	208
597	Lobelia cardinalis	WOA	35.43N	78.45W	T	H	River edge	NO	Campanulaceae	NA	2.50	208
598	Capsella bursa-pastoris	WOA	35.44N	78.42W	T	H	Forest	NO	Brassicaceae	NA	0.10	208
599	Erigeron annuus	WOA	35.44N	78.42W	T	H	Field	NO	Asteraceae	NA	2.46	208
600	Liriodendron tulipifera	WOA	35.44N	78.42W	T	T	Forest	NO	Magnoliaceae	NA	4.76	208
601	Lolium multiflorum	WOA	35.44N	78.42W	T	H	Roadside field	NO	Poaceae	NA	0.05	208
602	Oenothera laciniata	WOA	35.44N	78.42W	T	H	Field	NO	Onagraceae	NA	0.18	208
603	Rhus glabra	WOA	35.44N	78.42W	T	S	Field	NO	Anacardiaceae	NA	6.38	208
604	Taraxacum officinale	WOA	35.44N	78.42W	T	H	Roadside field	NO	Asteraceae	NA	3.68	208
605	Acer rubrum	WOA	35.45N	79.1W	T	T	Forest	NO	Sapindaceae	Insect	5.11	161
606	Quercus rubra	WOA	35.45N	79.19W	T	T	Forest	NO	Fagaceae	Insect	5.53	161
607	Oenothera biennis	WOA	35.46N	78.39W	T	H	Field	NO	Onagraceae	NA	6.79	208
608	Senecio vulgaris	WOA	35.46N	78.39W	T	H	Unclear	NO	Asteraceae	NA	2.44	208
609	Ambrosia artemisiifolia	WOA	35.46N	78.4W	T	H	Field	NO	Asteraceae	NA	2.07	208
610	Ambrosia artemisiifolia	WOA	35.46N	78.4W	T	H	Urban field	NO	Asteraceae	NA	0.69	208
611	Impatiens capensis	WOA	35.46N	78.4W	T	H	Field	NO	Balsaminaceae	NA	1.13	208
612	Liriodendron tulipifera	WOA	35.46N	78.4W	T	T	Forest	NO	Magnoliaceae	NA	3.16	208
613	Oenothera biennis	WOA	35.46N	78.4W	T	H	Field	NO	Onagraceae	NA	5.75	208

614	Taraxacum officinale	WOA	35.46N	78.4W	T	H	Field	NO	Asteraceae	NA	0.24	208
615	Rhus glabra	WOA	35.47N	78.39W	T	S	Urban field	NO	Anacardiaceae	NA	4.00	208
616	Erigeron annuus	WOA	35.47N	78.4W	T	H	Field	NO	Asteraceae	NA	0.60	208
617	Erigeron annuus	WOA	35.47N	78.4W	T	H	Roadside field	NO	Asteraceae	NA	2.74	208
618	Lolium multiflorum	WOA	35.47N	78.4W	T	H	Roadside field	NO	Poaceae	NA	0.05	208
619	Oenothera laciniata	WOA	35.47N	78.4W	T	H	Field	NO	Onagraceae	NA	0.69	208
620	Taraxacum officinale	WOA	35.47N	78.4W	T	H	Roadside field	NO	Asteraceae	NA	0.22	208
621	Ambrosia artemisiifolia	WOA	35.47N	78.41W	T	H	Field	NO	Asteraceae	NA	0.68	208
622	Lolium multiflorum	WOA	35.47N	78.41W	T	H	Grassland	NO	Poaceae	NA	0.01	208
623	Rumex acetosella	WOA	35.47N	78.41W	T	H	Field	NO	Polygonaceae	NA	5.56	208
624	Taraxacum officinale	WOA	35.48N	78.39W	T	H	Roadside field	NO	Asteraceae	NA	1.53	208
625	Asclepias syriaca	WOA	35.48N	78.41W	T	H	Grassland	NO	Apocynaceae	NA	1.68	208
626	Impatiens capensis	WOA	35.48N	78.41W	T	H	Field	NO	Balsaminaceae	NA	2.44	208
627	Ambrosia artemisiifolia	WOA	35.48N	78.42W	T	H	Field	NO	Asteraceae	NA	1.14	208
628	Carduus nutans	WOA	35.48N	79.5W	T	H	Roadside field	NO	Asteraceae	NA	4.83	208
629	Sabatia angularis	WOA	35.5N	80.8W	T	H	Roadside field	NO	Gentianaceae	NA	9.40	208
630	Hypericum perforatum	WOA	35.52N	78.5W	T	H	Field	NO	Hypericaceae	NA	0.01	208
631	Juglans nigra	WOA	35.54N	80.52W	T	T	Unclear	NO	Juglandaceae	NA	8.75	208
632	Acer negundo	WOA	35.54N	84.2W	T	T	Forest	NO	Sapindaceae	Insect	41.00	170
633	Lonicera japonica	WOA	35.54N	84.2W	T	V	Forest	NO	Caprifoliaceae	Insect	8.00	170
634	Microstegium vimineum	WOA	35.54N	84.2W	T	H	Forest	NO	Poaceae	Insect	2.50	170
635	Rubus spp.	WOA	35.54N	84.2W	T	S	Forest	NO	Rosaceae	Insect	32.00	170
636	Solidago canadensis	WOA	35.54N	84.2W	T	H	Forest	NO	Asteraceae	Insect	46.00	170
637	Liriodendron tulipifera	WOA	35.55N	84.17W	T	T	Forest	NO	Magnoliaceae	Insect	2.51	160
638	Hordeum vulgare	WOA	35.57N	78.59W	T	H	Disturbed soil	NO	Poaceae	NA	0.00	208

639	Lolium multiflorum	WOA	35.57N	78.59W	T	H	Disturbed soil	NO	Poaceae	NA	0.03	208
640	Acer rubrum	WOA	35.58N	79W	T	T	Forest	NO	Sapindaceae	Insect	9.31	78
641	Liquidambar styraciflua	WOA	35.58N	79W	T	T	Forest	NO	Altingiaceae	Insect	3.95	78
642	Cercis canadensis	WOA	35.58N	79.05W	T	T	Forest	NO	Fabaceae	Insect	3.74	78
643	Ulmus alata	WOA	35.58N	79.05W	T	T	Forest	NO	Ulmaceae	Insect	6.11	78
644	Cercis canadensis	WOA	35.58N	79.09W	T	T	Forest	NO	Fabaceae	Insect	6.16	98
645	Liriodendron tulipifera	WOA	35.58N	79.09W	T	T	Forest	NO	Magnoliaceae	Insect	3.08	98
646	Quercus phellos	WOA	35.58N	79.09W	T	T	Forest	NO	Fagaceae	Insect	4.53	98
647	Quercus rubra	WOA	35.58N	79.09W	T	T	Forest	NO	Fagaceae	Insect	4.26	98
648	Quercus velutina	WOA	35.58N	79.09W	T	T	Forest	NO	Fagaceae	Insect	3.21	98
649	Ulmus alata	WOA	35.58N	79.09W	T	T	Forest	NO	Ulmaceae	Insect	2.49	98
650	Sabatia angularis	WOA	35.58N	79.53W	T	H	Roadside field	NO	Gentianaceae	NA	0.50	208
651	Lathyrus latifolius	WOA	35.58N	80.5W	T	V	Roadside field	NO	Fabaceae	NA	0.13	208
652	Lolium multiflorum	WOA	35.59N	78.58W	T	H	Disturbed soil	NO	Poaceae	NA	0.28	208
653	Oenothera laciniata	WOA	35.59N	78.58W	T	H	Disturbed soil	NO	Onagraceae	NA	0.49	208
654	Acer rubrum	WOA	35.97N	79W	T	T	Forest	NO	Sapindaceae	Insect	6.62	98
655	Acer saccharum	WOA	35.97N	79W	T	T	Forest	NO	Sapindaceae	Insect	2.43	98
656	Liquidambar styraciflua	WOA	35.97N	79W	T	T	Forest	NO	Altingiaceae	Insect	1.44	98
657	Arabidopsis thaliana	WOA	36N	78.5W	T	H	Field	NO	Brassicaceae	Insect	9.83	125
658	Lobelia cardinalis	WOA	36.1N	78.55W	T	H	Wetland	NO	Campanulaceae	NA	0.50	208
659	Lathyrus latifolius	WOA	36.13N	81.36W	T	V	Roadside field	NO	Fabaceae	NA	0.70	208
660	Lathyrus latifolius	WOA	36.18N	81.41W	T	V	Roadside field	NO	Fabaceae	NA	0.54	208
661	Sabatia angularis	WOA	36.19N	79.14W	T	H	Roadside field	NO	Gentianaceae	NA	5.76	208
662	Rhus typhina	WOA	36.21N	81.18W	T	S	Field	NO	Anacardiaceae	NA	0.75	208

663	Sabatia angularis	WOA	36.24N	80.57W	T	H	Roadside field	NO	Gentianaceae	NA	8.04	208
664	Ipomoea purpurea	WOA	36.28N	79.2W	T	H	Field	NO	Convolvulaceae	NA	2.65	208
665	Asclepias syriaca	WOA	36.28N	79.21W	T	H	Field	NO	Apocynaceae	NA	3.85	208
666	Asclepias syriaca	WOA	36.28N	79.21W	T	H	Grassland	NO	Apocynaceae	NA	23.40	208
667	Erigeron annuus	WOA	36.28N	79.21W	T	H	Field	NO	Asteraceae	NA	5.24	208
668	Liriodendron tulipifera	WOA	36.28N	79.21W	T	T	Forest	NO	Magnoliaceae	NA	2.87	208
669	Lathyrus latifolius	WOA	36.28N	79.23W	T	V	Roadside field	NO	Fabaceae	NA	0.76	208
670	Rhus glabra	WOA	36.28N	79.23W	T	S	Field	NO	Anacardiaceae	NA	4.75	208
671	Rumex acetosella	WOA	36.28N	79.23W	T	H	Roadside field	NO	Polygonaceae	NA	1.85	208
672	Impatiens capensis	WOA	36.3N	79.22W	T	H	Forest	NO	Balsaminaceae	NA	1.24	208
673	Asclepias syriaca	WOA	36.31N	79.23W	T	H	Grassland	NO	Apocynaceae	NA	1.45	208
674	Trillium erectum	WOA	36.33N	80.54W	T	H	Forest	NO	Melanthiaceae	NA	7.00	208
675	Impatiens capensis	WOA	36.4N	78.54W	T	H	Pond edge	NO	Balsaminaceae	NA	1.15	208
676	Juglans nigra	WOA	36.4N	78.54W	T	T	Forest	NO	Juglandaceae	NA	6.81	208
677	Kalmia latifolia	WOA	36.4N	78.54W	T	S	Forest	NO	Ericaceae	NA	3.07	208
678	Polystichum acrostichoides	WOA	36.4N	78.54W	T	H	Forest	NO	Dryopteridaceae	NA	2.31	208
679	Posidonia oceanica	WOA	36.41N	2.363W	A	H	Coast	NO	Posidoniaceae	Others	0.90	33
680	Posidonia oceanica	WOA	36.44N	2.345W	A	H	Coast	NO	Posidoniaceae	Others	1.40	33
681	Posidonia oceanica	WOA	36.45N	2.324W	A	H	Coast	NO	Posidoniaceae	Others	0.90	33
682	Sabatia angularis	WOA	36.52N	81.11W	T	H	Roadside field	NO	Gentianaceae	NA	0.78	208
683	Posidonia oceanica	WOA	36.56N	1.523W	A	H	Coast	NO	Posidoniaceae	Others	1.60	33
684	Hypericum perforatum	WOA	36.8N	79.26W	T	H	Field	NO	Hypericaceae	NA	3.50	208
685	Hypericum perforatum	WOA	36.9N	81.19W	T	H	Field	NO	Hypericaceae	NA	1.21	208
686	Trillium erectum	WOA	36.9N	81.19W	T	H	Forest	NO	Melanthiaceae	NA	3.47	208
687	Asclepias syriaca	WOA	37.15N	80.36W	T	H	Field	NO	Apocynaceae	NA	0.84	208
688	Aquilegia canadensis	WOA	37.18N	80.3W	T	H	Forest	NO	Ranunculaceae	NA	1.43	208
689	Oenothera biennis	WOA	37.19N	80.32W	T	H	Unclear	NO	Onagraceae	NA	6.00	208
690	Solanum ptychanthum	WOA	37.2N	80W	T	H	Field	NO	Solanaceae	NA	8.86	208
691	Campanula americanum	WOA	37.2N	80.32W	C	H	Forest	NO	Campanulaceae	Insect	12.70	203

692	Campanula americanum	WOA	37.2N	80.32W	T	H	Field	NO	Campanulaceae	NA	0.83	208
693	Asclepias syriaca	WOA	37.2N	80.33W	T	H	Grassland	NO	Apocynaceae	NA	0.50	208
694	Asclepias syriaca	WOA	37.21N	80.32W	T	H	Grassland	NO	Apocynaceae	NA	0.10	208
695	Asclepias syriaca	WOA	37.21N	80.32W	T	H	Unclear	NO	Apocynaceae	NA	0.36	208
696	Kalmia latifolia	WOA	37.21N	80.32W	T	S	Unclear	NO	Ericaceae	NA	1.50	208
697	Rumex acetosella	WOA	37.21N	80.32W	T	H	Unclear	NO	Polygonaceae	NA	0.86	208
698	Asclepias syriaca	WOA	37.21N	80.33W	T	H	Forest	NO	Apocynaceae	NA	3.13	208
699	Rumex acetosella	WOA	37.21N	80.33W	T	H	Field	NO	Polygonaceae	NA	4.66	208
700	Campanula americanum	WOA	37.21N	80.36W	T	H	Forest	NO	Campanulaceae	NA	0.62	208
701	Trillium grandiflorum	WOA	37.21N	80.36W	T	H	Forest	NO	Melanthiaceae	NA	0.00	208
702	Posidonia oceanica	WOA	37.22N	1.331W	A	H	Coast	NO	Posidoniaceae	Others	0.90	33
703	Acer pensylvanicum	WOA	37.22N	80.3W	C	T	Forest	NO	Sapindaceae	Insect	3.27	120
704	Asclepias incarnata	WOA	37.22N	80.31W	T	H	Field	NO	Apocynaceae	NA	0.00	208
705	Asclepias syriaca	WOA	37.22N	80.31W	T	H	Field	NO	Apocynaceae	NA	8.15	208
706	Asclepias syriaca	WOA	37.22N	80.31W	T	H	Grassland	NO	Apocynaceae	NA	0.52	208
707	Erigeron annuus	WOA	37.22N	80.31W	T	H	Field	NO	Asteraceae	NA	2.68	208
708	Hypericum perforatum	WOA	37.22N	80.31W	T	H	Field	NO	Hypericaceae	NA	4.10	208
709	Lolium multiflorum	WOA	37.22N	80.31W	T	H	Field	NO	Poaceae	NA	0.00	208
710	Oenothera biennis	WOA	37.22N	80.31W	T	H	Unclear	NO	Onagraceae	NA	4.24	208
711	Picea rubens	WOA	37.22N	80.31W	T	T	Forest	NO	Pinaceae	NA	0.00	208
712	Asclepias syriaca	WOA	37.23N	80.3W	T	H	Grassland	NO	Apocynaceae	NA	0.00	208
713	Kalmia latifolia	WOA	37.23N	80.3W	T	S	Forest	NO	Ericaceae	NA	0.86	208
714	Posidonia oceanica	WOA	37.25N	1.485W	A	H	Coast	NO	Posidoniaceae	Others	0.30	33
715	Asclepias syriaca	WOA	37.25N	80.3W	T	H	Grassland	NO	Apocynaceae	NA	0.32	208
716	Asclepias syriaca	WOA	37.26N	80.3W	T	H	Field	NO	Apocynaceae	NA	0.24	208
717	Asclepias incarnata	WOA	37.26N	80.31W	T	H	Field	NO	Apocynaceae	NA	0.30	208
718	Asclepias syriaca	WOA	37.26N	80.31W	T	H	Field	NO	Apocynaceae	NA	0.82	208
719	Mimulus ringens	WOA	37.26N	80.31W	T	H	Field	NO	Phrymaceae	NA	1.55	208
720	Posidonia oceanica	WOA	37.29N	1.012W	A	H	Coast	NO	Posidoniaceae	Others	0.80	33
721	Posidonia oceanica	WOA	37.3N	0.552W	A	H	Coast	NO	Posidoniaceae	Others	1.70	33
722	Posidonia oceanica	WOA	37.33N	0.463W	A	H	Coast	NO	Posidoniaceae	Others	1.30	33
723	Posidonia oceanica	WOA	37.33N	0.492W	A	H	Coast	NO	Posidoniaceae	Others	1.70	33
724	Daphne laureola	WOA	37.54N	3.013W	A	S	Forest	NO	Thymelaeaceae	Insect	4.30	10

725	Quercus rubra	WOA	37.56N	90.47W	C	T	Forest	NO	Fagaceae	Insect	20.45	110
726	Posidonia oceanica	WOA	38.1N	0.294W	A	H	Coast	NO	Posidoniaceae	Others	1.80	33
727	Baccharis pilularis	WOA	38.19N	123.04W	T	S	Dune	NO	Asteraceae	Insect	6.28	168
728	Lupinus nanus	WOA	38.19N	123.04W	T	S	Grassland	NO	Fabaceae	Insect	19.69	167
729	Posidonia oceanica	WOA	38.33N	0.246E	A	H	Coast	NO	Posidoniaceae	Others	2.00	33
730	Ulmus spp.	WOA	38.33N	121.3W	T	T	Urban field	NO	Ulmaceae	Insect	22.00	108
731	Posidonia oceanica	WOA	38.34N	0.758E	A	H	Coast	NO	Posidoniaceae	Others	1.50	33
732	Posidonia oceanica	WOA	38.37N	0.133E	A	H	Coast	NO	Posidoniaceae	Others	2.50	33
733	Posidonia oceanica	WOA	38.4N	0.141E	A	H	Coast	NO	Posidoniaceae	Others	1.50	33
734	Raphanus raphanistrum	WOA	38.51N	120.36W	T	H	Fallow field	NO	Brassicaceae	Insect	12.21	4
735	Raphanus sativus	WOA	38.51N	120.36W	T	H	Fallow field	NO	Brassicaceae	Insect	39.80	4
736	Agastache urticifolia	WOA	38.57N	106.59W	C	S	Shrubland	NO	Lamiaceae	NA	7.60	140
737	Alnus incana	WOA	38.57N	106.59W	C	T	Shrubland	NO	Betulaceae	NA	2.90	140
738	Aquilegia coerulea	WOA	38.57N	106.59W	C	H	Shrubland	NO	Ranunculaceae	Insect	8.05	115
739	Bromus inermis	WOA	38.57N	106.59W	C	H	Shrubland	NO	Poaceae	NA	5.60	140
740	Campanula rotundifolia	WOA	38.57N	106.59W	C	H	Shrubland	NO	Campanulaceae	NA	1.20	140
741	Castilleja sulphurea	WOA	38.57N	106.59W	C	S	Shrubland	NO	Orobanchaceae	NA	8.50	140
742	Chamerion angustifolium	WOA	38.57N	106.59W	C	H	Shrubland	NO	Onagraceae	NA	21.90	140
743	Cirsium parryi	WOA	38.57N	106.59W	C	H	Shrubland	NO	Asteraceae	NA	16.70	140
744	Delphinium barbeyi	WOA	38.57N	106.59W	C	H	Shrubland	NO	Ranunculaceae	NA	6.70	140
745	Delphinium nelsonii	WOA	38.57N	106.59W	C	H	Shrubland	NO	Ranunculaceae	Insect	16.20	115
746	Elymus glaucus	WOA	38.57N	106.59W	C	H	Shrubland	NO	Poaceae	NA	16.50	140
747	Erigeron speciosus	WOA	38.57N	106.59W	C	H	Shrubland	NO	Asteraceae	Insect	3.95	115
748	Eriogonum umbellatum	WOA	38.57N	106.59W	C	S	Shrubland	NO	Polygonaceae	NA	0.60	140
749	Fragaria ovalis	WOA	38.57N	106.59W	C	H	Shrubland	NO	Rosaceae	Insect	9.85	115
750	Fragaria virginiana	WOA	38.57N	106.59W	C	H	Shrubland	NO	Rosaceae	NA	3.30	140
751	Galium biflorum	WOA	38.57N	106.59W	C	H	Shrubland	NO	Rubiaceae	Insect	2.40	115
752	Galium boreale	WOA	38.57N	106.59W	C	H	Shrubland	NO	Rubiaceae	NA	6.80	140
753	Gentiana parryi	WOA	38.57N	106.59W	C	H	Shrubland	NO	Gentianaceae	NA	1.80	140
754	Geranium richardsonii	WOA	38.57N	106.59W	C	H	Shrubland	NO	Geraniaceae	NA	3.40	140
755	Ipomopsis aggregata	WOA	38.57N	106.59W	C	H	Shrubland	NO	Polemoniaceae	NA	2.50	140
756	Iris missouriensis	WOA	38.57N	106.59W	C	H	Shrubland	NO	Iridaceae	NA	1.20	140
757	Juncus mertensianus	WOA	38.57N	106.59W	C	H	Shrubland	NO	Juncaceae	NA	7.50	140

758	Lathyrus leucanthus	WOA	38.57N	106.59W	C	H	Shrubland	NO	Fabaceae	Insect	5.75	115
759	Linaria vulgaris	WOA	38.57N	106.59W	C	H	Shrubland	NO	Plantaginaceae	NA	1.10	140
760	Linum lewisii	WOA	38.57N	106.59W	C	S	Shrubland	NO	Linaceae	NA	6.90	140
761	Lonicera involucrata	WOA	38.57N	106.59W	C	S	Shrubland	NO	Caprifoliaceae	NA	2.90	140
762	Lupinus argenteus	WOA	38.57N	106.59W	C	H	Shrubland	NO	Fabaceae	Insect	5.95	115
763	Lupinus argenteus	WOA	38.57N	106.59W	C	H	Shrubland	NO	Fabaceae	NA	5.70	140
764	Mahonia repens	WOA	38.57N	106.59W	C	S	Shrubland	NO	Berberidaceae	NA	9.00	140
765	Maianthemum stellatum	WOA	38.57N	106.59W	C	H	Shrubland	NO	Asparagaceae	NA	1.40	140
766	Paxistima myrsinites	WOA	38.57N	106.59W	C	S	Shrubland	NO	Celastraceae	NA	0.20	140
767	Phleum pratense	WOA	38.57N	106.59W	C	H	Shrubland	NO	Poaceae	NA	1.20	140
768	Polemonium foliosissimum	WOA	38.57N	106.59W	C	H	Shrubland	NO	Polemoniaceae	NA	3.60	140
769	Rumex densiflorus	WOA	38.57N	106.59W	C	H	Shrubland	NO	Polygonaceae	NA	40.50	140
770	Shepherdia canadensis	WOA	38.57N	106.59W	C	S	Shrubland	NO	Elaeagnaceae	NA	1.80	140
771	Silene latifolia	WOA	38.57N	106.59W	C	H	Shrubland	NO	Caryophyllaceae	NA	5.90	140
772	Thalictrum fendleri	WOA	38.57N	106.59W	C	H	Shrubland	NO	Ranunculaceae	Insect	9.55	115
773	Thlaspi arvense	WOA	38.57N	106.59W	C	H	Shrubland	NO	Brassicaceae	NA	1.20	140
774	Tragopogon pratensis	WOA	38.57N	106.59W	C	H	Shrubland	NO	Asteraceae	NA	3.90	140
775	Trifolium repens	WOA	38.57N	106.59W	C	H	Shrubland	NO	Fabaceae	NA	6.00	140
776	Valeriana edulis	WOA	38.57N	106.59W	C	H	Shrubland	NO	Caprifoliaceae	NA	10.70	140
777	Vicia americana	WOA	38.57N	106.59W	C	H	Shrubland	NO	Fabaceae	Insect	5.55	115
778	Viguiera multiflora	WOA	38.57N	106.59W	C	H	Shrubland	NO	Asteraceae	Insect	20.85	115
779	Viguiera multiflora	WOA	38.57N	106.59W	C	H	Shrubland	NO	Asteraceae	NA	12.50	140
780	Viola nuttallii	WOA	38.57N	106.59W	C	H	Shrubland	NO	Violaceae	Insect	8.15	115
781	Posidonia oceanica	WOA	38.74N	0.333W	A	H	Coast	NO	Posidoniaceae	Others	1.90	33
782	Posidonia oceanica	WOA	38.91N	0.315W	A	H	Coast	NO	Posidoniaceae	Others	1.00	33
783	Andropogon bladhii	WOA	39.05N	96.35W	A	H	Grassland	NO	Poaceae	Insect	6.70	80
784	Andropogon gerardii	WOA	39.05N	96.35W	A	H	Grassland	NO	Poaceae	Insect	16.24	80
785	Bromus inermis	WOA	39.05N	96.35W	A	H	Grassland	NO	Poaceae	Insect	2.84	80
786	Coronilla varia	WOA	39.05N	96.35W	A	H	Grassland	NO	Fabaceae	Insect	7.92	80
787	Elymus canadensis	WOA	39.05N	96.35W	A	H	Grassland	NO	Poaceae	Insect	5.18	80
788	Lactuca ludoviciana	WOA	39.05N	96.35W	A	H	Grassland	NO	Asteraceae	Insect	12.89	80
789	Lactuca serriola	WOA	39.05N	96.35W	A	H	Grassland	NO	Asteraceae	Insect	5.38	80
790	Melilotus officinalis	WOA	39.05N	96.35W	A	H	Grassland	NO	Fabaceae	Insect	9.85	80

791	Psoralea tenuiflora	WOA	39.05N	96.35W	A	H	Grassland	NO	Fabaceae	Insect	11.42	80
792	Tragopogon dubius	WOA	39.05N	96.35W	A	H	Grassland	NO	Asteraceae	Insect	5.69	80
793	Glycine max	WOA	39.31N	84.43W	C	H	Agricultural	NO	Fabaceae	Insect	34.35	169
794	Solanum carolinense	WOA	39.54N	75.4W	C	H	Grassland	NO	Solanaceae	NA	37.15	30
795	Apocynum cannabinum	WOA	39.54N	75.47W	C	H	Grassland	NO	Apocynaceae	NA	12.12	30
796	Linaria vulgaris	WOA	39.54N	75.47W	C	H	Grassland	NO	Plantaginaceae	NA	3.34	30
797	Poa pratensis	WOA	39.54N	75.47W	C	H	Grassland	NO	Poaceae	NA	3.39	30
798	Potentilla recta	WOA	39.54N	75.47W	C	H	Grassland	NO	Rosaceae	NA	51.50	30
799	Trillium grandiflorum	WOA	41.2N	79.58W	C	H	Forest	NO	Melanthiaceae	Others	20.86	100
800	Trillium grandiflorum	WOA	41.24N	80W	C	H	Forest	NO	Melanthiaceae	Others	17.47	100
801	Trillium grandiflorum	WOA	41.24N	80.12W	C	H	Forest	NO	Melanthiaceae	Others	24.85	100
802	Trillium grandiflorum	WOA	41.29N	80.24W	C	H	Forest	NO	Melanthiaceae	Others	12.59	100
803	Trillium grandiflorum	WOA	41.31N	80.26W	C	H	Forest	NO	Melanthiaceae	Others	0.22	100
804	Trillium grandiflorum	WOA	41.36N	80.21W	C	H	Forest	NO	Melanthiaceae	Others	33.98	100
805	Trillium grandiflorum	WOA	41.4N	80.14W	C	H	Forest	NO	Melanthiaceae	Others	0.00	100
806	Posidonia oceanica	WOA	41.41N	2.555E	T	H	Coast	NO	Posidoniaceae	Others	1.90	33
807	Trillium grandiflorum	WOA	41.41N	80.04W	C	H	Forest	NO	Melanthiaceae	Others	9.51	100
808	Posidonia oceanica	WOA	41.43N	2.594E	T	H	Coast	NO	Posidoniaceae	Others	1.60	33
809	Trillium grandiflorum	WOA	41.43N	80.02W	C	H	Forest	NO	Melanthiaceae	Others	3.85	100
810	Trillium grandiflorum	WOA	41.44N	80.03W	C	H	Forest	NO	Melanthiaceae	Others	6.03	100
811	Trillium grandiflorum	WOA	41.45N	79.57W	C	H	Forest	NO	Melanthiaceae	Others	31.67	100
812	Posidonia oceanica	WOA	41.46N	3.35E	T	H	Coast	NO	Posidoniaceae	Others	2.30	33
813	Trillium grandiflorum	WOA	41.47N	80.05W	C	H	Forest	NO	Melanthiaceae	Others	22.14	100
814	Posidonia oceanica	WOA	42.1N	3.125E	T	H	Coast	NO	Posidoniaceae	Others	1.70	33
815	Posidonia oceanica	WOA	42.15N	3.178E	T	H	Coast	NO	Posidoniaceae	Others	2.30	33
816	Posidonia oceanica	WOA	42.2N	3.195E	T	H	Coast	NO	Posidoniaceae	Others	1.90	33
817	Posidonia oceanica	WOA	42.21N	3.213E	T	H	Coast	NO	Posidoniaceae	Others	4.40	33
818	Brasenia schreberi	WOA	42.23N	84.01W	C	H	Unclear	NO	Nymphaeaceae	NA	2.43	207
819	Brassica oleracea	WOA	42.25N	8.39W	T	H	Agricultural	NO	Brassicaceae	Insect	31.50	152
820	Ambrosia artemisiifolia	WOA	42.25N	76.31W	C	H	Fallow field	NO	Asteraceae	Insect	1.40	32
821	Chrysanthemum leucanthemum	WOA	42.25N	76.31W	C	H	Fallow field	NO	Asteraceae	Insect	1.15	32
822	Erigeron strigosus	WOA	42.25N	76.31W	C	H	Fallow field	NO	Asteraceae	Insect	1.20	32
823	Euthamia graminifolia	WOA	42.25N	76.31W	C	H	Fallow field	NO	Asteraceae	Insect	1.90	32

824	Panicum capillare	WOA	42.25N	76.31W	C	H	Fallow field	NO	Poaceae	Insect	1.20	32
825	Panicum lanuginosum	WOA	42.25N	76.31W	C	H	Fallow field	NO	Poaceae	Insect	2.70	32
826	Plantago major	WOA	42.25N	76.31W	C	H	Fallow field	NO	Plantaginaceae	Insect	16.40	32
827	Rumex acetosella	WOA	42.25N	76.31W	C	H	Fallow field	NO	Polygonaceae	Insect	0.90	32
828	Trifolium hybridum	WOA	42.25N	76.31W	C	H	Fallow field	NO	Fabaceae	Insect	1.40	32
829	Asplenium platyneuron	WOA	42.27N	83.5W	C	H	Unclear	NO	Aspleniaceae	NA	2.28	207
830	Nymphaea odorata	WOA	42.27N	83.5W	C	H	Unclear	NO	Nymphaeaceae	NA	1.50	207
831	Larix americana	WOA	42.31N	83.51W	C	T	Unclear	NO	Pinaceae	NA	12.23	207
832	Posidonia oceanica	WOA	42.91N	3.233E	T	H	Coast	NO	Posidoniaceae	Others	3.70	33
833	Aegopodium podagraria	WOA	43.1N	73.5W	C	H	Forest	NO	Apiaceae	NA	14.70	140
834	Alliaria petiolata	WOA	43.1N	73.5W	C	H	Forest	NO	Brassicaceae	NA	2.50	140
835	Asarum canadense	WOA	43.1N	73.5W	C	H	Forest	NO	Aristolochiaceae	NA	10.90	140
836	Asclepias syriaca	WOA	43.1N	73.5W	C	H	Forest	NO	Apocynaceae	NA	24.20	140
837	Aster novae-angliae	WOA	43.1N	73.5W	C	H	Forest	NO	Asteraceae	NA	8.40	140
838	Berberis thunbergii	WOA	43.1N	73.5W	C	S	Forest	NO	Berberidaceae	NA	2.60	140
839	Betula papyrifera	WOA	43.1N	73.5W	C	T	Forest	NO	Betulaceae	NA	10.50	140
840	Carex plantaginea	WOA	43.1N	73.5W	C	H	Forest	NO	Cyperaceae	NA	4.00	140
841	Carya ovata	WOA	43.1N	73.5W	C	T	Forest	NO	Juglandaceae	NA	13.60	140
842	Celastrus orbiculata	WOA	43.1N	73.5W	C	V	Forest	NO	Celastraceae	NA	5.30	140
843	Cichorium intybus	WOA	43.1N	73.5W	C	H	Forest	NO	Asteraceae	NA	22.30	140
844	Cornus alternifolia	WOA	43.1N	73.5W	C	T	Forest	NO	Cornaceae	NA	19.80	140
845	Daucus carota	WOA	43.1N	73.5W	C	H	Forest	NO	Apiaceae	NA	7.80	140
846	Euonymus alatus	WOA	43.1N	73.5W	C	S	Forest	NO	Celastraceae	NA	4.30	140
847	Fagus grandifolia	WOA	43.1N	73.5W	C	T	Forest	NO	Fagaceae	NA	13.00	140
848	Fragaria virginiana	WOA	43.1N	73.5W	C	H	Forest	NO	Rosaceae	NA	10.80	140
849	Hepatica nobilis	WOA	43.1N	73.5W	C	H	Forest	NO	Ranunculaceae	NA	7.60	140
850	Lindera benzoin	WOA	43.1N	73.5W	C	T	Forest	NO	Lauraceae	NA	10.40	140
851	Lonicera morrowi	WOA	43.1N	73.5W	C	S	Forest	NO	Caprifoliaceae	NA	3.10	140
852	Lythrum salicaria	WOA	43.1N	73.5W	C	S	Forest	NO	Lythraceae	NA	10.00	140
853	Phragmites australis	WOA	43.1N	73.5W	C	H	Forest	NO	Poaceae	NA	1.90	140
854	Phytolacca americana	WOA	43.1N	73.5W	C	H	Forest	NO	Phytolaccaceae	NA	1.50	140
855	Plantago major	WOA	43.1N	73.5W	C	H	Forest	NO	Plantaginaceae	NA	13.70	140
856	Polygonum cuspidatum	WOA	43.1N	73.5W	C	S	Forest	NO	Polygonaceae	NA	6.60	140

857	Populus deltoides	WOA	43.1N	73.5W	C	T	Forest	NO	Salicaceae	NA	5.80	140
858	Quercus rubra	WOA	43.1N	73.5W	C	T	Forest	NO	Fagaceae	NA	28.10	140
859	Rhamnus cathartica	WOA	43.1N	73.5W	C	T	Forest	NO	Rhamnaceae	NA	5.60	140
860	Rhus radicans	WOA	43.1N	73.5W	C	V	Forest	NO	Anacardiaceae	NA	2.50	140
861	Rhus typhina	WOA	43.1N	73.5W	C	S	Forest	NO	Anacardiaceae	NA	14.30	140
862	Sanguinaria canadensis	WOA	43.1N	73.5W	C	H	Forest	NO	Papaveraceae	NA	6.40	140
863	Solidago canadensis	WOA	43.1N	73.5W	C	H	Forest	NO	Asteraceae	NA	9.30	140
864	Thalictrum dioicum	WOA	43.1N	73.5W	C	H	Forest	NO	Ranunculaceae	NA	6.90	140
865	Tilia americana	WOA	43.1N	73.5W	C	T	Forest	NO	Malvaceae	NA	5.20	140
866	Trifolium pratense	WOA	43.1N	73.5W	C	H	Forest	NO	Fabaceae	NA	17.70	140
867	Trillium erectum	WOA	43.1N	73.5W	C	H	Forest	NO	Melanthiaceae	NA	6.10	140
868	Tussilago farfara	WOA	43.1N	73.5W	C	H	Forest	NO	Asteraceae	NA	16.60	140
869	Verbascum thapsus	WOA	43.1N	73.5W	C	H	Forest	NO	Scrophulariaceae	NA	2.40	140
870	Viburnum acerifolium	WOA	43.1N	73.5W	C	S	Forest	NO	Adoxaceae	NA	12.60	140
871	Salix miyabeana	WOA	43.11N	141.24E	C	T	Forest	NO	Salicaceae	Insect	11.48	105
872	Fagus grandifolia	WOA	43.39N	80.26W	C	T	Forest	NO	Fagaceae	Insect	7.13	24
873	Fraxinus pennsylvanica	WOA	43.39N	80.26W	C	T	Forest	NO	Oleaceae	Insect	5.00	24
874	Quercus velutina	WOA	43.39N	80.26W	C	T	Forest	NO	Fagaceae	Insect	8.50	24
875	Acer saccharum	WOA	43.56N	71.4W	C	T	Forest	NO	Sapindaceae	Insect	12.46	189
876	Ambrosia artemisiifolia	WOA	44.03N	79.29W	C	H	Fallow field	NO	Asteraceae	Insect	3.27	117
877	Artemisia biennis	WOA	44.03N	79.29W	C	H	Fallow field	NO	Asteraceae	Insect	3.85	5
878	Artemisia biennis	WOA	44.03N	79.29W	C	H	Fallow field	NO	Asteraceae	Insect	4.49	6
879	Artemisia campestris	WOA	44.03N	79.29W	C	H	Fallow field	NO	Asteraceae	Insect	1.00	5
880	Artemisia campestris	WOA	44.03N	79.29W	C	H	Fallow field	NO	Asteraceae	Insect	11.06	6
881	Asclepias syriaca	WOA	44.03N	79.29W	C	H	Fallow field	NO	Apocynaceae	Insect	2.97	5
882	Asclepias syriaca	WOA	44.03N	79.29W	C	H	Fallow field	NO	Apocynaceae	Insect	7.92	6
883	Bromus inermis	WOA	44.03N	79.29W	C	H	Fallow field	NO	Poaceae	Insect	1.40	5
884	Bromus inermis	WOA	44.03N	79.29W	C	H	Fallow field	NO	Poaceae	Insect	0.52	6
885	Bromus kalmii	WOA	44.03N	79.29W	C	H	Fallow field	NO	Poaceae	Insect	1.48	5
886	Bromus kalmii	WOA	44.03N	79.29W	C	H	Fallow field	NO	Poaceae	Insect	0.26	6
887	Campanula rapunculoides	WOA	44.03N	79.29W	C	H	Fallow field	NO	Campanulaceae	Insect	7.54	5
888	Campanula rapunculoides	WOA	44.03N	79.29W	C	H	Fallow field	NO	Campanulaceae	Insect	0.45	6
889	Campanula rotundifolia	WOA	44.03N	79.29W	C	H	Fallow field	NO	Campanulaceae	Insect	0.72	5

890	Campanula rotundifolia	WOA	44.03N	79.29W	C	H	Fallow field	NO	Campanulaceae	Insect	0.45	6
891	Cerastium arvense	WOA	44.03N	79.29W	C	H	Fallow field	NO	Caryophyllaceae	Insect	0.52	5
892	Cerastium arvense	WOA	44.03N	79.29W	C	H	Fallow field	NO	Caryophyllaceae	Insect	0.15	6
893	Cerastium fontanum	WOA	44.03N	79.29W	C	H	Fallow field	NO	Caryophyllaceae	Insect	1.16	5
894	Cerastium fontanum	WOA	44.03N	79.29W	C	H	Fallow field	NO	Caryophyllaceae	Insect	12.73	6
895	Chenopodium album	WOA	44.03N	79.29W	C	H	Fallow field	NO	Amaranthaceae	Insect	10.47	5
896	Chenopodium simplex	WOA	44.03N	79.29W	C	H	Fallow field	NO	Amaranthaceae	Insect	7.54	5
897	Cynanchum rossicum	WOA	44.03N	79.29W	C	V	Fallow field	NO	Apocynaceae	Insect	0.96	5
898	Cynanchum rossicum	WOA	44.03N	79.29W	C	V	Fallow field	NO	Apocynaceae	Insect	0.65	6
899	Elymus repens	WOA	44.03N	79.29W	C	H	Fallow field	NO	Poaceae	Insect	4.57	5
900	Elymus repens	WOA	44.03N	79.29W	C	H	Fallow field	NO	Poaceae	Insect	0.37	6
901	Elymus trachycaulus	WOA	44.03N	79.29W	C	H	Fallow field	NO	Poaceae	Insect	2.05	5
902	Elymus trachycaulus	WOA	44.03N	79.29W	C	H	Fallow field	NO	Poaceae	Insect	0.84	6
903	Galium boreale	WOA	44.03N	79.29W	C	H	Fallow field	NO	Rubiaceae	Insect	0.76	5
904	Galium boreale	WOA	44.03N	79.29W	C	H	Fallow field	NO	Rubiaceae	Insect	0.25	6
905	Galium verum	WOA	44.03N	79.29W	C	V	Fallow field	NO	Rubiaceae	Insect	0.20	5
906	Galium verum	WOA	44.03N	79.29W	C	V	Fallow field	NO	Rubiaceae	Insect	0.90	6
907	Geum aleppicum	WOA	44.03N	79.29W	C	H	Fallow field	NO	Rosaceae	Insect	3.45	5
908	Geum aleppicum	WOA	44.03N	79.29W	C	H	Fallow field	NO	Rosaceae	Insect	20.64	6
909	Geum urbanum	WOA	44.03N	79.29W	C	H	Fallow field	NO	Rosaceae	Insect	6.74	5
910	Geum urbanum	WOA	44.03N	79.29W	C	H	Fallow field	NO	Rosaceae	Insect	11.01	6
911	Lactuca canadensis	WOA	44.03N	79.29W	C	H	Fallow field	NO	Asteraceae	Insect	13.40	5
912	Lactuca canadensis	WOA	44.03N	79.29W	C	H	Fallow field	NO	Asteraceae	Insect	5.59	6
913	Lactuca serriola	WOA	44.03N	79.29W	C	H	Fallow field	NO	Asteraceae	Insect	3.81	5
914	Lactuca serriola	WOA	44.03N	79.29W	C	H	Fallow field	NO	Asteraceae	Insect	3.82	6
915	Lepidium campestre	WOA	44.03N	79.29W	C	H	Fallow field	NO	Brassicaceae	Insect	39.57	5
916	Lepidium campestre	WOA	44.03N	79.29W	C	H	Fallow field	NO	Brassicaceae	Insect	16.93	6
917	Lepidium densiflorum	WOA	44.03N	79.29W	C	H	Fallow field	NO	Brassicaceae	Insect	32.27	5
918	Lepidium densiflorum	WOA	44.03N	79.29W	C	H	Fallow field	NO	Brassicaceae	Insect	33.57	6
919	Plantago major	WOA	44.03N	79.29W	C	H	Fallow field	NO	Plantaginaceae	Insect	15.00	5
920	Plantago major	WOA	44.03N	79.29W	C	H	Fallow field	NO	Plantaginaceae	Insect	35.66	6
921	Plantago rugellii	WOA	44.03N	79.29W	C	H	Fallow field	NO	Plantaginaceae	Insect	6.02	5
922	Plantago rugellii	WOA	44.03N	79.29W	C	H	Fallow field	NO	Plantaginaceae	Insect	34.93	6

923	Potentilla arguta	WOA	44.03N	79.29W	C	S	Fallow field	NO	Rosaceae	Insect	1.80	5
924	Potentilla arguta	WOA	44.03N	79.29W	C	S	Fallow field	NO	Rosaceae	Insect	5.06	6
925	Potentilla recta	WOA	44.03N	79.29W	C	H	Fallow field	NO	Rosaceae	Insect	3.01	5
926	Potentilla recta	WOA	44.03N	79.29W	C	H	Fallow field	NO	Rosaceae	Insect	7.27	6
927	Senecio pauperculus	WOA	44.03N	79.29W	C	H	Fallow field	NO	Asteraceae	Insect	4.49	5
928	Senecio vulgaris	WOA	44.03N	79.29W	C	H	Fallow field	NO	Asteraceae	Insect	7.58	5
929	Silene antirrhina	WOA	44.03N	79.29W	C	H	Fallow field	NO	Caryophyllaceae	Insect	0.32	5
930	Silene antirrhina	WOA	44.03N	79.29W	C	H	Fallow field	NO	Caryophyllaceae	Insect	0.51	6
931	Silene vulgaris	WOA	44.03N	79.29W	C	H	Fallow field	NO	Caryophyllaceae	Insect	1.04	5
932	Silene vulgaris	WOA	44.03N	79.29W	C	H	Fallow field	NO	Caryophyllaceae	Insect	0.67	6
933	Quercus ilex	WOA	44.44N	0.46W	T	T	Forest	NO	Fagaceae	Insect	10.11	73
934	Quercus robur	WOA	44.44N	0.46W	T	T	Forest	NO	Fagaceae	Insect	24.38	73
935	Pontederia cordata	WOA	45.22N	75.41W	C	H	Wetland	NO	Pontederiaceae	NA	1.88	209
936	Tsuga canadensis	WOA	45.22N	75.41W	C	T	Forest	NO	Pinaceae	NA	0.88	209
937	Larix laricina	WOA	45.23N	75.42W	C	T	Forest	NO	Pinaceae	NA	0.60	209
938	Pinus strobus	WOA	45.23N	75.42W	C	T	Forest	NO	Pinaceae	NA	0.28	209
939	Sagittaria latifolia	WOA	45.23N	75.42W	C	H	Wetland	NO	Alismataceae	NA	1.01	209
940	Acer rubrum	WOA	45.25N	93.1W	C	T	Field	NO	Sapindaceae	Insect	3.38	173
941	Anemone cylindrica	WOA	45.25N	93.12W	C	H	Fallow field	NO	Ranunculaceae	Insect	3.08	173
942	Corylus americana	WOA	45.25N	93.12W	C	S	Fallow field	NO	Betulaceae	Insect	5.99	173
943	Fragaria virginiana	WOA	45.25N	93.12W	C	H	Fallow field	NO	Rosaceae	Insect	3.42	173
944	Lathyrus venosus	WOA	45.25N	93.12W	C	V	Fallow field	NO	Fabaceae	Insect	1.60	173
945	Lespedeza capitata	WOA	45.25N	93.12W	C	H	Grassland	NO	Fabaceae	Insect	8.91	107
946	Monarda fistulosa	WOA	45.25N	93.12W	C	H	Fallow field	NO	Lamiaceae	Insect	2.88	173
947	Potentilla recta	WOA	45.25N	93.12W	C	H	Fallow field	NO	Rosaceae	Insect	2.27	173
948	Quercus ellipsoidalis	WOA	45.25N	93.12W	C	T	Fallow field	NO	Fagaceae	Insect	8.04	173
949	Rubus allegheniensis	WOA	45.25N	93.12W	C	H	Fallow field	NO	Rosaceae	Insect	2.73	173
950	Solidago gigantea	WOA	45.25N	93.12W	C	H	Fallow field	NO	Asteraceae	Insect	2.04	173
951	Solidago missouriensis	WOA	45.25N	93.12W	C	H	Fallow field	NO	Asteraceae	Insect	3.03	173
952	Solidago rigida	WOA	45.25N	93.12W	C	H	Fallow field	NO	Asteraceae	Insect	4.69	173
953	Abies balsamea	WOA	45.26N	81.36W	C	T	Unclear	NO	Pinaceae	NA	3.40	207
954	Thuja occidentalis	WOA	45.26N	81.36W	C	T	Unclear	NO	Cupressaceae	NA	1.91	207
955	Brasenia schreberi	WOA	46.22N	82.39W	C	H	Unclear	NO	Nymphaeaceae	NA	2.30	207

956	Nuphar variegatum	WOA	46.22N	82.39W	C	H	Unclear	NO	Nymphaeaceae	NA	6.03	207
957	Nymphaea odorata	WOA	46.22N	82.39W	C	H	Unclear	NO	Nymphaeaceae	NA	3.60	207
958	Juniperus communis	WOA	46.56N	84.4W	C	T	Unclear	NO	Cupressaceae	NA	0.30	207
959	Picea glauca	WOA	46.56N	84.4W	C	T	Unclear	NO	Pinaceae	NA	2.03	207
960	Pinus banksiana	WOA	46.56N	84.4W	C	T	Unclear	NO	Pinaceae	NA	0.60	207
961	Taxus canadensis	WOA	46.56N	84.4W	C	S	Unclear	NO	Taxaceae	NA	0.04	207
962	Abies grandis	WOA	46.94N	121.33W	C	T	Forest	NO	Pinaceae	Insect	0.33	175
963	Pseudotsuga menziesii	WOA	46.94N	121.33W	C	T	Forest	NO	Pinaceae	Insect	2.00	175
964	Thuja plicata	WOA	46.94N	121.33W	C	T	Forest	NO	Cupressaceae	Insect	0.33	175
965	Tsuga heterophylla	WOA	46.94N	121.33W	C	T	Forest	NO	Pinaceae	Insect	0.00	175
966	Pinus resinosa	WOA	47.19N	84.36W	C	T	Unclear	NO	Pinaceae	NA	4.27	207
967	Cystopteris fragilis	WOA	47.21N	84.41W	C	H	Unclear	NO	Woodsiaceae	NA	6.90	207
968	Gymnocarpium dryopteris	WOA	47.21N	84.41W	C	H	Unclear	NO	Woodsiaceae	NA	4.85	207
969	Phegopteris connectilis	WOA	47.21N	84.41W	C	H	Unclear	NO	Thelypteridaceae	NA	11.55	207
970	Polypodium virginianum	WOA	47.21N	84.41W	C	H	Unclear	NO	Polypodiaceae	NA	0.74	207
971	Betonica officinalis	WOA	47.22N	7.25E	C	H	Grassland	NO	Lamiaceae	Others	3.50	188
972	Betonica officinalis	WOA	47.25N	7.2E	C	H	Grassland	NO	Lamiaceae	Others	2.29	188
973	Betonica officinalis	WOA	47.27N	7.34E	C	H	Grassland	NO	Lamiaceae	Others	1.36	188
974	Larix americana	WOA	47.33N	84.31W	C	T	Unclear	NO	Pinaceae	NA	0.80	207
975	Nymphaea odorata	WOA	47.33N	84.31W	C	H	Unclear	NO	Nymphaeaceae	NA	24.22	207
976	Osmunda claytoniana	WOA	47.33N	84.31W	C	H	Unclear	NO	Osmundaceae	NA	1.30	207
977	Phegopteris dryopteris	WOA	47.33N	84.31W	C	H	Unclear	NO	Thelypteridaceae	NA	1.97	207
978	Picea mariana	WOA	47.33N	84.31W	C	T	Unclear	NO	Pinaceae	NA	0.00	207
979	Abies grandis	WOA	47.45N	120.44W	C	T	Forest	NO	Pinaceae	Insect	0.10	178
980	Pseudotsuga menziesii	WOA	47.45N	120.44W	C	T	Forest	NO	Pinaceae	Insect	0.40	178
981	Taxus brevifolia	WOA	47.45N	120.44W	C	T	Forest	NO	Taxaceae	Insect	0.80	178
982	Thuja plicata	WOA	47.45N	120.44W	C	T	Forest	NO	Cupressaceae	Insect	0.10	178
983	Tsuga heterophylla	WOA	47.45N	120.44W	C	T	Forest	NO	Pinaceae	Insect	0.30	178
984	Cornus sanguinea	WOA	48.31N	9.03E	T	S	Forest	NO	Cornaceae	Insect	3.32	94
985	Crataegus monogyna	WOA	48.31N	9.03E	T	S	Forest	NO	Rosaceae	Insect	5.68	94
986	Euonymus europaeus	WOA	48.31N	9.03E	T	S	Forest	NO	Celastraceae	Insect	16.67	94
987	Ligustrum vulgare	WOA	48.31N	9.03E	T	S	Forest	NO	Oleaceae	Insect	1.40	94
988	Lonicera xylosteum	WOA	48.31N	9.03E	T	S	Forest	NO	Caprifoliaceae	Insect	1.62	94

989	Rosa canina	WOA	48.31N	9.03E	T	V	Forest	NO	Rosaceae	Insect	17.49	94
990	Viburnum lantana	WOA	48.31N	9.03E	T	S	Forest	NO	Adoxaceae	Insect	12.55	94
991	Senecio ovatus	WOA	50.36N	13.17E	C	H	Field	NO	Asteraceae	Insect	20.60	158
992	Rumex acetosa	WOA	50.55N	11.35E	C	H	Grassland	NO	Polygonaceae	Insect	5.10	171
993	Achillea millefolium	WOA	50.57N	11.37E	C	H	Grassland	NO	Asteraceae	Insect	0.51	114
994	Alopecurus pratensis	WOA	50.57N	11.37E	C	H	Grassland	NO	Poaceae	Insect	1.38	114
995	Anthoxanthum odoratum	WOA	50.57N	11.37E	C	H	Grassland	NO	Poaceae	Insect	1.14	114
996	Anthriscus sylvestris	WOA	50.57N	11.37E	C	H	Grassland	NO	Apiaceae	Insect	0.51	114
997	Arrhenatherum elatius	WOA	50.57N	11.37E	C	H	Grassland	NO	Poaceae	Insect	0.76	114
998	Avenula pubescens	WOA	50.57N	11.37E	C	H	Grassland	NO	Poaceae	Insect	2.46	114
999	Bellis perennis	WOA	50.57N	11.37E	C	H	Grassland	NO	Asteraceae	Insect	2.30	114
1000	Bromus erectus	WOA	50.57N	11.37E	C	H	Grassland	NO	Poaceae	Insect	0.92	114
1001	Bromus hordeaceus	WOA	50.57N	11.37E	C	H	Grassland	NO	Poaceae	Insect	2.23	114
1002	Carum carvi	WOA	50.57N	11.37E	C	H	Grassland	NO	Apiaceae	Insect	0.67	114
1003	Cirsium oleraceum	WOA	50.57N	11.37E	C	H	Grassland	NO	Asteraceae	Insect	1.32	114
1004	Crepis biennis	WOA	50.57N	11.37E	C	H	Grassland	NO	Asteraceae	Insect	4.33	114
1005	Dactylis glomerata	WOA	50.57N	11.37E	C	H	Grassland	NO	Poaceae	Insect	0.65	114
1006	Daucus carota	WOA	50.57N	11.37E	C	H	Grassland	NO	Apiaceae	Insect	0.66	114
1007	Festuca pratensis	WOA	50.57N	11.37E	C	H	Grassland	NO	Poaceae	Insect	1.31	114
1008	Festuca rubra	WOA	50.57N	11.37E	C	H	Grassland	NO	Poaceae	Insect	1.84	114
1009	Galium mollugo agg.	WOA	50.57N	11.37E	C	H	Grassland	NO	Rubiaceae	Insect	0.88	114
1010	Geranium pratense	WOA	50.57N	11.37E	C	H	Grassland	NO	Geraniaceae	Insect	1.11	114
1011	Glechoma hederacea	WOA	50.57N	11.37E	C	H	Grassland	NO	Lamiaceae	Insect	2.96	114
1012	Heracleum sphondylium	WOA	50.57N	11.37E	C	H	Grassland	NO	Apiaceae	Insect	1.28	114
1013	Holcus lanatus	WOA	50.57N	11.37E	C	H	Grassland	NO	Poaceae	Insect	0.43	114
1014	Knautia arvensis	WOA	50.57N	11.37E	C	H	Grassland	NO	Caprifoliaceae	Insect	0.37	114
1015	Lathyrus pratensis	WOA	50.57N	11.37E	C	V	Grassland	NO	Fabaceae	Insect	2.69	114
1016	Leontodon autumnalis	WOA	50.57N	11.37E	C	H	Grassland	NO	Asteraceae	Insect	1.10	114
1017	Leontodon hispidus	WOA	50.57N	11.37E	C	H	Grassland	NO	Asteraceae	Insect	0.10	114
1018	Leucanthemum vulgare	WOA	50.57N	11.37E	C	H	Grassland	NO	Asteraceae	Insect	1.30	114
1019	Lotus corniculatus	WOA	50.57N	11.37E	C	H	Grassland	NO	Fabaceae	Insect	1.38	114
1020	Luzula campestris	WOA	50.57N	11.37E	C	H	Grassland	NO	Juncaceae	Insect	1.21	114
1021	Medicago lupulina	WOA	50.57N	11.37E	C	H	Grassland	NO	Fabaceae	Insect	3.98	114

1022	Medicago varia	WOA	50.57N	11.37E	C	H	Grassland	NO	Fabaceae	Insect	3.71	114
1023	Onobrychis viciifolia	WOA	50.57N	11.37E	C	H	Grassland	NO	Fabaceae	Insect	0.78	114
1024	Pastinaca sativa	WOA	50.57N	11.37E	C	H	Grassland	NO	Apiaceae	Insect	0.45	114
1025	Phleum pratense	WOA	50.57N	11.37E	C	H	Grassland	NO	Poaceae	Insect	0.55	114
1026	Pimpinella major	WOA	50.57N	11.37E	C	H	Grassland	NO	Apiaceae	Insect	0.19	114
1027	Plantago lanceolata	WOA	50.57N	11.37E	C	H	Grassland	NO	Plantaginaceae	Insect	1.82	114
1028	Plantago media	WOA	50.57N	11.37E	C	H	Grassland	NO	Plantaginaceae	Insect	0.52	114
1029	Poa pratensis	WOA	50.57N	11.37E	C	H	Grassland	NO	Poaceae	Insect	1.67	114
1030	Poa trivialis	WOA	50.57N	11.37E	C	H	Grassland	NO	Poaceae	Insect	1.97	114
1031	Primula veris	WOA	50.57N	11.37E	C	H	Grassland	NO	Primulaceae	Insect	0.37	114
1032	Prunella vulgaris	WOA	50.57N	11.37E	C	H	Grassland	NO	Lamiaceae	Insect	2.05	114
1033	Ranunculus acris	WOA	50.57N	11.37E	C	H	Grassland	NO	Ranunculaceae	Insect	0.80	114
1034	Ranunculus repens	WOA	50.57N	11.37E	C	H	Grassland	NO	Ranunculaceae	Insect	2.16	114
1035	Rumex acetosa	WOA	50.57N	11.37E	C	H	Grassland	NO	Polygonaceae	Insect	2.25	114
1036	Sanguisorba officinalis	WOA	50.57N	11.37E	C	H	Grassland	NO	Rosaceae	Insect	0.65	114
1037	Taraxacum officinale	WOA	50.57N	11.37E	C	H	Grassland	NO	Asteraceae	Insect	0.32	114
1038	Tragopogon pratensis	WOA	50.57N	11.37E	C	H	Grassland	NO	Asteraceae	Insect	0.48	114
1039	Trifolium campestre	WOA	50.57N	11.37E	C	H	Grassland	NO	Fabaceae	Insect	6.49	114
1040	Trifolium dubium	WOA	50.57N	11.37E	C	H	Grassland	NO	Fabaceae	Insect	2.71	114
1041	Trifolium fragiferum	WOA	50.57N	11.37E	C	H	Grassland	NO	Fabaceae	Insect	10.21	114
1042	Trifolium hybridum	WOA	50.57N	11.37E	C	H	Grassland	NO	Fabaceae	Insect	10.61	114
1043	Trifolium pratense	WOA	50.57N	11.37E	C	H	Grassland	NO	Fabaceae	Insect	5.26	114
1044	Trifolium repens	WOA	50.57N	11.37E	C	H	Grassland	NO	Fabaceae	Insect	6.67	114
1045	Trisetum flavescens	WOA	50.57N	11.37E	C	H	Grassland	NO	Poaceae	Insect	1.83	114
1046	Veronica chamaedrys	WOA	50.57N	11.37E	C	H	Grassland	NO	Plantaginaceae	Insect	0.39	114
1047	Vicia cracca	WOA	50.57N	11.37E	C	H	Grassland	NO	Fabaceae	Insect	3.68	114
1048	Fagus sylvatica	WOA	51.1N	10.5E	C	T	Forest	NO	Fagaceae	Insect	1.50	184
1049	Arnica montana	WOA	51.44N	10.29E	C	H	Grassland	NO	Asteraceae	Others	2.75	26
1050	Fagus sylvatica	WOA	53.33N	2.483W	T	T	Forest	NO	Fagaceae	Insect	3.14	150
1051	Fagus sylvatica	WOA	56.17N	10.27E	C	T	Forest	NO	Fagaceae	Insect	5.68	141
1052	Tilia cordata	WOA	56.51N	53.12E	C	T	Urban field	NO	Malvaceae	Insect	35.20	51
1053	Arabidopsis lyrata	WOA	59.28N	6.49E	C	H	Disturbed soil	NO	Brassicaceae	Insect	0.10	113

1054	Arabidopsis lyrata	WOA	59.39N	6.58E	C	H	Disturbed soil	NO	Brassicaceae	Insect	1.59	113
1055	Arabidopsis lyrata	WOA	60.24N	7.1E	C	H	Disturbed soil	NO	Brassicaceae	Insect	2.48	113
1056	Nuphar lutea	WOA	60.4N	25E	C	H	Grassland	NO	Nymphaeaceae	Insect	17.40	104
1057	Achillea millefolium	WOA	61N	138W	C	H	Forest	NO	Asteraceae	Insect	0.78	88
1058	Achillea millefolium	WOA	61N	138W	C	H	Grassland	NO	Asteraceae	Insect	0.91	88
1059	Arctostaphylos uvaursi	WOA	61N	138W	C	S	Forest	NO	Ericaceae	Insect	1.21	88
1060	Artemisia norvegica	WOA	61N	138W	C	H	Forest	NO	Asteraceae	Insect	0.38	88
1061	Carex consimilis	WOA	61N	138W	C	H	Forest	NO	Cyperaceae	Insect	0.24	88
1062	Dryas octopetala	WOA	61N	138W	C	S	Forest	NO	Rosaceae	Insect	0.00	88
1063	Festuca altaica	WOA	61N	138W	C	H	Forest	NO	Poaceae	Insect	0.00	88
1064	Festuca campestris	WOA	61N	138W	C	H	Grassland	NO	Poaceae	Insect	0.54	88
1065	Galium boreale	WOA	61N	138W	C	H	Grassland	NO	Rubiaceae	Insect	11.83	88
1066	Mertensia paniculata	WOA	61N	138W	C	H	Forest	NO	Boraginaceae	Insect	5.97	88
1067	Polygonum viviparum	WOA	61N	138W	C	H	Forest	NO	Polygonaceae	Insect	0.97	88
1068	Rosa arkansana	WOA	61N	138W	C	S	Grassland	NO	Rosaceae	Insect	2.68	88
1069	Salix reticulata	WOA	61N	138W	C	S	Forest	NO	Salicaceae	Insect	2.01	88
1070	Thermopsis rhombifolia	WOA	61N	138W	C	H	Grassland	NO	Fabaceae	Insect	70.18	88
1071	Arabidopsis lyrata	WOA	61.06N	7.3E	p	H	Disturbed soil	NO	Brassicaceae	Insect	2.00	113
1072	Arabidopsis lyrata	WOA	61.18N	7.51E	p	H	Disturbed soil	NO	Brassicaceae	Insect	6.81	113
1073	Salix phylicifolia	WOA	61.33N	29.33E	C	T	Forest	NO	Salicaceae	Insect	5.76	182
1074	Salix phylicifolia	WOA	61.33N	29.33E	C	T	Forest	NO	Salicaceae	Insect	6.94	183
1075	Arabidopsis lyrata	WOA	61.41N	8.35E	p	H	Disturbed soil	NO	Brassicaceae	Insect	2.68	113
1076	Arabidopsis lyrata	WOA	61.41N	8.25E	p	H	Disturbed soil	NO	Brassicaceae	Insect	0.61	113
1077	Arabidopsis lyrata	WOA	61.42N	8.23E	p	H	Disturbed soil	NO	Brassicaceae	Insect	0.88	113
1078	Arabidopsis lyrata	WOA	61.42N	6.51E	p	H	Disturbed soil	NO	Brassicaceae	Insect	4.33	113

1079	Arabidopsis lyrata	WOA	62.03N	5.34E	p	H	Disturbed soil	NO	Brassicaceae	Insect	2.12	113
1080	Arabidopsis lyrata	WOA	62.3N	9.05E	C	H	Disturbed soil	NO	Brassicaceae	Insect	1.27	113
1081	Arabidopsis lyrata	WOA	62.33N	17.56E	C	H	Disturbed soil	NO	Brassicaceae	Insect	2.16	113
1082	Arabidopsis lyrata	WOA	62.35N	18.02E	C	H	Disturbed soil	NO	Brassicaceae	Insect	3.19	113
1083	Arabidopsis lyrata	WOA	62.35N	18.01E	C	H	Disturbed soil	NO	Brassicaceae	Insect	4.60	113
1084	Arabidopsis lyrata	WOA	62.36N	18.03E	C	H	Disturbed soil	NO	Brassicaceae	Insect	5.66	113
1085	Salix phylicifolia	WOA	62.37N	29.4E	C	T	Forest	NO	Salicaceae	Insect	1.37	183
1086	Arabidopsis lyrata	WOA	62.39N	9.39E	C	H	Disturbed soil	NO	Brassicaceae	Insect	0.82	113
1087	Arabidopsis lyrata	WOA	62.42N	18.08E	C	H	Disturbed soil	NO	Brassicaceae	Insect	3.20	113
1088	Arabidopsis lyrata	WOA	62.44N	18.09E	C	H	Disturbed soil	NO	Brassicaceae	Insect	5.98	113
1089	Arabidopsis thaliana	WOA	62.48N	18.12E	C	H	Grassland	NO	Brassicaceae	Others	0.88	8
1090	Arabidopsis lyrata	WOA	62.49N	18.18E	C	H	Disturbed soil	NO	Brassicaceae	Insect	5.49	113
1091	Arabidopsis lyrata	WOA	62.5N	18.22E	C	H	Disturbed soil	NO	Brassicaceae	Insect	4.04	113
1092	Arabidopsis lyrata	WOA	62.52N	18.25E	C	H	Disturbed soil	NO	Brassicaceae	Insect	6.21	113
1093	Arabidopsis lyrata	WOA	62.54N	18.23E	C	H	Disturbed soil	NO	Brassicaceae	Insect	3.82	113
1094	Arabidopsis lyrata	WOA	62.58N	18.32E	C	H	Disturbed soil	NO	Brassicaceae	Insect	3.23	113
1095	Arabidopsis lyrata	WOA	62.59N	18.31E	C	H	Disturbed soil	NO	Brassicaceae	Insect	2.68	113

1096	Arabidopsis lyrata	WOA	63.11N	19E	C	H	Disturbed soil	NO	Brassicaceae	Insect	3.65	113
1097	Arabidopsis lyrata	WOA	63.11N	18.48E	C	H	Disturbed soil	NO	Brassicaceae	Insect	5.96	113
1098	Arabidopsis lyrata	WOA	63.11N	18.46E	C	H	Disturbed soil	NO	Brassicaceae	Insect	3.84	113
1099	Arabidopsis lyrata	WOA	63.12N	18.57E	C	H	Disturbed soil	NO	Brassicaceae	Insect	4.41	113
1100	Arabidopsis lyrata	WOA	63.12N	18.53E	C	H	Disturbed soil	NO	Brassicaceae	Insect	5.73	113
1101	Arabidopsis lyrata	WOA	63.13N	18.58E	C	H	Disturbed soil	NO	Brassicaceae	Insect	3.18	113
1102	Salix triandra	WOA	63.43N	19.31E	C	T	River valley	NO	Salicaceae	Insect	9.16	89
1103	Vaccinium vitisidaea	WOA	68N	19E	C	S	Forest	NO	Ericaceae	Insect	0.05	163
1104	Chamaecrista debilis	WA	21.36S	47.48W	T	S	Savanna	F	Fabaceae	Insect	77.64	44
1105	Rhizophora stylosa	WA	19.17S	147.03E	T	T	Savanna	F	Rhizophoraceae	Insect	6.20	95
1106	Lafoensia pacari	WA	18.57S	48.12W	Tro	T	Savanna	F	Lythraceae	NA	9.08	103
1107	Qualea parviflora	WA	18.21S	45.19W	Tro	T	Savanna	F	Vochysiaceae	Insect	8.12	74
1108	Lumnitzera littorea	WA	18.15S	146.12E	T	T	Forest	F	Combretaceae	Insect	4.30	165
1109	Rhizophora stylosa	WA	18.15S	146.12E	T	T	Forest	F	Rhizophoraceae	Insect	7.60	165
1110	Rhizophora mucronata	WA	18.01S	146.02E	T	T	Forest	N & F	Rhizophoraceae	Insect	3.50	95
1111	Banisteriopsis campestris	WA	15.56S	44.55W	Tro	H	Savanna	F	Malpighiaceae	Insect	14.55	122
1112	Qualea grandiflora	WA	15.56S	44.55W	Tro	T	Savanna	F	Vochysiaceae	Insect	7.15	122
1113	Qualea parviflora	WA	15.56S	44.55W	Tro	T	Savanna	F	Vochysiaceae	Insect	12.60	122
1114	Rhizophora mucronata	WA	9.285S	147.11E	Tro	T	Mangrove	N & F	Rhizophoraceae	Insect	2.60	165
1115	Rhizophora stylosa	WA	9.285S	147.11E	Tro	T	Mangrove	F	Rhizophoraceae	Insect	5.10	165
1116	Hevea brasiliensis	WA	5.053S	38.38E	Tro	T	Forest	F	Euphorbiaceae	NA	1.23	39
1117	Hura crepitans	WA	5.053S	38.38E	Tro	T	Forest	F	Euphorbiaceae	NA	4.33	39
1118	Cedrela odorata	WA	5.05S	38.3E	Tro	T	Forest	F	Meliaceae	NA	5.00	39
1119	Cordia alliodora	WA	5.05S	38.3E	Tro	T	Forest	N & F	Boraginaceae	NA	6.17	39
1120	Maieta guianensis	WA	1.47S	59.48W	Tro	T	Forest	N & F	Melastomataceae	NA	5.05	196
1121	Tococa bullifera	WA	1.47S	59.48W	Tro	T	Forest	N & F	Melastomataceae	NA	11.05	196
1122	Diospyros abyssinica	WA	0.27N	30.25E	Tro	T	Forest	F	Ebenaceae	Insect	3.00	28

1123	Parinari excelsa	WA	0.27N	30.25E	Tro	T	Forest	F	Chrysobalanaceae	Insect	3.00	28
1124	Spathodea campanulata	WA	0.27N	30.25E	Tro	T	Forest	F	Bignoniaceae	Insect	3.00	28
1125	Shorea spp.	WA	1.43N	110.27E	Tro	T	Forest	F	Dipterocarpaceae	Insect	12.75	36
1126	Leonardoxa africana	WA	2.34N	9.5E	Tro	T	Forest	N & F	Fabaceae	Insect	1.89	72
1127	Shorea leprosula	WA	4.54N	117.48E	Tro	T	Forest	F	Dipterocarpaceae	Insect	9.67	83
1128	Shorea leprosula	WA	4.571N	117.4E	Tro	T	Forest	F	Dipterocarpaceae	NA	17.05	83
1129	Shorea leprosula	WA	4.571N	117.4E	Tro	T	Forest	F	Dipterocarpaceae	NA	24.19	124
1130	Cecropia peltata	WA	8.59N	79.33W	Tro	T	Forest	N & F	Moraceae	Insect	2.38	195
1131	Gouania lupuloides	WA	8.59N	79.33W	A	T	Savanna	F	Rhamnaceae	Insect	8.71	195
1132	Alchornea costaricensis	WA	9.08N	79.5W	A	T	Forest	F	Euphorbiaceae	Insect	68.67	162
1133	Inga goldmanii	WA	9.09N	79.51W	Tro	T	Forest	F	Fabaceae	Insect	22.80	35
1134	Inga goldmanii	WA	9.09N	79.51W	Tro	T	Forest	F	Fabaceae	Insect	22.80	106
1135	Tabebuia rosea	WA	9.193N	79.38W	A	T	Field	F	Bignoniaceae	Insect	8.47	156
1136	Tabebuia rosea	WA	9.213N	79.54W	A	T	Plantation	F	Bignoniaceae	Insect	4.27	129
1137	Rhizophora mucronata	WA	9.5N	98.35E	Tro	T	Mangrove	N & F	Rhizophoraceae	Others	0.35	147
1138	Rhizophora mucronata	WA	9.5N	98.35E	Tro	T	Mangrove	N & F	Rhizophoraceae	Others	1.55	145
1139	Theobroma cacao	WA	9.51N	83.39W	Tro	T	Agricultural	F	Malvaceae	Insect	9.70	52
1140	Piper arieianum	WA	10.25N	84.01W	Tro	S	Forest	F	Piperaceae	Insect	13.55	119
1141	Bidens pilosa	WA	10.42N	61.17W	Tro	H	Forest	F	Asteraceae	NA	0.40	206
1142	Cecropia peltata	WA	18.1N	65.3W	Tro	T	Forest	N & F	Moraceae	Insect	1.52	174
1143	Calathea ovandensis	WA	18.26N	95.1W	Tro	H	Forest	F	Marantaceae	Insect	3.65	90
1144	Cordia alliodora	WA	18.36N	95W	Tro	T	Forest	N & F	Boraginaceae	Insect	24.16	40
1145	Costus spicatus	WA	18.36N	95W	Tro	H	Forest	F	Costaceae	Insect	8.20	40
1146	Guarea glabra	WA	18.36N	95W	Tro	T	Forest	F	Meliaceae	Insect	18.30	40
1147	Bunchosia lindeniana	WA	18.36N	95.07W	Tro	T	Forest	F	Malpighiaceae	Insect	18.80	40
1148	Ceiba pentandra	WA	18.36N	95.07W	Tro	T	Forest	F	Malvaceae	Insect	9.15	40
1149	Coffea arabica	WA	18.6N	93W	Tro	T	Agricultural	N & F	Rubiaceae	Insect	2.50	52
1150	Theobroma cacao	WA	18.6N	93W	Tro	T	Agricultural	F	Malvaceae	Insect	4.90	52
1151	Polypodium plebeium	WA	19.3N	96.57W	Tro	H	Forest	F	Polypodiaceae	Insect	21.60	127
1152	Cordia alliodora	WA	19.3N	105W	Tro	T	Forest	N & F	Boraginaceae	Insect	9.27	38
1153	Cordia alliodora	WA	19.3N	105W	Tro	T	Forest	N & F	Boraginaceae	Insect	4.78	56
1154	Coccoloba barbadensis	WA	19.3N	105.03W	Tro	T	Forest	F	Polygonaceae	Insect	17.65	38
1155	Cochlospermum vitifolium	WA	19.3N	105.03W	Tro	T	Forest	F	Bixaceae	Insect	4.18	56

1156	Plumeria rubra	WA	19.3N	105.03W	Tro	T	Forest	F	Apocynaceae	Insect	6.68	56
1157	Prosopis spp.	WA	19.3N	105.03W	Tro	S	Forest	F	Fabaceae	Insect	13.90	38
1158	Cordia alliodora	WA	20.9N	103W	T	T	Forest	N & F	Boraginaceae	Insect	3.70	149
1159	Conocarpus erectus	WA	23.28N	75.49W	Tro	S	Grassland	F	Combretaceae	Insect	8.96	185
1160	Laguncularia racemosa	WA	27.45N	82.34W	Tro	T	Forest	F	Combretaceae	Others	0.39	50
1161	Diospyros virginiana	WA	29.38N	82.2W	Tro	T	Sand dune	F	Ebenaceae	Insect	12.36	99
1162	Gossypium thurberi	WA	31.41N	110.57W	A	S	Desert	F	Malvaceae	Insect	5.16	166
1163	Robinia pseudoacacia	WA	35.12N	83.25W	T	T	Forest	F	Fabaceae	Insect	15.00	81
1164	Robinia pseudoacacia	WA	35.12N	83.25W	T	T	Forest	F	Fabaceae	Insect	27.00	98
1165	Robinia pseudoacacia	WA	35.12N	83.25W	T	T	Forest	F	Fabaceae	Insect	11.40	140
1166	Robinia pseudoacacia	WA	35.12N	83.25W	T	T	Forest	F	Fabaceae	Insect	7.36	208
1167	Robinia pseudoacacia	WA	35.33N	83.25W	T	T	Forest	F	Fabaceae	Insect	4.74	208
1168	Cassia fasciculata	WA	35.46N	78.39W	T	H	Field	F	Fabaceae	NA	9.16	208
1169	Robinia pseudoacacia	WA	35.46N	78.39W	T	T	Forest	F	Fabaceae	NA	3.42	208
1170	Cassia fasciculata	WA	35.46N	78.4W	T	H	Field	F	Fabaceae	NA	2.73	208
1171	Robinia pseudoacacia	WA	35.46N	78.4W	T	T	Forest	F	Fabaceae	NA	13.10	208
1172	Robinia pseudoacacia	WA	35.47N	78.39W	T	T	Forest	F	Fabaceae	NA	11.85	208
1173	Yucca filamentosa	WA	35.47N	78.39W	T	S	Roadside field	F	Asparagaceae	NA	0.10	208
1174	Cassia fasciculata	WA	35.47N	78.41W	T	H	Field	F	Fabaceae	NA	1.70	208
1175	Robinia pseudoacacia	WA	35.47N	78.41W	T	T	Forest	F	Fabaceae	NA	3.50	208
1176	Cassia fasciculata	WA	35.48N	78.41W	T	H	Field	F	Fabaceae	NA	1.19	208
1177	Prunus serotina	WA	35.58N	79.09W	T	T	Forest	F	Rosaceae	Insect	3.10	24
1178	Quercus alba	WA	35.58N	79.09W	T	T	Forest	F	Fagaceae	Insect	8.20	24
1179	Robinia pseudoacacia	WA	35.58N	79.09W	T	T	Forest	F	Fabaceae	Insect	5.76	179
1180	Yucca filamentosa	WA	36.28N	79.23W	T	S	Roadside field	F	Asparagaceae	NA	0.11	208
1181	Robinia pseudoacacia	WA	36.4N	78.54W	T	T	Forest	F	Fabaceae	NA	6.03	179
1182	Quercus alba	WA	36.54N	84.21W	T	T	Forest	F	Fagaceae	Insect	14.30	24
1183	Quercus alba	WA	36.54N	84.21W	T	T	Forest	F	Fagaceae	Insect	11.91	16
1184	Robinia pseudoacacia	WA	37.2N	80.32W	T	T	Forest	F	Fabaceae	NA	6.07	179
1185	Robinia pseudoacacia	WA	37.21N	80.32W	T	T	Forest	F	Fabaceae	NA	7.72	179
1186	Robinia pseudoacacia	WA	37.22N	80.31W	T	T	Forest	F	Fabaceae	NA	8.47	179

1187	Chamaecrista fasciculata	WA	37.57N	91.49W	C	H	Grassland	F	Fabaceae	Insect	11.39	2
1188	Quercus alba	WA	38.31N	90.33W	T	T	Forest	F	Fagaceae	Insect	15.61	16
1189	Quercus alba	WA	38.31N	90.33W	T	T	Forest	F	Fagaceae	Insect	2.30	98
1190	Quercus alba	WA	38.31N	90.33W	T	T	Forest	F	Fagaceae	Insect	15.02	3
1191	Chamaecrista fasciculata	WA	38.42N	90.2W	C	H	Grassland	F	Fabaceae	Insect	2.83	2
1192	Chamaecrista fasciculata	WA	38.46N	90.35W	C	H	Forest	F	Fabaceae	Insect	7.90	2
1193	Epilobium angustifolium	WA	38.57N	106.59W	C	H	Shrubland	F	Onagraceae	Insect	12.70	115
1194	Helianthella quinquenervis	WA	38.57N	106.59W	C	H	Shrubland	F	Asteraceae	Insect	7.80	115
1195	Helianthella quinquenervis	WA	38.57N	106.59W	C	H	Shrubland	F	Asteraceae	NA	21.80	140
1196	Populus tremuloides	WA	38.57N	106.59W	T	T	Shrubland	F	Salicaceae	NA	8.00	198
1197	Citrus reticulata	WA	39.29N	0.45W	A	T	Orchard	F	Rutaceae	Insect	38.80	68
1198	Cirsium arvense	WA	39.54N	75.47W	C	H	Grassland	F	Asteraceae	NA	13.46	30
1199	Solidago altissima	WA	42.25N	76.31W	C	H	Fallow field	F	Asteraceae	Insect	4.13	32
1200	Catalpa speciosa	WA	43.1N	73.5W	C	T	Forest	F	Bignoniaceae	NA	10.30	140
1201	Robinia pseudoacacia	WA	43.1N	73.5W	C	T	Forest	F	Fabaceae	NA	5.13	179
1202	Prunus serotina	WA	43.39N	80.26W	C	T	Forest	F	Rosaceae	Insect	4.33	98
1203	Prunus virginiana	WA	43.39N	80.26W	C	T	Forest	F	Rosaceae	Insect	8.80	24
1204	Quercus alba	WA	43.39N	80.26W	C	T	Forest	F	Fagaceae	Insect	17.74	123
1205	Betula pendula	WA	44.44N	0.46W	T	T	Forest	F	Betulaceae	Insect	33.34	73
1206	Picea abies	WA	45.23N	75.42W	C	T	Forest	F	Pinaceae	NA	0.06	209
1207	Populus tremuloides	WA	45.25N	93.12W	T	T	Fallow field	F	Salicaceae	Insect	8.75	198
1208	Acer pseudoplatanus	WA	47.33N	52.4E	A	T	Forest	F	Sapindaceae	Insect	44.30	66
1209	Prunus spinosa	WA	48.31N	9.03E	T	S	Forest	F	Rosaceae	Insect	18.46	94
1210	Centaurea jacea	WA	50.57N	11.37E	T	H	Grassland	F	Asteraceae	Others	1.54	114
1211	Artemisia ludoviciana	WA	61N	138W	C	H	Grassland	F	Asteraceae	Insect	0.93	88
1212	Epilobium angustifolium	WA	61N	138W	C	H	Forest	F	Onagraceae	Insect	1.38	88
1213	Salix myrsinifolia	WA	61.33N	29.33E	C	T	Forest	F	Salicaceae	Insect	1.44	182
1214	Salix myrsinifolia	WA	61.33N	29.33E	C	T	Forest	F	Salicaceae	Insect	1.06	183
1215	Salix myrsinifolia	WA	62.37N	29.4E	C	T	Forest	F	Salicaceae	Insect	3.05	183
1216	Populus tremuloides	WA	64.42N	148.19W	C	T	Forest	F	Salicaceae	Insect	13.00	198
1217	Populus tremuloides	WA	64.42N	148.19W	C	T	Forest	F	Salicaceae	Insect	16.70	140
1218	Populus tremuloides	WA	64.42N	148.19W	C	T	Forest	F	Salicaceae	Insect	1.87	173
1219	Populus tremuloides	WA	64.52N	148.04W	C	T	Forest	F	Salicaceae	Insect	2.43	173

1220	Vaccinium myrtillus	WA	68N	19E	C	S	Forest	F	Ericaceae	Insect	0.10	163
1221	Vaccinium uliginosum	WA	68N	19E	C	S	Forest	F	Ericaceae	Insect	0.54	163
1222	Coffea arabica	WA	15.1N	92.19W	Tro	T	Agricultural	N & F	Rubiaceae	Insect	0.42	151
1223	Coffea arabica	WA	15.11N	92.2W	Tro	T	Agricultural	N & F	Rubiaceae	Insect	1.18	151
1224	Cordia alliodora	WA	19.3N	105.02W	Tro	T	Forest	N & F	Boraginaceae	Insect	0.96	157
1225	Macaranga hosei	WA	3.3N	101.31E	Tro	T	Forest	N & F	Euphorbiaceae	Insect	0.20	84
1226	Rhizophora mucronata	WA	9.5N	98.35E	Tro	T	Mangrove	N & F	Rhizophoraceae	Insect	0.29	147
1227	Coffea arabica	WA	15.15N	92.22W	Tro	T	Agricultural	N & F	Rubiaceae	Insect	2.24	151
1228	Macaranga triloba	WA	3.3N	101.31E	Tro	T	Forest	N & F	Euphorbiaceae	Insect	1.20	84
1229	Mallotus japonicus	WA	34.41N	133.55E	T	T	Unclear	F	Euphorbiaceae	Insect	4.41	202
1230	Macaranga winkleri	WA	4.2N	113.5E	Tro	T	Forest	N & F	Euphorbiaceae	Insect	1.93	91
1231	Conocarpus erectus	WA	24.1N	76.26W	T	T	Mangrove	F	Combretaceae	Insect	2.54	153
1232	Fallopia japonica	WA	42.53N	73.35W	C	H	Unclear	F	Polygonaceae	Insect	2.54	139
1233	Polypodium plebeium	WA	19.32N	96.55W	Tro	F	Forest	F	Polypodiaceae	Insect	0.40	102
1234	Ouratea spectabilis	WA	18.59S	48.18W	Tro	T	Shrubland	F	Ochnaceae	Insect	4.13	29
1235	Macaranga tanarius	WA	3.3N	101.31E	Tro	T	Forest	N & F	Euphorbiaceae	Insect	3.20	84
1236	Tococa formicaria	WA	13.53S	45.42W	Tro	T	Savanna	N	Melastomataceae	Insect	3.62	19
1237	Zanthoxylum myriacanthum	WA	3.17N	101.44E	Tro	T	Forest	N & F	Rutaceae	Insect	3.58	132
1238	Pinus ponderosa	WA	39.06N	105.05W	T	T	Forest	F	Pinaceae	Insect	6.80	133
1239	Conocarpus erectus	WA	23.3N	75.47W	T	T	Mangrove	F	Combretaceae	Insect	4.58	154
1240	Macaranga lamellata	WA	4.2N	113.5E	Tro	T	Forest	N & F	Euphorbiaceae	Insect	2.00	91
1241	Macaranga hulletti	WA	3.14N	101.38E	Tro	T	Forest	N & F	Euphorbiaceae	Insect	1.14	55
1242	Cordia alliodora	WA	10.5N	85.36W	Tro	T	Forest	N & F	Boraginaceae	Insect	6.80	157
1243	Conocarpus erectus	WA	23.5N	75.8W	T	T	Mangrove	F	Combretaceae	Insect	2.75	155
1244	Cordia nodosa	WA	3.37N	72.14W	Tro	T	Forest	N & F	Boraginaceae	Insect	4.49	62
1245	Theobroma cacao	WA	1.23S	121.26E	Tro	T	Agricultural	F	Malvaceae	Insect	5.99	199
1246	Quercus dentata	WA	43.12N	141.15E	C	T	Coast	F	Fagaceae	Insect	3.70	92
1247	Gossypium hirsutum	WA	35.35N	85.29W	T	T	Agricultural	F	Malvaceae	Insect	3.20	190
1248	Fallopia japonica	WA	43.02N	73.45W	C	H	Unclear	F	Polygonaceae	Insect	5.75	139
1249	Harungana madagascariensis	WA	1.19S	9.86E	Tro	T	Forest	F	Guttiferae	Insect	1.63	45
1250	Macaranga beccariana	WA	4.2N	113.5E	Tro	T	Forest	N & F	Euphorbiaceae	Insect	2.00	91
1251	Tabebuia rosea	WA	9.19N	79.38W	A	T	Forest	F	Bignoniaceae	Insect	7.40	164
1252	Gossypium thurberi	WA	31.41N	110.57W	A	S	Sand dune	F	Malvaceae	Insect	3.15	166

1253	Cordia nodosa	WA	12.34S	70.05W	Tro	T	Forest	N & F	Boraginaceae	Insect	5.05	64
1254	Macaranga bullettii	WA	4.2N	113.5E	Tro	T	Forest	N & F	Euphorbiaceae	Insect	3.60	91
1255	Tococa macrosperma	WA	6.21N	62.49W	Tro	S	Shrubland	N	Melastomataceae	Insect	4.16	128
1256	Quercus liaotungensis	WA	39.57N	115.25E	T	T	Forest	F	Fagaceae	Insect	8.50	205
1257	Macaranga hosei	WA	3.14N	101.38E	Tro	T	Forest	N & F	Euphorbiaceae	Insect	1.08	55
1258	Macaranga hypoleuca	WA	3.14N	101.38E	Tro	T	Forest	N & F	Euphorbiaceae	Insect	1.09	55
1259	Hirtella physophora	WA	5.03N	53.02W	Tro	S	Forest	N & F	Chrysobalanaceae	Insect	0.76	75
1260	Macaranga hosei	WA	3.14N	101.38E	Tro	T	Forest	N & F	Euphorbiaceae	Insect	1.08	55
1261	Macaranga hosei	WA	4.2N	113.5E	Tro	T	Forest	N & F	Euphorbiaceae	Insect	2.50	91
1262	Citrus sinensis	WA	10.29N	85.21W	Tro	T	Agricultural	N	Rutaceae	Insect	13.04	79
1263	Duroia hirsuta	WA	3.37N	72.14W	Tro	T	Forest	N	Rubiaceae	Insect	4.78	62
1264	Solanum lycocarpum	WA	18.57S	48.12W	Tro	S	Savanna	F	Solanaceae	Insect	8.70	135
1265	Leonardoxa africana	WA	2.34N	9.5E	Tro	T	Forest	N & F	Fabaceae	Insect	1.89	72
1266	Piper cenocladum	WA	10.25N	84.05W	Tro	S	Forest	N & F	Piperaceae	Insect	13.47	47
1267	Populus fremontii	WA	40.45N	112.07W	C	T	Forest	F	Salicaceae	Insect	7.40	59
1268	Macaranga triloba	WA	3.14N	101.38E	Tro	T	Forest	N & F	Euphorbiaceae	Insect	5.36	55
1269	Chrysothamnus viscidiflorus	WA	38.719N	106.816W	T	S	Grassland	F	Asteraceae	Insect	13.39	76
1270	Tococa guianensis	WA	13.8S	51.5W	Tro	T	Savanna	N & F	Melastomataceae	Insect	12.96	134
1271	Lafoensia pacari	WA	18.57S	48.12W	Tro	T	Savanna	F	Lythraceae	Insect	6.94	103
1272	Inga spectabilis	WA	0.4S	77.24W	Tro	T	Forest	F	Fabaceae	Insect	16.13	25
1273	Hirtella myrmecophila	WA	2.25S	59.48W	Tro	T	Forest	N	Chrysobalanaceae	Insect	0.54	93
1274	Turnera ulmifolia	WA	19.36N	96.22W	Tro	H	Sand dune	F	Turneraceae	Insect	11.68	192
1275	Inga punctata	WA	10.01N	83.45W	Tro	T	Forest	F	Fabaceae	Insect	13.00	101
1276	Macaranga tanarius	WA	3.21N	100.48E	Tro	T	Forest	N & F	Euphorbiaceae	Insect	9.05	54
1277	Vaccinium myrtillus	WA	64N	20.2E	C	S	Forest	F	Ericaceae	Insect	17.71	13
1278	Macaranga bancana	WA	4.2N	113.5E	Tro	T	Forest	N & F	Euphorbiaceae	Insect	3.73	91
1279	Macaranga trachyphylla	WA	4.2N	113.5E	Tro	T	Forest	N & F	Euphorbiaceae	Insect	2.63	91
1280	Leonardoxa africana	WA	2.34N	9.5E	Tro	T	Forest	N & F	Fabaceae	Insect	1.89	71
1281	Solanum lycocarpum	WA	18.55S	48.15W	Tro	H	Savanna	F	Solanaceae	Insect	13.31	42
1282	Anemopaegma scabriusculum	WA	15.27S	43.56W	Tro	S	Savanna	F	Bignoniaceae	Insect	40.49	142
1283	Tococa guianensis	WA	5.3N	77.3W	Tro	S	Forest	N	Melastomataceae	Insect	14.80	11
1284	Tococa coronata	WA	6.19N	62.49W	Tro	S	Shrubland	N & F	Melastomataceae	Insect	2.31	128
1285	Robinia pseudoacacia	WA	39.3N	77.44W	T	T	Unclear	F	Fabaceae	Insect	26.40	65

1286	Macaranga hosei	WA	3.21N	100.48E	Tro	T	Forest	N & F	Euphorbiaceae	Insect	13.38	54
1287	Acacia drepanolobium	WA	0.17N	37.52E	Tro	T	Savanna	N & F	Fabaceae	Insect	25.00	187
1288	Inga densiflora	WA	10.01N	83.45W	Tro	T	Forest	F	Fabaceae	Insect	8.75	101
1289	Tococa bullifera	WA	2.3S	60W	Tro	S	Forest	N & F	Melastomataceae	Insect	16.73	27
1290	Anemopaegma album	WA	15.27S	43.56W	Tro	S	Savanna	F	Bignoniaceae	Insect	35.08	142
1291	Mallotus japonicus	WA	34.41N	133.55E	T	T	Forest	F	Euphorbiaceae	Insect	21.62	202
1292	Coelogyne nitida	WA	27.55N	84.3E	T	H	Forest	F	Orchidaceae	Insect	27.00	191
1293	Picea abies	WA	67.55N	24.08E	C	T	Forest	F	Pinaceae	Insect	17.88	96
1294	Pluchea indica	WA	21.26N	157.47W	Tro	S	Coast	F	Asteraceae	Insect	31.30	14
1295	Tococa spadacijzora	WA	5.3N	77.3W	Tro	S	Forest	N	Melastomataceae	Insect	17.73	11
1296	Tococa guianensis	WA	6.2N	62.49W	Tro	S	Shrubland	N	Melastomataceae	Insect	2.75	128
1297	Populus tremuloides	WA	64.49N	147.59W	C	T	Forest	F	Salicaceae	Insect	35.58	136
1298	Banisteriopsis campestris	WA	18.59S	48.18W	Tro	S	Savanna	F	Malpighiaceae	Insect	5.00	12
1299	Piper cenocladum	WA	10.25N	84.05W	Tro	S	Forest	N & F	Piperaceae	Insect	18.70	46
1300	Macaranga tanarius	WA	3.14N	101.38E	Tro	T	Forest	N & F	Euphorbiaceae	Insect	5.00	55
1301	Macaranga conifera	WOA	3.14N	101.38E	Tro	T	Forest	NO	Euphorbiaceae	Insect	4.82	55
1302	Macaranga gigantea	WOA	3.14N	101.38E	Tro	T	Forest	NO	Euphorbiaceae	Insect	4.94	55
1303	Piper imperiale	WA	10.25N	84.05W	Tro	S	Forest	F	Piperaceae	Insect	9.60	57
1304	Piper cenocladum	WA	10.25N	84.05W	Tro	S	Forest	N & F	Piperaceae	Insect	4.50	57
1305	Tachigalia myrmecophila	WA	2.24S	W	Tro	T	Forest	N	Fabaceae	Insect	9.86	61
1306	Tococa sp.	WA	2.24S	W	Tro	T	Forest	N	Melastomataceae	Insect	27.73	61
1307	Duroia sp.	WA	2.24S	W	Tro	T	Forest	N	Rubiaceae	Insect	30.83	61
1308	Cordia nodosa	WA	2.24S	W	Tro	T	Forest	N & F	Boraginaceae	Insect	16.97	61
1309	Maieta guainensis	WA	2.24S	W	Tro	T	Forest	N & F	Melastomataceae	Insect	17.72	61
1310	Hirtella physophora	WA	2.24S	W	Tro	T	Forest	N & F	Chrysobalanaceae	Insect	22.90	61
1311	Duroia hirsuta	WA	3.37N	72.14W	Tro	T	Forest	N	Rubiaceae	Insect	10.10	63
1312	Macaranga tanarius	WA	2.29N	102.14E	Tro	T	Forest	N & F	Euphorbiaceae	Insect	5.90	85
1313	Macaranga kingii	WA	4.2N	113.5E	Tro	T	Forest	N & F	Euphorbiaceae	Insect	0.00	91
1314	Macaranga bavilandii	WA	4.2N	113.5E	Tro	T	Forest	N & F	Euphorbiaceae	Insect	2.00	91
1315	Piper cenocladum	WA	10.25N	84.5W	Tro	S	Forest	N & F	Piperaceae	Insect	10.61	109
1316	Pseudocedrela kotschyi	WA	8.44N	3.49W	Tro	T	Savanna	F	Meliaceae	Insect	3.47	130
1317	Rhizophora mucronata	WA	9.57N	98.37E	Tro	T	Mangrove	N & F	Rhizophoraceae	Insect	1.11	146
1318	Inga paraensis	WA	2.24S	59.44W	Tro	T	Forest	F	Fabaceae	Insect	39.00	181

1319	Cordia alliodora	WA	10.26N	83.59W	Tro	T	Forest	N & F	Boraginaceae	Insect	12.00	193
1320	Passiflora siamica	WA	21.41N	101.25E	Tro	H	Forest	F	Passifloraceae	Insect	6.70	201
1321	Passiflora coccinea	WA	21.41N	101.25E	Tro	H	Forest	F	Passifloraceae	Insect	7.40	201
1322	Populus tremuloides	AE	64.49N	147.59W	C	T	Forest	F	Salicaceae	Insect	63.93	136
1323	Tococa guianensis	AE	6.2N	62.49W	Tro	S	Shrubland	N	Melastomataceae	Insect	63.15	128
1324	Tococa spadacijzora	AE	5.3N	77.3W	Tro	S	Forest	N	Melastomataceae	Insect	58.12	11
1325	Pluchea indica	AE	21.26N	157.47W	Tro	S	Coast	F	Asteraceae	Insect	47.50	14
1326	Picea abies	AE	67.55N	24.08E	C	T	Forest	F	Pinaceae	Insect	45.18	96
1327	Coelogyne nitida	AE	27.55N	84.3E	T	H	Forest	F	Orchidaceae	Insect	44.00	191
1328	Mallotus japonicus	AE	34.41N	133.55E	T	T	Forest	F	Euphorbiaceae	Insect	40.00	202
1329	Anemopaegma album	AE	15.27S	43.56W	Tro	S	Savanna	F	Bignoniaceae	Insect	39.77	142
1330	Tococa bullifera	AE	2.3S	60W	Tro	S	Forest	N & F	Melastomataceae	Insect	37.56	27
1331	Inga densiflora	AE	10.01N	83.45W	Tro	T	Forest	F	Fabaceae	Insect	37.25	101
1332	Acacia drepanolobium	AE	0.17N	37.52E	Tro	T	Savanna	N & F	Fabaceae	Insect	28.00	187
1333	Macaranga hosei	AE	3.21N	100.48E	Tro	T	Forest	N & F	Euphorbiaceae	Insect	27.30	54
1334	Robinia pseudoacacia	AE	39.3N	77.44W	T	T	Unclear	F	Fabaceae	Insect	27.20	65
1335	Tococa coronata	AE	6.19N	62.49W	Tro	S	Shrubland	N & F	Melastomataceae	Insect	26.15	128
1336	Tococa guianensis	AE	5.3N	77.3W	Tro	S	Forest	N	Melastomataceae	Insect	26.13	11
1337	Anemopaegma scabriusculum	AE	15.27S	43.56W	Tro	S	Savanna	F	Bignoniaceae	Insect	25.78	142
1338	Solanum lycocarpum	AE	18.55S	48.15W	Tro	H	Savanna	F	Solanaceae	Insect	25.28	42
1339	Leonardoxa africana	AE	2.34N	9.5E	Tro	T	Forest	N & F	Fabaceae	Insect	23.74	71
1340	Macaranga trachyphylla	AE	4.2N	113.5E	Tro	T	Forest	N & F	Euphorbiaceae	Insect	22.45	91
1341	Macaranga bancana	AE	4.2N	113.5E	Tro	T	Forest	N & F	Euphorbiaceae	Insect	21.90	91
1342	Vaccinium myrtillus	AE	64N	20.2E	C	S	Forest	F	Ericaceae	Insect	20.00	13
1343	Macaranga tanarius	AE	3.21N	100.48E	Tro	T	Forest	N & F	Euphorbiaceae	Insect	19.78	54
1344	Inga punctata	AE	10.01N	83.45W	Tro	T	Forest	F	Fabaceae	Insect	19.63	101
1345	Turnera ulmifolia	AE	19.36N	96.22W	Tro	H	Sand dune	F	Turneraceae	Insect	18.88	192
1346	Hirtella myrmecophila	AE	2.25S	59.48W	Tro	T	Forest	N	Chrysobalanaceae	Insect	18.56	93
1347	Inga spectabilis	AE	0.4S	77.24W	Tro	T	Forest	F	Fabaceae	Insect	18.47	25
1348	Lafoensia pacari	AE	18.57S	48.12W	Tro	T	Savanna	F	Lythraceae	Insect	17.84	103
1349	Tococa guianensis	AE	13.8S	51.5W	Tro	T	Savanna	N & F	Melastomataceae	Insect	17.35	134
1350	Chrysothamnus viscidiflorus	AE	38.719N	106.816W	T	S	Grassland	F	Asteraceae	Insect	17.30	76
1351	Macaranga triloba	AE	3.14N	101.38E	Tro	T	Forest	N & F	Euphorbiaceae	Insect	16.03	55

1352	Populus fremontii	AE	40.45N	112.07W	C	T	Forest	F	Salicaceae	Insect	15.52	59
1353	Piper cenocladum	AE	10.25N	84.05W	Tro	S	Forest	N & F	Piperaceae	Insect	15.11	47
1354	Leonardoxa africana	AE	2.34N	9.5E	Tro	T	Forest	N & F	Fabaceae	Insect	14.77	72
1355	Solanum lycocarpum	AE	18.57S	48.12W	Tro	S	Savanna	F	Solanaceae	Insect	14.48	135
1356	Duroia hirsuta	AE	3.37N	72.14W	Tro	T	Forest	N	Rubiaceae	Insect	13.06	62
1357	Citrus sinensis	AE	10.29N	85.21W	Tro	T	Agricultural	N	Rutaceae	Insect	12.04	79
1358	Macaranga hosei	AE	4.2N	113.5E	Tro	T	Forest	N & F	Euphorbiaceae	Insect	12.03	91
1359	Macaranga hosei	AE	3.14N	101.38E	Tro	T	Forest	N & F	Euphorbiaceae	Insect	10.86	55
1360	Hirtella physophora	AE	5.03N	53.02W	Tro	S	Forest	N & F	Chrysobalanaceae	Insect	10.81	75
1361	Macaranga hypoleuca	AE	3.14N	101.38E	Tro	T	Forest	N & F	Euphorbiaceae	Insect	10.69	55
1362	Macaranga hosei	AE	3.14N	101.38E	Tro	T	Forest	N & F	Euphorbiaceae	Insect	10.13	55
1363	Quercus liaotungensis	AE	39.57N	115.25E	T	T	Forest	F	Fagaceae	Insect	10.10	205
1364	Macaranga bullettii	AE	4.2N	113.5E	Tro	T	Forest	N & F	Euphorbiaceae	Insect	10.00	91
1365	Tococa macrosperma	AE	6.21N	62.49W	Tro	S	Shrubland	N	Melastomataceae	Insect	10.00	128
1366	Cordia nodosa	AE	12.34S	70.05W	Tro	T	Forest	N & F	Boraginaceae	Insect	9.88	64
1367	Gossypium thurberi	AE	31.41N	110.57W	A	S	Sand dune	F	Malvaceae	Insect	8.94	166
1368	Tabebuia rosea	AE	9.19N	79.38W	A	T	Forest	F	Bignoniaceae	Insect	8.20	164
1369	Macaranga beccariana	AE	4.2N	113.5E	Tro	T	Forest	N & F	Euphorbiaceae	Insect	7.90	91
1370	Harungana madagascariensis	AE	1.19S	9.86E	Tro	T	Forest	F	Guttiferae	Insect	7.88	45
1371	Fallopia japonica	AE	43.02N	73.45W	C	H	Unclear	F	Polygonaceae	Insect	7.84	139
1372	Quercus dentata	AE	43.12N	141.15E	C	T	Coast	F	Fagaceae	Insect	7.70	92
1373	Gossypium hirsutum	AE	35.35N	85.29W	T	T	Agricultural	F	Malvaceae	Insect	7.70	190
1374	Theobroma cacao	AE	1.23S	121.26E	Tro	T	Agricultural	F	Malvaceae	Insect	7.42	199
1375	Cordia nodosa	AE	3.37N	72.14W	Tro	T	Forest	N & F	Boraginaceae	Insect	6.67	62
1376	Conocarpus erectus	AE	23.5N	75.8W	T	T	Mangrove	F	Combretaceae	Insect	5.63	155
1377	Cordia alliodora	AE	10.5N	85.36W	Tro	T	Forest	N & F	Boraginaceae	Insect	5.46	157
1378	Macaranga hulletti	AE	3.14N	101.38E	Tro	T	Forest	N & F	Euphorbiaceae	Insect	5.25	55
1379	Macaranga lamellata	AE	4.2N	113.5E	Tro	T	Forest	N & F	Euphorbiaceae	Insect	5.00	91
1380	Conocarpus erectus	AE	23.3N	75.47W	T	T	Mangrove	F	Combretaceae	Insect	4.96	154
1381	Pinus ponderosa	AE	39.06N	105.05W	T	T	Forest	F	Pinaceae	Insect	4.36	133
1382	Zanthoxylum myriacanthum	AE	3.17N	101.44E	Tro	T	Forest	N & F	Rutaceae	Insect	4.25	132
1383	Tococa formicaria	AE	13.53S	45.42W	Tro	T	Savanna	N	Melastomataceae	Insect	4.22	19
1384	Macaranga tanarius	AE	3.3N	101.31E	Tro	T	Forest	N & F	Euphorbiaceae	Insect	4.20	84

1385	Ouratea spectabilis	AE	18.59S	48.18W	Tro	T	Shrubland	F	Ochnaceae	Insect	4.03	29
1386	Polypodium plebeium	AE	19.32N	96.55W	Tro	F	Forest	F	Polypodiaceae	Insect	3.90	102
1387	Fallopia japonica	AE	42.53N	73.35W	C	H	Unclear	F	Polygonaceae	Insect	3.88	139
1388	Conocarpus erectus	AE	24.1N	76.26W	T	T	Mangrove	F	Combretaceae	Insect	3.26	153
1389	Macaranga winkleri	AE	4.2N	113.5E	Tro	T	Forest	N & F	Euphorbiaceae	Insect	3.13	91
1390	Mallotus japonicus	AE	34.41N	133.55E	T	T	Unclear	F	Euphorbiaceae	Insect	2.65	202
1391	Macaranga triloba	AE	3.3N	101.31E	Tro	T	Forest	N & F	Euphorbiaceae	Insect	2.60	84
1392	Coffea arabica	AE	15.15N	92.22W	Tro	T	Agricultural	N & F	Rubiaceae	Insect	2.53	151
1393	Rhizophora mucronata	AE	9.5N	98.35E	Tro	T	Mangrove	N & F	Rhizophoraceae	Insect	1.97	147
1394	Macaranga hosei	AE	3.3N	101.31E	Tro	T	Forest	N & F	Euphorbiaceae	Insect	1.70	84
1395	Cordia alliodora	AE	19.3N	105.02W	Tro	T	Forest	N & F	Boraginaceae	Insect	1.42	157
1396	Coffea arabica	AE	15.11N	92.2W	Tro	T	Agricultural	N & F	Rubiaceae	Insect	1.21	151
1397	Coffea arabica	AE	15.1N	92.19W	Tro	T	Agricultural	N & F	Rubiaceae	Insect	0.53	151
1398	Peixotoa tomentosa	WA	18.59S	48.18W	Tro	S	Savanna	F	Malpighiaceae	Insect	5.11	210
1399	Peixotoa tomentosa	AE	18.59S	48.18W	Tro	S	Savanna	F	Malpighiaceae	Insect	6.33	210
1400	Banisteriopsis laevifolia	WA	18.59S	48.18W	Tro	S	Savanna	F	Malpighiaceae	Insect	3.62	210
1401	Banisteriopsis laevifolia	AE	18.59S	48.18W	Tro	S	Savanna	F	Malpighiaceae	Insect	4.56	210
1402	Banisteriopsis campestris	WA	18.59S	48.18W	Tro	S	Savanna	F	Malpighiaceae	Insect	4.36	210
1403	Banisteriopsis campestris	AE	18.59S	48.18W	Tro	S	Savanna	F	Malpighiaceae	Insect	5.78	210
1404	Banisteriopsis malifolia	WA	18.59S	48.18W	Tro	S	Savanna	F	Malpighiaceae	Insect	7.27	210
1405	Banisteriopsis malifolia	AE	18.59S	48.18W	Tro	S	Savanna	F	Malpighiaceae	Insect	10.58	210

References (Four of the 210 references are based on the unpublished data source used in Turcotte et al. (2014)):

1 Abbott, I., Vanheurck, P., Burbidge, T. & Williams, M. Damage caused by insects and fungi to *Eucalypt Foliage* - spatial and temporal patterns in mediterranean forest of Western-Australia. *Forest Ecology and Management* **58**, 85-110 (1993).

2 Abdala-Roberts, L. & Marquis, R. J. Test of local adaptation to biotic interactions and soil abiotic conditions in the ant-tended *Chamaecrista fasciculata* (Fabaceae). *Oecologia* **154**, 315-326 (2007).

3 Adams, A. S. & Rieske, L. K. Herbivory and fire influence white oak (*Quercus alba* L.) seedling vigor. *Forest Science* **47**, 331-337 (2001).

4 Agrawal, A. A. Induced responses to herbivory in wild radish: Effects on several herbivores and plant fitness. *Ecology* **80**, 1713-1723 (1999).

5 Agrawal, A. A. & Kotanen, P. M. Herbivores and the success of exotic plants: a phylogenetically controlled experiment. *Ecology Letters* **6**, 712-715 (2003).

6 Agrawal, A. A. *et al.* Enemy release? An experiment with congeneric plant pairs and diverse above- and belowground enemies. *Ecology* **86**, 2979-2989 (2005).

7 Aide, T. M. & Zimmerman, J. K. Patterns of insect herbivory, growth, and survivorship in juveniles of a neotropical liana. *Ecology* **71**, 1412-1421 (1990).

8 Akiyama, R. & Agren, J. Magnitude and timing of leaf damage affect seed production in a natural population of *Arabidopsis thaliana* (Brassicaceae). *Plos One* **7** (2012).

9 Alcala, R. E., Mariano, N. A., Osuna, F. & Abarca, C. A. An experimental test of the defensive role of sticky traps in the carnivorous plant *Pinguicula moranensis* (Lentibulariaceae). *Oikos* **119**, 891-895 (2010).

10 Alonso, C. & Herrera, C. M. Variation in herbivory within and among plants of *Daphne laureola* (Thymelaeaceae): Correlation with plant size and architecture. *Journal of Ecology* **84**, 495-502 (1996).

11 Alvarez, G., Armbrecht, I., Jimenez, E., Armbrecht, H. & Ulloa-Chacon, P. Ant-plant association in two Tococa species from a primary rain forest of *Colombian Choco* (Hymenoptera : Formicidae). *Sociobiology* **38**, 585-602 (2001).

12 Alves-Silva, E. & Del-Claro, K. Effect of post-fire resprouting on leaf fluctuating asymmetry, extrafloral nectar quality, and ant-plant-herbivore interactions. *Naturwissenschaften* **100**, 525-532 (2013).

13 Atlegrim, O. Indirect effects of ant predation (Hymenoptera : Formicidae) on bilberry *Vaccinium myrtillus*. *European Journal of Entomology* **102**, 175-180 (2005).

14 Bach, C. E. Direct and Indirect Interactions between Ants (*Pheidole-Megacephala*), Scales (*Coccus-Viridis*) and Plants (*Pluchea-Indica*). *Oecologia* **87**, 233-239 (1991).

15 Balick, M. J., Furth, D. G. & Cooperdriver, G. Biochemical and evolutionary aspects of arthropod predation on ferns. *Oecologia* **35**, 55-89 (1978).

16 Barber, N. A. & Marquis, R. J. Light environment and the impacts of foliage quality on herbivorous insect attack and bird predation. *Oecologia* **166**, 401-409 (2011).

17 Bebber, D. P., Brown, N. D. & Speight, M. R. Dipterocarp seedling population dynamics in Bornean primary lowland forest during the 1997-8 El Nino-Southern Oscillation. *Journal of Tropical Ecology* **20**, 11-19 (2004).

18 Benitez-Malvido, J. & Kossmann-Ferraz, I. D. Litter cover variability affects seedling performance and herbivory. *Biotropica* **31**, 598-606 (1999).

19 Bizerril, M. X. A. & Vieira, E. M. Azteca ants as antiherbivore agents of *Tococa formicaria* (Melastomataceae) in Brazilian Cerrado. *Studies on Neotropical Fauna and Environment* **37**, 145-149 (2002).

20 Boege, K. Induced responses in three tropical dry forest plant species - direct and indirect effects on herbivory. *Oikos* **107**, 541-548 (2004).

21 Boege, K. Herbivore attack in *Casearia nitida* influenced by plant ontogenetic variation in foliage quality and plant architecture. *Oecologia* **143**, 117-125 (2005).

22 Boege, K. & Dirzo, R. Intraspecific variation in growth, defense and herbivory in *Dialium guianense* (Caesalpiniaceae) mediated by edaphic heterogeneity. *Plant Ecology* **175**, 59-69 (2004).

23 Bommarco, R., Miranda, F., Bylund, H. & Bjorkman, C. Insecticides suppress natural enemies and increase pest damage in cabbage. *Journal of Economic Entomology* **104**, 782-791 (2011).

24 Bray, J. R. Primary consumption in three forest canopies. *Ecology* **45**, 165-167 (1964).

25 Brenes-Arguedas, T., Coley, P. D. & Kursar, T. A. Divergence and diversity in the defensive ecology of Inga at two Neotropical sites. *Journal of Ecology* **96**, 127-135 (2008).

26 Bruelheide, H. & Scheidel, U. Slug herbivory as a limiting factor for the geographical range of Arnica montana. *Journal of Ecology* **87**, 839-848 (1999).

27 Bruna, E. M., Lapola, D. M. & Vasconcelos, H. L. Interspecific variation in the defensive responses of obligate plant-ants: experimental tests and consequences for herbivory. *Oecologia* **138**, 558-565 (2004).

28 Burgess, M. A. & Chapman, C. A. Tree leaf chemical characters: selective pressures by folivorous primates and invertebrates. *African Journal of Ecology* **43**, 242-250 (2005).

29 Byk, J. & Del-Claro, K. Nectar- and pollen-gathering Cephalotes ants provide no protection against herbivory: a new manipulative experiment to test ant protective capabilities. *Acta Ethologica* **13**, 33-38 (2010).

30 Cahill, J. F., Castelli, J. P. & Casper, B. B. The herbivory uncertainty principle: Visiting plants can alter herbivory. *Ecology* **82**, 307-312 (2001).

31 Campo, J. & Dirzo, R. Leaf quality and herbivory responses to soil nutrient addition in secondary tropical dry forests of Yucatan, Mexico. *Journal of Tropical Ecology* **19**, 525-530 (2003).

32 Carson, W. P. & Root, R. B. Top-down effects of insect herbivores during early succession: influence on biomass and plant dominance. *Oecologia* **121**, 260-272 (1999).

33 Cebrian, J. *et al.* Herbivory on posidonia oceanica: Magnitude and variability in the spanish mediterranean. *Marine Ecology Progress Series* **130**, 147-155 (1996).

34 Cepeda-Cornejo, V. & Dirzo, R. Sex-related differences in reproductive allocation, growth, defense and herbivory in three dioecious neotropical palms. *Plos One* **5** (2010).

35 Coley, P. D. *et al.* Divergent defensive strategies of young leaves in two species of inga. *Ecology* **86**, 2633-2643 (2005).

36 Cooke, F. P., Brown, J. P. & Mole, S. Herbivory, foliar enzyme-inhibitors, nitrogen and leaf structure of young and mature leaves in a tropical forest. *Biotropica* **16**, 257-263 (1984).

37 Cuevas-Reyes, P., Oyama, K., Gonzalez-Rodriguez, A., Fernandes, G. W. & Mendoza-Cuenca, L. Contrasting herbivory patterns and leaf fluctuating asymmetry in *Heliocarpus pallidus* between different habitat types within a Mexican tropical dry forest. *Journal of Tropical Ecology* **27**, 383-391 (2011).

38 Cuevas-Reyes, P., Quesada, M. & Oyama, K. Abundance and leaf damage caused by gall-inducing insects in a Mexican tropical dry forest. *Biotropica* **38**, 107-115 (2006).

39 Dawson, W., Burslem, D. F. R. P. & Hulme, P. E. Herbivory is related to taxonomic isolation, but not to invasiveness of tropical alien plants. *Diversity and Distributions* **15**, 141-147 (2009).

40 Delacruz, M. & Dirzo, R. A survey of the standing levels of herbivory in seedlings from a mexican rain-forest. *Biotropica* **19**, 98-106 (1987).

41 Delano-Frier, J. P. *et al.* The effect of exogenous jasmonic acid on induced resistance and productivity in amaranth (*Amaranthus hypochondriacus*) is influenced by environmental conditions. *Journal of Chemical Ecology* **30**, 1001-1034 (2004).

42 Del-Claro, K., Byk, J., Yugue, G. M. & Morato, M. G. Conservative benefits in an ant-hemipteran association in the Brazilian tropical savanna. *Sociobiology* **47**, 415-421 (2006).

43 del-Val, E. & Armesto, J. J. Seedling mortality and herbivory damage in subtropical and temperate populations: Testing the hypothesis of higher herbivore pressure toward the tropics. *Biotropica* **42**, 174-179 (2010).

44 do Nascimento, E. A. & Del-Claro, K. Ant visitation to extrafloral nectaries decreases herbivory and increases fruit set in *Chamaecrista debilis* (Fabaceae) in a Neotropical savanna. *Flora* **205**, 754-756 (2010).

45 Dunham, A. E. & Mikheyev, A. S. Influence of an invasive ant on grazing and detrital communities and nutrient fluxes in a tropical forest. *Diversity and Distributions* **16**, 33-42 (2010).

46 Dyer, L. A. & Letourneau, D. K. Relative strengths of top-down and bottom-up forces in a tropical forest community. *Oecologia* **119**, 265-274 (1999).

47 Dyer, L. A. & Letourneau, D. K. Trophic cascades in a complex terrestrial community. *Proceedings of the National Academy of Sciences of the United States of America* **96**, 5072-5076 (1999).

48 Edwards, P. J. Studies of mineral cycling in a montane rain-forest in New-Guinea .2. Production and disappearance of litter. *Journal of Ecology* **65**, 971-992 (1977).

49 Eichhorn, M. P., Compton, S. G. & Hartley, S. E. Seedling species determines rates of leaf herbivory in a Malaysian rain forest. *Journal of Tropical Ecology* **22**, 513-519 (2006).

50 Erickson, A. A., Bell, S. S. & Dawes, C. J. Does mangrove leaf chemistry help explain crab herbivory patterns? *Biotropica* **36**, 333-343 (2004).

51 Ermolaev, I. V. & Sidorova, O. V. Seasonal dynamics of damage to small-leaved lime trees by phyllophagous arthropods. *Entomological Review* **91**, 585-591 (2011).

52 Ewel, J. *et al.* Leaf area, light transmission, roots and leaf damage in nine tropical plant communities. *Agro-Ecosystems* **7**, 305-326 (1982).

53 Faveri, S. B., Vasconcelos, H. L. & Dirzo, R. Effects of Amazonian forest fragmentation on the interaction between plants, insect herbivores, and their natural enemies. *Journal of Tropical Ecology* **24**, 57-64 (2008).

54 Fiala, B., Grunsky, H., Maschwitz, U. & Linsenmair, K. E. Diversity of ant-plant interactions - protective efficacy in macaranga species with different degrees of ant association. *Oecologia* **97**, 186-192 (1994).

55 Fiala, B., Maschwitz, U., Pong, T. Y. & Helbig, A. J. Studies of a south east asian ant-plant association - protection of macaranga trees by *Crematogaster-borneensis*. *Oecologia* **79**, 463-470 (1989).

56 Filip, V., Dirzo, R., Maass, J. M. & Sarukhan, J. Within-year and among-year variation in the levels of herbivory on the foliage of trees from a mexican tropical deciduous forest. *Biotropica* **27**, 78-86 (1995).

57 Fincher, R. M. *et al.* Inter- and intraspecific comparisons of antiherbivore defenses in three species of rainforest understory shrubs. *Journal of Chemical Ecology* **34**, 558-574 (2008).

58 Fine, P. V. A. *et al.* The growth-defense trade-off and habitat specialization by plants in Amazonian forests. *Ecology* **87**, S150-S162 (2006).

59 Floate, K. D. & Whitham, T. G. Aphid-ant interaction reduces chrysomelid herbivory in a cottonwood hybrid zone. *Oecologia* **97**, 215-221 (1994).

60 Forkner, R. E. & Hunter, M. D. What goes up must come down? Nutrient addition and predation pressure on oak herbivores. *Ecology* **81**, 1588-1600 (2000).

61 Fowler, H. G. Herbivory and assemblage structure of myrmecophytous understory plants and their associated ants in the central amazon. *Insectes Sociaux* **40**, 137-145 (1993).
62 Frederickson, M. Ant species confer different partner benefits on two neotropical myrmecophytes. *Oecologia* **143**, 387-395 (2005).
63 Frederickson, M. E. & Gordon, D. M. The devil to pay: a cost of mutualism with Myrmelachista schumanni ants in 'devil's gardens' is increased herbivory on *Duroia hirsuta* trees. *Proceedings of the Royal Society B-Biological Sciences* **274**, 1117-1123 (2007).
64 Frederickson, M. E. *et al.* What happens when ants fail at plant defence? *Cordia nodosa* dynamically adjusts its investment in both direct and indirect resistance traits in response to herbivore damage. *Journal of Ecology* **101**, 400-409 (2013).
65 Fritz, R. S. in *Ecology* Vol. 64 789-797 (1983).
66 Fry, H. R. C., Quiring, D. I., Ryall, K. L. & Dixon, P. L. Relationships between elm spanworm, *Ennomos subsignaria*, juvenile density and defoliation on mature sycamore maple in an urban environment. *Forest Ecology and Management* **255**, 2726-2732 (2008).
67 Garcia-Gunman, G. & Benitez-Malvido, J. Effect of litter on the incidence of leaf-fungal pathogens and herbivory in seedlings of the tropical tree *Nectandra ambigens*. *Journal of Tropical Ecology* **19**, 171-177 (2003).
68 Garcia-Mari, F., Granda, C., Zaragoza, S. & Agusti, M. Impact of *Phyllocnistis citrella* (Lepidoptera : Gracillariidae) on leaf area development and yield of mature citrus trees in the Mediterranean area. *Journal of Economic Entomology* **95**, 966-974 (2002).
69 Garibaldi, L. A., Kitzberger, T. & Chaneton, E. J. Environmental and genetic control of insect abundance and herbivory along a forest elevational gradient. *Oecologia* **167**, 117-129 (2011).
70 Garibaldi, L. A., Kitzberger, T., Mazia, C. N. & Chaneton, E. J. Nutrient supply and bird predation additively control insect herbivory and tree growth in two contrasting forest habitats. *Oikos* **119**, 337-349 (2010).
71 Gaume, L. & McKey, D. An ant-plant mutualism and its host-specific parasite: activity rhythms, young leaf patrolling, and effects on herbivores of two specialist plant-ants inhabiting the same myrmecophyte. *Oikos* **84**, 130-144 (1999).
72 Gaume, L., McKey, D. & Anstett, M. C. Benefits conferred by ''timid'' ants: active anti-herbivore protection of the rainforest tree *Leonardoxa africana* by the minute ant *Petalomyrmex phylax*. *Oecologia* **112**, 209-216 (1997).
73 Giffard, B., Corcket, E., Barbaro, L. & Jactel, H. Bird predation enhances tree seedling resistance to insect herbivores in contrasting forest habitats. *Oecologia* **168**, 415-424 (2012).
74 Gonçalves-Alvim, S. J., Lana, T. C., Ranieri, B. D. & Fernandes, G. W. Test of hypotheses about herbivory and chemical defences of *Qualea parviflora* (Vochysiaceae) in Brazilian Cerrado. *Brazilian Journal of Botany* **34**, 223-230 (2011).
75 Grangier, J., Dejean, A., Male, P. J. G. & Orivel, J. Indirect defense in a highly specific ant-plant mutualism. *Naturwissenschaften* **95**, 909-916 (2008).
76 Grinath, J. B., Inouye, B. D., Underwood, N. & Billick, I. The indirect consequences of a mutualism: comparing positive and negative components of the net interaction between honeydew-tending ants and host plants. *Journal of Animal Ecology* **81**, 494-502 (2012).
77 Grubb, P. J. *et al.* Monocot leaves are eaten less than dicot leaves in tropical lowland rain forests: Correlations with toughness and leaf presentation. *Annals of Botany* **101**, 1379-1389 (2008).
78 Hamilton, J. G. *et al.* Insect herbivory in an intact forest understory under experimental CO2 enrichment. *Oecologia* **138**, 566-573 (2004).
79 Hammill, E., Corvalan, P. & Srivastava, D. S. Bromeliad-associated reductions in host herbivory: Do epiphytic bromeliads act as commensalists or mutualists? *Biotropica* **46**, 78-82 (2014).
80 Han, X. M., Dendy, S. P., Garrett, K. A., Fang, L. & Smith, M. D. Comparison of damage to native and exotic tallgrass prairie plants by natural enemies. *Plant Ecology* **198**, 197-210 (2008).
81 Hargrove, W. W., Crossley, D. A. & Seastedt, T. R. Shifts in insect herbivory in the canopy of black locust, *Robinia-pseudacacia*, after fertilization. *Oikos* **43**, 322-328 (1984).
82 Hartley, M. K., Rogers, W. E. & Siemann, E. Comparisons of arthropod assemblages on an invasive and native trees: abundance, diversity and damage. *Arthropod-Plant Interactions* **4**, 237-245 (2010).

83 Harvey, C. T. & Eubanks, M. D. Effect of habitat complexity on biological control by the red imported fire ant (Hymenoptera : Formicidae) in collards. *Biological Control* **29**, 348-358 (2004).
84 Heil, M., Fiala, B., Maschwitz, U. & Linsenmair, K. E. On benefits of indirect defence: short- and long-term studies of antiherbivore protection via mutualistic ants. *Oecologia* **126**, 395-403 (2001).
85 Heil, M. *et al.* Extrafloral nectar production of the ant-associated plant, Macaranga tanarius, is an induced, indirect, defensive response elicited by jasmonic acid. *Proceedings of the National Academy of Sciences of the United States of America* **98**, 1083-1088 (2001).
86 Hendrix, S. D. & Marquis, R. J. Herbivore damage to three tropical ferns. *Biotropica* **15**, 108-111 (1983).
87 Herbert, D. A., Mack, T. P., Backman, P. A. & Rodriguezkabana, R. Validation of a model for estimating leaf-feeding by insects in soybean. *Crop Protection* **11**, 27-34 (1992).
88 Hik, D. S., Brown, M., Dabros, A., Weir, J. & Cahill, J. F. Prevalence and predictability of handling effects in field studies: Results from field experiments and a meta-analysis. *American Journal of Botany* **90**, 270-277 (2003).
89 Hjalten, J. *et al.* Variable responses of natural enemies to *Salix triandra* phenotypes with different secondary chemistry. *Oikos* **116**, 751-758 (2007).
90 Horvitz, C. C. & Schemske, D. W. Effects of plant size, leaf herbivory, local competition and fruit production on survival, growth and future reproduction of a neotropical herb. *Journal of Ecology* **90**, 279-290 (2002).
91 Itino, T. & Itioka, T. Interspecific variation and ontogenetic change in antiherbivore defense in myrmecophytic Macaranga species. *Ecological Research* **16**, 765-774 (2001).
92 Ito, F. & Higashi, S. An indirect mutualism between oaks and wood ants via aphids. *Journal of Animal Ecology* **60**, 463-470 (1991).
93 Izzo, T. J. & Vasconcelos, H. L. Cheating the cheater: domatia loss minimizes the effects of ant castration in an Amazonian ant-plant. *Oecologia* **133**, 200-205 (2002).
94 Jackson, R. V., Kollmann, J., Grubb, P. J. & Bee, J. N. Insect herbivory on European tall-shrub species: the need to distinguish leaves before and after unfolding or unrolling, and the advantage of longitudinal sampling. *Oikos* **87**, 561-570 (1999).
95 Johnstone, I. M. Consumption of leaves by herbivores in mixed mangrove stands. *Biotropica* **13**, 252-259 (1981).
96 Karhu, K. J. Effects of ant exclusion during outbreaks of a defoliator and a sap-sucker on birch. *Ecological Entomology* **23**, 185-194 (1998).
97 Kerguelen, V. & Hoddle, M. S. Comparison of the susceptibility of several cultivars of avocado to the persea mite, *Oligonychus perseae* (Acari : Tetranychidae). *Scientia Horticulturae* **84**, 101-114 (2000).
98 Knepp, R. G. *et al.* Elevated CO2 reduces leaf damage by insect herbivores in a forest community. *New Phytologist* **167**, 207-218 (2005).
99 Knight, T. M., Caswell, H. & Kalisz, S. Population growth rate of a common understory herb decreases non-linearly across a gradient of deer herbivory. *Forest Ecology and Management* **257**, 1095-1103 (2009).
100 Knight, T. M. & Holt, R. D. Fire generates spatial gradients in herbivory: An example from a Florida sandhill ecosystem. *Ecology* **86**, 587-593 (2005).
101 Koptur, S. Experimental-evidence for defense of Inga (Mimosoideae) saplings by ants. *Ecology* **65**, 1787-1793 (1984).
102 Koptur, S., Rico-Gray, V. & Palacios-Rios, M. Ant protection of the nectaried fern *Polypodium plebeium* in central Mexico. *American Journal of Botany* **85**, 736-739 (1998).
103 Korndorfer, A. P. & Del-Claro, K. Ant defense versus induced defense in *Lafoensia pacari* (Lythraceae), a myrmecophilous tree of the brazilian cerrado. *Biotropica* **38**, 786-788 (2006).
104 Kouki, J. Small-scale distributional dynamics of the yellow water-lily and its herbivore *Galerucella-nymphaeae* (Coleoptera, Chrysomelidae). *Oecologia* **88**, 48-54 (1991).
105 Kudo, G. Variations in leaf traits and susceptibility to insect herbivory within a Salix miyabeana population under field conditions. *Plant Ecology* **169**, 61-69 (2003).
106 Kursar, T. A., Wolfe, B. T., Epps, M. J. & Coley, P. D. Food quality, competition, and parasitism influence feeding preference in a neotropical Lepidopteran. *Ecology* **87**, 3058-3069 (2006).
107 Lau, J. A., Strengbom, J., Stone, L. R., Reich, P. B. & Tiffin, P. Direct and indirect effects of CO2, nitrogen, and community diversity on plant-enemy interactions. *Ecology* **89**, 226-236 (2008).
108 Lawson, A. B. & Dahlsten, D. L. Evaluation of systemic insecticides as a treatment option in integrated pest management of the elm leaf beetle, *Xanthogaleruca luteola* (Muller) (Coleoptera : Chrysomelidae). *Journal of Economic Entomology* **96**, 1455-1462 (2003).

109 Letourneau, D. K. & Dyer, L. A. Experimental test in lowland tropical forest shows top-down effects through four trophic levels. *Ecology* **79**, 1678-1687 (1998).
110 Linit, M. J., Johnson, P. S., Mckinney, R. A. & Kearby, W. H. Insects and leaf-area losses of planted northern red oak seedlings in an ozark forest. *Forest Science* **32**, 11-20 (1986).
111 Liu, T. X. Population dynamics of *Bemisia argentifolii* (Homoptera : Aleyrodidae) on spring collard and relationship to yield in the Lower Rio Grande Valley of Texas. *Journal of Economic Entomology* **93**, 750-756 (2000).
112 Loch, A. D. & Matsuki, M. Effects of defoliation by *Eucalyptus weevil*, *Gonipterus scutellatus,* and chrysomelid beetles on growth of *Eucalyptus globulus* in southwestern Australia. *Forest Ecology and Management* **260**, 1324-1332 (2010).
113 Loe, G., Torang, P., Gaudeul, M. & Agren, J. Trichome production and spatiotemporal variation in herbivory in the perennial herb *Arabidopsis lyrata*. *Oikos* **116**, 134-142 (2007).
114 Loranger, H. *et al.* Invertebrate herbivory increases along an experimental gradient of grassland plant diversity. *Oecologia* **174**, 183-193 (2014).
115 Louda, S. M., Dixon, P. M. & Huntly, N. J. Herbivory in sun versus shade at a natural meadow-woodland ecotone in the rocky-mountains. *Vegetatio* **72**, 141-149 (1987).
116 Lowman, M. D. & Heatwole, H. Spatial and temporal variability in defoliation of australian eucalypts. *Ecology* **73**, 129-142 (1992).
117 Mack, A. L., Ickes, K., Jessen, J. H., Kennedy, B. & Sinclair, J. R. Ecology of *Aglaia mackiana* (Meliaceae) seedlings in a New Guinea rain forest. *Biotropica* **31**, 111-120 (1999).
118 MacKay, J. & Kotanen, P. M. Local escape of an invasive plant, common ragweed (*Ambrosia artemisiifolia* L.), from above-ground and below-ground enemies in its native area. *Journal of Ecology* **96**, 1152-1161 (2008).
119 Marquis, R. J. Leaf herbivores decrease fitness of a tropical plant. *Science* **226**, 537-539 (1984).
120 Marquis, R. J. Intra-crown variation in leaf herbivory and seed production in striped maple, *Acer-pensylvanicum* L (Aceraceae). *Oecologia* **77**, 51-55 (1988).
121 Marquis, R. J. & Clark, D. B. Habitat and fertilization effects on leaf herbivory in *Hampea-appendiculata* (Malvaceae) - implications for tropical firewood systems. *Agriculture Ecosystems & Environment* **25**, 165-174 (1989).
122 Marquis, R. J., Diniz, I. R. & Morais, H. C. Patterns and correlates of interspecific variation in foliar insect herbivory and pathogen attack in Brazilian cerrado. *Journal of Tropical Ecology* **17**, 127-148 (2001).
123 Marquis, R. J. & Whelan, C. J. Insectivorous birds increase growth of white oak through consumption of leaf-chewing insects. *Ecology* **75**, 2007-2014 (1994).
124 Massey, F. P., Massey, K., Press, M. C. & Hartley, S. E. Neighbourhood composition determines growth, architecture and herbivory in tropical rain forest tree seedlings. *Journal of Ecology* **94**, 646-655 (2006).
125 Mauricio, R. & Rausher, M. D. Experimental manipulation of putative selective agents provides evidence for the role of natural enemies in the evolution of plant defense. *Evolution* **51**, 1435-1444 (1997).
126 Mehltreter, K., Hülber., K. & Hietz., P. Herbivory on epiphytic ferns of a Mexican cloud forest. *Fern Gazette* **17**, 303-309 (2006).
127 Mehltreter, K. & Tolome, J. Herbivory on three tropical fern species of a Mexican cloud forest. P. 375-81 in S. Chandra and M. Srivastava, editors. Pteridology in the New Millenium. Kluwer Academic Publishers. (2008).
128 Michelangeli, F. A. Ant protection against herbivory in three species of Tococa (Melastomataceae) occupying different environments. *Biotropica* **35**, 181-188 (2003).
129 Mingaleva, N. A., Pestov, S. V. & Zagirova, S. V. Health status and biological damage to tree leaves in green areas of Syktyvkar. *Contemporary Problems of Ecology* **4**, 310-318 (2011).
130 Mody, K. & Linsenmair, K. E. Plant-attracted ants affect arthropod community structure but not necessarily herbivory. *Ecological Entomology* **29**, 217-225 (2004).
131 Moles, A. T. & Westoby, M. Do small leaves expand faster than large leaves, and do shorter expansion times reduce herbivore damage? *Oikos* **90**, 517-524 (2000).
132 Moog, J., Feldhaar, H. & Maschwitz, U. On the caulinary domatia of the SE-Asian ant-plant *Zanthoxylum myriacanthum* Wall. ex Hook. f. (Rutaceae) their influence on branch statics, and the protection against herbivory. *Sociobiology* **40**, 547-574 (2002).
133 Mooney, K. A. Tritrophic effects of birds and ants on a canopy food web, tree growth, and phytochemistry. *Ecology* **88**, 2005-2014 (2007).

134 Moraes, S. C. & Vasconcelos, H. L. Long-term persistence of a Neotropical ant-plant population in the absence of obligate plant-ants. *Ecology* **90**, 2375-2383 (2009).

135 Moreira, V. S. S. & Del-Claro, K. The outcomes of an ant-treehopper association on *Solanum lycocarpum* St. Hill: Increased membracid fecundity and reduced damage by chewing herbivores. *Neotropical Entomology* **34**, 881-887 (2005).

136 Mortensen, B., Wagner, D. & Doak, P. Defensive effects of extrafloral nectaries in quaking aspen differ with scale. *Oecologia* **165**, 983-993 (2011).

137 Myster, R. W. Foliar pathogen and insect herbivore effects on two landslide tree species in Puerto Rico. *Forest Ecology and Management* **169**, 231-242 (2002).

138 Nakamura, M., Kagata, H. & Ohgushi, T. Trunk cutting initiates bottom-up cascades in a tri-trophic system: sprouting increases biodiversity of herbivorous and predaceous arthropods on willows. *Oikos* **113**, 259-268 (2006).

139 Ness, J. H. *et al.* Reciprocally beneficial interactions between introduced plants and ants are induced by the presence of a third introduced species. *Oikos* **122**, 695-704 (2013).

140 Ness, J. H., Rollinson, E. J. & Whitney, K. D. Phylogenetic distance can predict susceptibility to attack by natural enemies. *Oikos* **120**, 1327-1334 (2011).

141 Nielsen, B. O. Above ground food resources and herbivory in a beech forest ecosystem. *Oikos* **31**, 273-279 (1978).

142 Nogueira, A., Guimaraes, E., Machado, S. R. & Lohmann, L. G. Do extrafloral nectaries present a defensive role against herbivores in two species of the family Bignoniaceae in a Neotropical savannas? *Plant Ecology* **213**, 289-301 (2012).

143 Norghauer, J. M., Malcolm, J. R. & Zimmerman, B. L. Juvenile mortality and attacks by a specialist herbivore increase with conspecific adult basal area of Amazonian *Swietenia macrophylla* (Meliaceae). *Journal of Tropical Ecology* **22**, 451-460 (2006).

144 Norghauer, J. M., Malcolm, J. R., Zimmerman, B. L. & Felfili, J. M. Experimental establishment of big-leaf mahogany (*Swietenia macrophylla* King) seedlings on two soil types in native forest of Para, Brazil. *Forest Ecology and Management* **255**, 282-291 (2008).

145 Offenberg, J., Havanon, S., Aksornkoae, S., MacIntosh, D. J. & Nielsen, M. G. Observations on the ecology of weaver ants (*Oecophylla smaragdina* Fabricius) in a Thai mangrove ecosystem and their effect on herbivory of *Rhizophora mucronata* Lam. *Biotropica* **36**, 344-351 (2004).

146 Offenberg, J., Macintosh, D. J. & Nielsen, M. G. Indirect ant-protection against crab herbivory: damage-induced susceptibility to crab grazing may lead to its reduction on ant-colonized trees. *Functional Ecology* **20**, 52-57 (2006).

147 Offenberg, J., Nielsen, M. G., Macintosh, D. J., Havanon, S. & Aksornkoae, S. Lack of ant attendance may induce compensatory plant growth. *Oikos* **111**, 170-178 (2005).

148 Ohmart, C. P., Stewart, L. G. & Thomas, J. R. Leaf consumption by insects in three Eucalyptus forest types in Southeastern Australia and their role in short-term nutrient cycling. *Oecologia* **59**, 322-330 (1983).

149 Pascual-Alvarado, E., Cuevas-Reyes, P., Quesada, M. & Oyama, K. Interactions between galling insects and leaf-feeding insects: the role of plant phenolic compounds and their possible interference with herbivores. *Journal of Tropical Ecology* **24**, 329-336 (2008).

150 Phillipson, J. & Thompson, D. J. Phenology and intensity of phyllophage attack on *Fagus-sylvatica* in wytham woods, Oxford. *Ecological Entomology* **8**, 315-330 (1983).

151 Philpott, S. M., Perfecto, I. & Vandermeer, J. Effects of predatory ants on lower trophic levels across a gradient of coffee management complexity. *Journal of Animal Ecology* **77**, 505-511 (2008).

152 Picoaga, A., Cartea, M. E., Soengas, P., Monetti, L. & Ordas, A. Resistance of kale populations to lepidopterous pests in northwestern Spain. *Journal of Economic Entomology* **96**, 143-147 (2003).

153 Piovia-Scott, J. The effect of disturbance on an ant-plant mutualism. *Oecologia* **166**, 411-420 (2011).

154 Piovia-Scott, J. Plant phenotype influences the effect of ant mutualists on a polymorphic mangrove. *Journal of Ecology* **99**, 327-334 (2011).

155 Piovia-Scott, J., Spiller, D. A. & Schoener, T. W. Effects of experimental seaweed deposition on lizard and ant predation in an island food web. *Science* **331**, 461-463 (2011).

156 Plath, M., Mody, K., Potvin, C. & Dorn, S. Establishment of native tropical timber trees in monoculture and mixed-species plantations: Small-scale effects on tree performance and insect

herbivory. *Forest Ecology and Management* **261**, 741-750 (2011).
157 Pringle, E. G., Dirzo, R. & Gordon, D. M. Indirect benefits of symbiotic coccoids for an ant-defended myrmecophytic tree. *Ecology* **92**, 37-46 (2011).
158 Pysek, P. Seasonal-changes in response of senecio-ovatus to grazing by the chrysomelid beetle *Chrysomela-speciosissima*. *Oecologia* **91**, 596-628 (1992).
159 Radho-Toly, S., Majer, J. D. & Yates, C. Impact of fire on leaf nutrients, arthropod fauna and herbivory of native and exotic eucalypts in Kings Park, Perth, Western Australia. *Austral Ecology* **26**, 500-506 (2001).
160 Reichle, D. E., Goldstei.Ra, Vanhook, R. I. & Dodson, G. J. Analysis of insect consumption in a forest canopy. *Ecology* **54**, 1076-1083 (1973).
161 Reynolds, B. C. & Crossley, D. A. Spatial variation in herbivory by forest canopy arthropods along an elevation gradient. *Environmental Entomology* **26**, 1232-1239 (1997).
162 Richards, L. A. & Coley, P. D. Seasonal and habitat differences affect the impact of food and predation on herbivores: a comparison between gaps and understory of a tropical forest. *Oikos* **116**, 31-40 (2007).
163 Richardson, S. J., Press, M. C., Parsons, A. N. & Hartley, S. E. How do nutrients and warming impact on plant communities and their insect herbivores? A 9-year study from a sub-Arctic heath. *Journal of Ecology* **90**, 544-556 (2002).
164 Riedel, J., Dorn, S., Brand, G., Barrios, H. & Mody, K. Effects of ants on arthropod assemblages of a native timber tree in a tropical reforestation plantation. *Journal of Applied Entomology* **137**, 418-428 (2013).
165 Robertson, A. I. & Duke, N. C. Insect herbivory on mangrove leaves in north queensland. *Australian Journal of Ecology* **12**, 1-7 (1987).
166 Rudgers, J. A. Enemies of herbivores can shape plant traits: Selection in a facultative ant-plant mutualism. *Ecology* **85**, 192-205 (2004).
167 Rudgers, J. A. & Hoeksema, J. D. Inter-annual variation in above- and belowground herbivory on a native, annual legume. *Plant Ecology* **169**, 105-120 (2003).
168 Rudgers, J. A. & Whitney, K. D. Interactions between insect herbivores and a plant architectural dimorphism. *Journal of Ecology* **94**, 1249-1260 (2006).
169 Rypstra, A. L. & Marshall, S. D. Augmentation of soil detritus affects the spider community and herbivory in a soybean agroecosystem. *Entomologia Experimentalis Et Applicata* **116**, 149-157 (2005).
170 Sanders, N. J., Belote, R. T. & Weltzin, J. F. Multitrophic effects of elevated atmospheric CO2 on understory plant and arthropod communities. *Environmental Entomology* **33**, 1609-1616 (2004).
171 Scherber, C., Milcu, A., Partsch, S., Scheu, S. & Weisser, W. W. The effects of plant diversity and insect herbivory on performance of individual plant species in experimental grassland. *Journal of Ecology* **94**, 922-931 (2006).
172 Schmidt, G. & Zotz, G. Herbivory in the epiphyte, *Vriesea sanguinolenta* Cogn. & Marchal (Bromeliaceae). *Journal of Tropical Ecology* **16**, 829-839 (2000).
173 Schnitzer, S. A., Reich, P. B., Bergner, B. & Carson, W. P. Herbivore and pathogen damage on grassland and woodland plants: a test of the herbivore uncertainty principle. *Ecology Letters* **5**, 531-539 (2002).
174 Schowalter, T. D. Invertebrate community structure and herbivory in a tropical rain-forest canopy in puerto-rico following hurricane hugo. *Biotropica* **26**, 312-319 (1994).
175 Schowalter, T. D. & Ganio, L. M. Vertical and seasonal variation in canopy arthropod communities in an old-growth conifer forest in southwestern Washington, USA. *Bulletin of Entomological Research* **88**, 633-640 (1998).
176 Schuldt, A. *et al.* Tree diversity promotes insect herbivory in subtropical forests of south-east China. *Journal of Ecology* **98**, 917-926 (2010).
177 Schuldt, A. *et al.* Plant traits affecting herbivory on tree recruits in highly diverse subtropical forests. *Ecology Letters* **15**, 732-739 (2012).
178 Shaw, D. C., Ernest, K. A., Rinker, H. B. & Lowman, M. D. Stand-level herbivory in an old-growth conifer forest canopy. *Western North American Naturalist* **66**, 473-481 (2006).
179 Shure, D. J. & Wilson, L. A. Patch-size effects on plant phenolics in successional openings of the southern appalachians. *Ecology* **74**, 55-67 (1993).
180 Siemann, E. & Rogers, W. E. Herbivory, disease, recruitment limitation, and success of alien and native tree species. *Ecology* **84**, 1489-1505 (2003).

181 Sinimbu, G., Coley, P. D., Lemes, M. R., Lokvam, J. & Kursar, T. A. Do the antiherbivore traits of expanding leaves in the Neotropical tree *Inga paraensis* (Fabaceae) vary with light availability? *Oecologia* **170**, 669-676 (2012).

182 Sipura, M. Tritrophic interactions: willows, herbivorous insects and insectivorous birds. *Oecologia* **121**, 537-545 (1999).

183 Sipura, M. Contrasting effects of ants on the herbivory and growth of two willow species. *Ecology* **83**, 2680-2690 (2002).19

184 Sobek, S., Scherber, C., Steffan-Dewenter, I. & Tscharntke, T. Sapling herbivory, invertebrate herbivores and predators across a natural tree diversity gradient in Germany's largest con2nected deciduous forest. *Oecologia* **160**, 279-288 (2009).

185 Spiller, D. A. & Agrawal, A. A. Intense disturbance enhances plant susceptibility to herbivory: Natural and experimental evidence. *Ecology* **84**, 890-897 (2003).

186 Spiller, D. A. & Schoener, T. W. Folivory on islands with and without insectivorous lizards: An eight-year study. *Oikos* **78**, 15-22 (1997).

187 Stanton, M. L. & Palmer, T. M. The high cost of mutualism: effects of four species of East African ant symbionts on their myrmecophyte host tree. *Ecology* **92**, 1073-1082 (2011).

188 Stoll, P., Dolt, C., Goverde, M. & Baur, B. Experimental habitat fragmentation and invertebrate grazing in a herbaceous grassland species. *Basic and Applied Ecology* **7**, 307-319 (2006).

189 Strong, A. M., Sherry, T. W. & Holmes, R. T. Bird predation on herbivorous insects: indirect effects on sugar maple saplings. *Oecologia* **125**, 370-379 (2000).

190 Styrsky, J. D. & Eubanks, M. D. A facultative mutualism between aphids and an invasive ant increases plant reproduction. *Ecological Entomology* **35**, 190-199 (2010).

191 Subedi, A. *et al.* Pollination and protection against herbivory of *Nepalese Coelogyninae* (Orchidaceae). *American Journal of Botany* **98**, 1095-1103 (2011).

192 Torres-Hernández, L., Rico-Gray, V., Castillo-Guevara, C. & Vergara, J. A. Effect of nectar-foraging ants and wasps on the reproductive fitness of *Turnera ulmifolia* (Turneraceae) in a coastal sand dune in Mexico. *Acta Zoologica Mexicana Nueva Serie* **81**, 13-21 (2000).

193 Trager, M. D. & Bruna, E. M. Effects of plant age, experimental nutrient addition and ant occupancy on herbivory in a neotropical myrmecophyte. *Journal of Ecology* **94**, 1156-1163 (2006).

194 Unsicker, S. B. & Mody, K. Influence of tree species and compass bearing on insect folivory of nine common tree species in the West African savanna. *Journal of Tropical Ecology* **21**, 227-231 (2005).

195 Van Bael, S. A. *et al.* General herbivore outbreak following an El Nino-related drought in a lowland Panamanian forest. *Journal of Tropical Ecology* **20**, 625-633 (2004).

196 Vasconcelos, H. L. & Davidson, D. W. Relationship between plant size and ant associates in two Amazonian ant-plants. *Biotropica* **32**, 100-111 (2000).

197 Vasquez, P. A., Grez, A. A., Bustamante, R. O. & Simonetti, J. A. Herbivory, foliar survival and shoot growth in fragmented populations of *Aristotelia chilensis*. *Acta Oecologica-International Journal of Ecology* **31**, 48-53 (2007).

198 Wagner, D., DeFoliart, L., Doak, P. & Schneiderheinze, J. Impact of epidermal leaf mining by the aspen leaf miner (*Phyllocnistis populiella*) on the growth, physiology, and leaf longevity of quaking aspen. *Oecologia* **157**, 259-267 (2008).

199 Wielgoss, A. *et al.* Interaction complexity matters: disentangling services and disservices of ant communities driving yield in tropical agroecosystems. *Proceedings of the Royal Society B: Biological Sciences* **281** (2014).

200 Winkler, M., Hulber, K., Mehltreter, K., Franco, J. G. & Hietz, P. Herbivory in epiphytic bromeliads, orchids and ferns in a Mexican montane forest. *Journal of Tropical Ecology* **21**, 147-154 (2005).

201 Xu, F. F. & Chen, J. Competition hierarchy and plant defense in a guild of ants on tropical Passiflora. *Insectes Sociaux* **57**, 343-349 (2010).

202 Yamawo, A., Tagawa, J., Hada, Y. & Suzuki, N. Different combinations of multiple defence traits in an extrafloral nectary-bearing plant growing under various habitat conditions. *Journal of Ecology* **102**, 238-247 (2014).

203 Yang, L. H. Pulses of dead periodical cicadas increase herbivory of American bellflowers. *Ecology* **89**, 1497-1502 (2008).

204 Zehnder, C. B., Stodola, K. W., Cooper, R. J. & Hunter, M. D. Spatial heterogeneity in the relative impacts of foliar quality and predation pressure on red oak, *Quercus rubra*, arthropod communities. *Oecologia* **164**, 1017-1027 (2010).

Zhang, S., Zhang, Y. X. & Ma, K. M. The ecological effects of ant-aphid mutualism on plants at a large spatial scale. *Sociobiology* **60**, 236-241 (2013).

Godfrey, Unpublished

Johnson, Unpublished

Johnson and Broadhead, Unpublished

Thomsen, Unpublished

Vilela, A. A., Torezan-Silingardi, H. M. & Del-Claro K. Conditional outcomes in ant–plant–herbivore interactions influenced by sequential flowering. Flora **209**:359-366 (2014).
